# Non-cognitive skills mediate education-related polygenic score associations with academic achievement across development

**DOI:** 10.1038/s41467-026-72838-2

**Published:** 2026-05-08

**Authors:** Quan Zhou, Wangjingyi Liao, Andrea G. Allegrini, Kaili Rimfeld, Jasmin Wertz, Tim T. Morris, Laurel Raffington, Robert Plomin, Margherita Malanchini

**Affiliations:** 1 Centre for Brain and Behaviour, School of Biological and Behavioural Sciences, Queen Mary University of London, London, UK; 2 Division of Psychology and Language Sciences, University College London, London, UK; 3 Social, Genetic & Developmental Psychiatry Centre, Institute of Psychiatry, Psychology & Neuroscience, King’s College London, London, UK; 4 Department of Psychology, Royal Holloway University of London, London, UK; 5 School of Philosophy, Psychology and Language Sciences, The University of Edinburgh, Edinburgh, UK; 6 Centre for Longitudinal Studies, Social Research Institute, University College London, London, UK; 7 Max Planck Research Group Biosocial – Biology, Social Disparities, and Development, Max Planck Institute for Human Development, Berlin, Germany

**Keywords:** Behavioural genetics, Human behaviour, Education

## Abstract

The role of environmental, developmental, and psychological processes in translating genetic dispositions into observed academic achievement remains under-investigated. Here, we examine whether non-cognitive skills—including motivation, attitudes, and emotional and behavioural functioning—mediate the genetic prediction of academic achievement across development. We analyse data from 5,016 children enrolled in the Twins Early Development Study at ages 7, 9, 12, and 16, as well as their parents and teachers. We find that non-cognitive skills mediate between less than 5 and up to 64% of the genetic prediction of academic achievement. Mediation effects are larger and more robust for motivation and attitudes (β ≈ 0.13) than for emotional and behavioural functioning (β ≈ 0.01–0.03). This pattern holds longitudinally and is replicated in within-family analyses, where non-cognitive skills account for up to 83% of the total mediation effects. These findings highlight the contribution of non-cognitive skills beyond shared familial factors, likely reflecting how children evoke and select experiences that align with their genetic propensity and lead to differences in academic development.

## Abstract (web summary)

The authors show that non-cognitive skills, including motivation and attitudes towards learning, mediate associations between education-related genetic disposition and academic achievement across compulsory education.

## Introduction

Academic achievement, commonly measured as school grades during childhood and adolescence, is associated with a range of positive life outcomes, including better physical and mental health^1,2^ and higher income^3–5^. Research indicates that genetic differences between people partly explain variation in academic achievement, a concept known as heritability^6^. Twin studies, which estimate the relative contribution of genetic and environmental factors by comparing identical and fraternal twins, have shown that 40 to 60% of the differences in educational attainment and academic achievement can be attributed to genetic factors^7,8^.

DNA-based methods have also quantified academic achievement, estimating heritability at 20 to 30% during childhood and adolescence^7,9,10^. Another way of capturing education-associated genetic dispositions is to construct a polygenic score^11^. Polygenic scores aggregate the effects of numerous genetic variants identified in large-scale genome-wide association studies, creating a composite index that captures known genetic disposition for a trait^12^. Education-associated polygenic scores have been found to account for 12 to 16% of the variance in adult educational attainment (years of schooling)^13^. In addition, a polygenic score combining education^14^ and cognition-associated genetic variants was found to account for 15% of the variance in academic achievement at the end of compulsory education in a UK-based sample^15^, a figure comparable to other well-established predictors of academic achievement, such as family socioeconomic status^16^.

Genetic associations with academic achievement likely encompass a combination of environmental, developmental, and psychological processes. To explore this further, recent studies investigated how family environments mediate these genetic influences. One study showed that, beyond socioeconomic status, multiple aspects of the family environment—such as supportive parenting, household chaos, and TV consumption—mediated polygenic score associations with academic achievement throughout compulsory education^17^. By employing a sibling-difference design to distinguish genetic associations shared between siblings (between-family effects) and those unique to each child (within-family effects), the study found that family environments were predominantly correlated with between-family factors^17^. These associations likely reflect passive gene-environment correlation, suggesting that children, by growing up with their biological parents, are exposed to environmental experiences that correlate with their genetics^18^.

Consistent with this interpretation, an adoption study that controls for passive gene–environment correlation by design, found no evidence that family environments such as warm parenting, household chaos, or time spent watching TV mediated education-linked genetic associations with children’s academic performance^19^. Another study found that mothers’ educational attainment  polygenic scores were associated with their children’s educational outcomes, beyond the children’s own genetics. This ‘genetic nurture’^20^ pathway from mother’s genetics to her child’s attainment is thought to capture environmental processes that covary with parental genetic propensities^21^ and was mediated by cognitively stimulating parenting^22^. However, environmental, developmental and psychological experiences other than family environments that may translate genetic propensity into academic achievement remain under-investigated.

The present study extends this line of enquiry and investigates whether another important set of psychological characteristics—non-cognitive skills—mediate polygenic score associations with academic achievement across development. The term ‘*non-cognitive skills*’ describes attitudes and characteristics that impact life outcomes beyond what cognitive tests can measure and predict^23^. These skills encompass motivation, perseverance, mindset, learning strategies, social skills, and self-regulatory strategies^24^. Non-cognitive skills are associated with educational outcomes beyond cognitive ability. Studies have found that self-efficacy and personality predict academic achievement beyond cognitive ability across compulsory education^25,26^. Other studies have linked personality, self-regulation, and motivation to academic performance^27–30^. More recently, our research highlighted that the association between non-cognitive skills and academic achievement increases substantially across compulsory education, from age 7 to 16^26^. Previous research also showed that greater self-control and, to a lesser extent, interpersonal skills, partly mediated the genetic prediction of adult educational attainment^31^. However, findings are mixed. For example, an adoption study found no evidence for the mediating role of inhibitory control, a more targeted facet of self-regulation, on educational performance in middle childhood^32^.

In the current study, we explore the role of non-cognitive skills in mediating polygenic score associations with academic achievement through five key questions. First, do non-cognitive skills mediate polygenic score associations with academic achievement across compulsory education? Second, can separating education-associated genetics into a cognitive and a non-cognitive component^26,33^ lead to the identification of partly distinct non-cognitive pathways related to academic achievement? Third, do non-cognitive skills mediate the genetic prediction of academic achievement over time, reflecting longitudinal mediation processes rather than concurrent associations? Fourth, do non-cognitive skills mediate polygenic score associations with academic achievement even when limited to differences within pairs of siblings? And lastly, is the mediating role of non-cognitive skills robust when controlling for other factors known to be related to both academic achievement and non-cognitive skills, namely general cognitive ability and family socio-economic status?

To address these questions, we employ a design with specific features. We measure several non-cognitive skills across development, including academic motivation, self-efficacy, and emotional and behavioural functioning at age 7, 9, 12, and 16 (see Methods). We select these constructs based on prior theoretical and empirical work that identified them as characteristics that predict academic achievement and engagement beyond cognitive performance^24,34^. We include data on non-cognitive skills from multiple raters –children, parents, and teachers. We consider polygenic scores associated with educational attainment (EA)^14^, as well as those associated with the cognitive and non-cognitive components of education^26^. This allows us to examine putatively different pathways from genetic disposition to observed academic achievement. We adopt a sibling difference design to partly adjust for shared sibling processes, such as passive gene-environment correlation^17,35^, demographic between-family factors, population stratification^36^, and assortative mating^37^.

We find that non-cognitive skills mediate associations between education-related genetic dispositions and academic achievement across compulsory education. Non-cognitive skills, particularly those closely linked to learning (e.g., academic interest, curiosity, and self-perceived ability), play an important role in translating education-associated genetic dispositions into observed academic outcomes. These mediation effects persist developmentally and are observed even after accounting for genetic and environmental differences between nuclear families. Although modest in magnitude, the effects are developmentally consistent and robust to controlling for potentially confounding factors such as family socio-economic status and general cognitive ability.

## Results

### Creating latent dimensions of non-cognitive skills

Different measures of non-cognitive skills were collected at different ages, with ratings provided by children, parents, and teachers. Following our previous work^26^, two latent dimensions of non-cognitive skills were created using confirmatory factor analyses: education-specific non-cognitive skills (including attitudes towards school, self-perceived academic ability, academic interest and curiosity) and emotional and behavioural functioning. A detailed description is provided in the Methods. Model fit indices are reported in Supplementary Data 25–26. While model fit was acceptable to good for most raters and timepoints, self- and parent-reported models at age 9 showed poorer fit, consequently results for these analyses at age 9 should be interpreted with caution.

### Correlations between polygenic scores, non-cognitive skills and academic achievement

Descriptive statistics are presented in Supplementary Note 1 and Supplementary Data 1. All variables were normally distributed; therefore, no transformations were applied prior to analyses.

We first analysed correlations of polygenic scores constructed from prior genome-wide association studies of educational attainment (*EA*^14^), cognitive (*Cog*) and non-cognitive (*NonCog*) skills, operationalised as genetic variants associated with education after accounting for genetic variants associated with cognitive performance^26^, with academic achievement and the latent non-cognitive dimensions rated by parents, teachers and self across compulsory education. These analyses were performed on unrelated individuals by selecting one twin from each pair randomly to account for relatedness. Correlations between polygenic scores and academic achievement at all ages were statistically significant (Supplementary Data 2a). Their magnitude tended to increase across development, particularly for the EA polygenic score (r ranging from 0.23 at age 7 to 0.35 at age 16) and the NonCog polygenic score (r ranging from 0.11 at age 7 to 0.24 at age 16). The correlations between polygenic scores and latent dimensions of non-cognitive skills were modest and statistically significant, except for self-rated non-cognitive skills at age 9 (see Supplementary Fig. 1**;** Supplementary Data 2b). The correlations between latent dimensions of non-cognitive skills and academic achievement were positive, moderately sized and statistically significant across all ages (r ranging between 0.13 and 0.63) (Supplementary Fig. 1**;** Supplementary Data 2b). Moderate and positive correlations were also observed when considering individual measures of non-cognitive skills, including academic interest, self-perceived ability, grit, and curiosity (see Supplementary Data 3).

### Non-cognitive skills mediate polygenic score associations with academic achievement

**Fig. 1:**
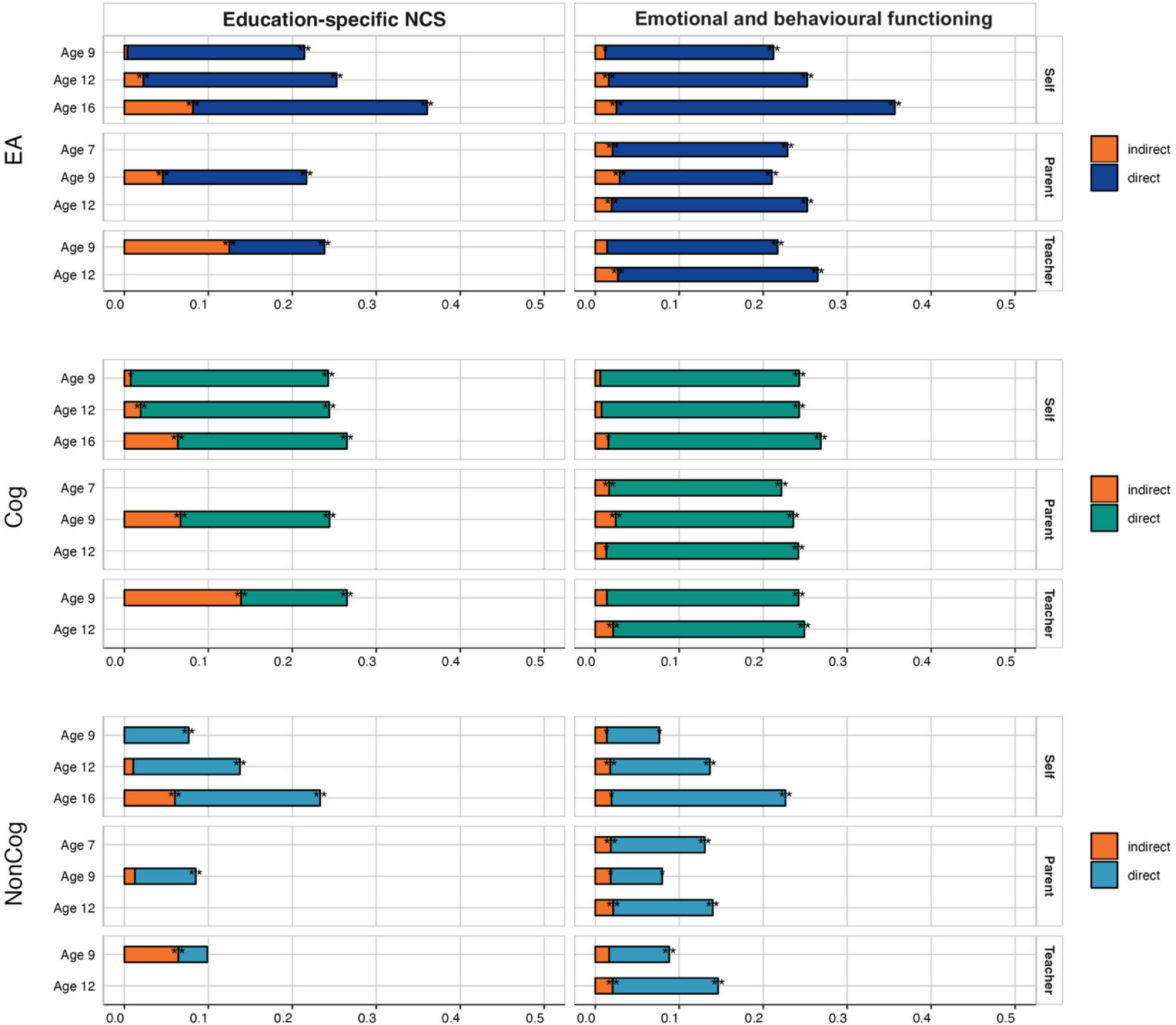
Polygenic score associations with academic achievement mediated by latent dimensions of education-specific non-cognitive skills and emotional and behavioural functioning. The length of each bar represents the total association (standardised beta coefficient) between the polygenic scores for educational attainment^14^ (EA; top panel), cognitive skills^26^ (Cog, middle panel) and non-cognitive skills^26^ (NonCog; bottom panel) and academic achievement at ages 7, 9, 12, and 16. The orange portion of each bar shows the indirect effect (i.e., mediated by latent factors of non-cognitive skills), while the remaining portion of each bar shows the direct effect of polygenic scores on academic achievement (i.e., not mediated by non-cognitive measures). The left panels show the mediation effects of  education-specific non-cognitive skills (NCS), while the right panels show mediation effects associated with emotional and behavioural functioning. *p < 0.05, **p < 0.01 after applying FDR correction (two-tailed). Model estimates and exact *N* are presented in Supplementary Data 4–6.

We applied mediation models to examine the extent to which latent dimension of non-cognitive skills (i.e., education-specific non-cognitive characteristics and emotional and behavioural functioning) mediated the association between genetic disposition towards education, measured using the educational attainment polygenic score, and academic achievement across compulsory education. Figure 1 (top panel) depicts the results of the mediation models for the educational attainment polygenic score. Indirect (mediated) effects for emotional and behavioural functioning were developmentally stable, similar across different raters, and small in magnitude, ranging from β = 0.01, *p* = 0.132 to β = 0.03, *p* < 0.001. In contrast, indirect (mediated) effects for education-specific non-cognitive skills increased across compulsory education, varied across raters, and were larger in magnitude. When considering self-reported measures, indirect (mediated) effects increased from zero at age 9, to β = 0.02 [95% CI, 0.01, 0.04], *p* < 0.001 at age 12 to β = 0.08 [95% CI, 0.06, 0.11], *p* < 0.001 at age 16. The indirect (mediated) effects of education-specific non-cognitive skills at age 9 were statistically significant for both parent-rated (β = 0.05 [95% CI, 0.02, 0.07], *p* < 0.001) and teacher-rated measures (β = 0.13 [95% CI, 0.10, 0.15], *p* < 0.001, all *p* values reported are adjusted using the Benjamini–Hochberg false discovery rate (FDR) method (see Fig. 1 top panel; Supplementary Data 4).

The two dimensions of education-specific non-cognitive skills and emotional and behavioural functioning also mediated the cognitive and non-cognitive polygenic score-academic achievement associations across compulsory education. The strongest mediation effects were observed for teacher-rated education-specific non-cognitive skills at age 9, which accounted for up to 48% of the total cognitive polygenic score prediction, and up to 64% of the total non-cognitive polygenic score prediction (Fig. 1 middle and bottom panels, and Supplementary Data 5 and 6). Sensitivity analyses considering a composite score including achievement in English and mathematics at ages 7, 9, and 12 yielded a similar pattern of results. The results were also highly consistent when we considered each subject separately (Supplementary Data7).

### A detailed analysis of education-specific non-cognitive measures as mediators of polygenic score associations with academic achievement

**Fig. 2:**
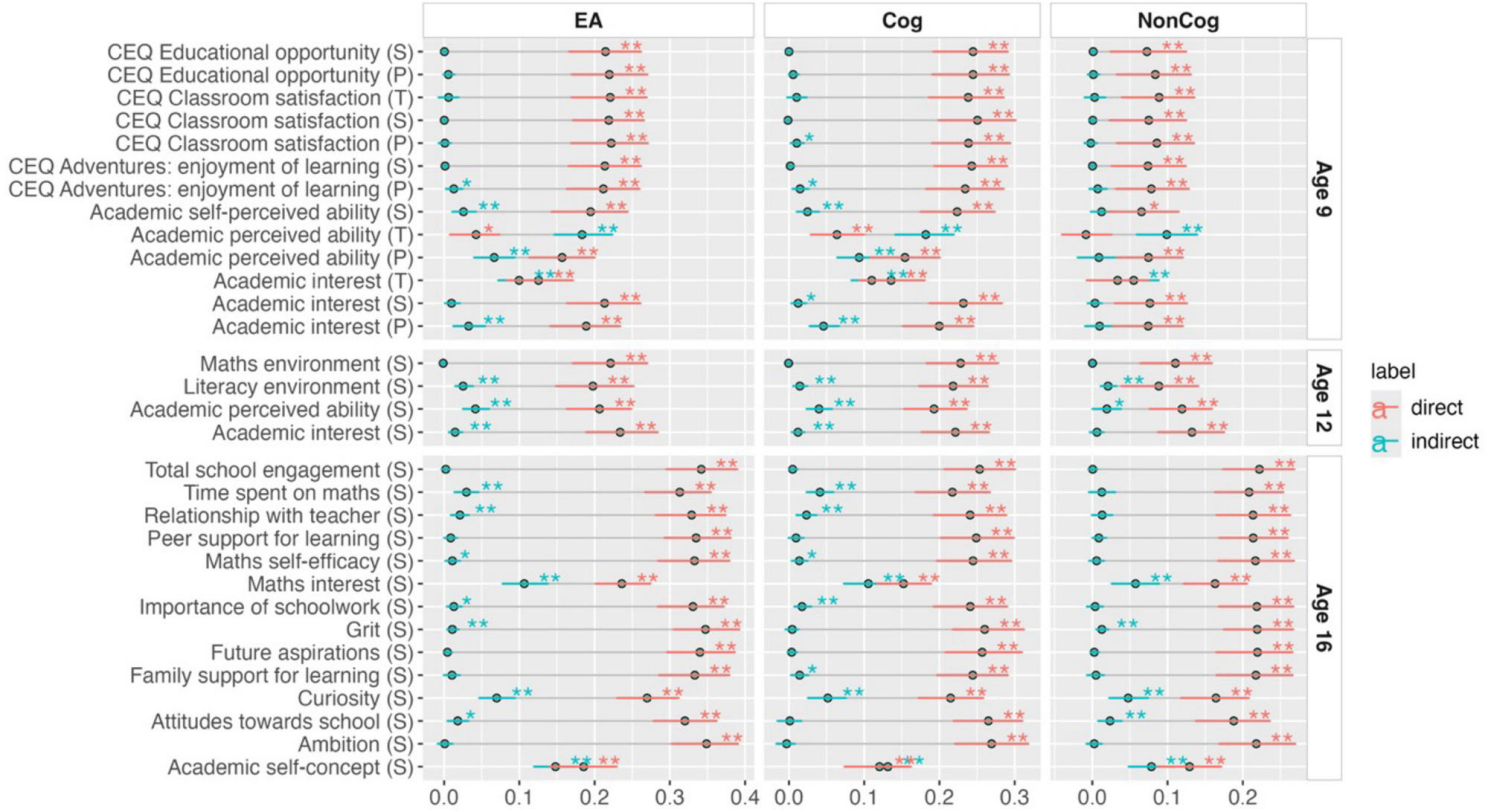
Direct and indirect effects of polygenic score associations with academic achievement as mediated by education-specific non-cognitive skills. Variables labelled with (P), (T), and (S) represent different informants: (P) = Parent-rated; (T) = Teacher-rated; (S) = Self-rated. Each dot represents the effect size for direct (red) and indirect (blue) polygenic score associations with academic achievement at ages 9, 12 and 16. EA = educational attainment polygenic score^14^ (left panel), Cog = cognitive polygenic score^26^ (middle panel), and NonCog = non-cognitive polygenic score^26^ (right panel). Error bars indicate 95% confidence intervals. Effects were estimated using mediation models (see Methods). All statistical tests are two-sided, and *P* values are adjusted using Benjamini–Hochberg false discovery rate (FDR). *p < 0.05, **p < 0.01 after applying FDR correction (two-tailed). Exact *P* values and sample sizes for each model are reported in Supplementary Data 8–10.

We conducted a more detailed set of analyses to investigate the role played by specific non-cognitive measures in the association between polygenic scores and academic achievements (Fig. 2 and Supplementary Data 8–10). Mediation effects were stronger for student-centred non-cognitive variables such as academic interest, academic perceived ability, self-concept and curiosity, as compared to measures capturing broader aspects of the learning environment, such as classroom satisfaction and peer support (see Fig. 2). In line with the results we obtained when considering latent non-cognitive dimensions, the mediating role of parent-rated and teacher-rated non-cognitive measures at age 9 was stronger than what we observed for self-rated non-cognitive measures. Although, for self-rated non-cognitive measures mediation effects increased developmentally, for example, for academic self-perceived ability and academic interest (Fig. 2).

By the end of compulsory education, the mediating role of self-rated non-cognitive measures was substantial. For example, the indirect (mediated) effects of academic self-concept and maths interests measured at age 16 accounted for 45% and 32% of the association between the EA polygenic score and academic achievement at age 16, respectively (left panel of Fig. 2). Moreover, when we partitioned polygenic score effects into cognitive and non-cognitive genetics, we observed that academic self-concept and interest mediated both polygenic score associations with academic achievement. For the Cog polygenic score, mediation effects accounted for 52% (self-concept) and 42% (interest) of the association with academic achievement (middle panel of Fig. 2); while for the NonCog polygenic score for 38% and 27%, respectively (right panel of Fig. 2). However, not all measures yielded significant effects. For example, measures capturing external perceptions of the school environment (e.g., CEQ classroom measures at age 9, and math environment at age 12) showed limited or nonsignificant mediation. Overall, mediation effects observed for individual non-cognitive measures were highly consistent across the three polygenic scores considered (Fig. 2 and Supplementary Data 8–10).

### Non-cognitive skills longitudinally mediate the associations between genetic dispositions and academic achievement

**Fig. 3:**
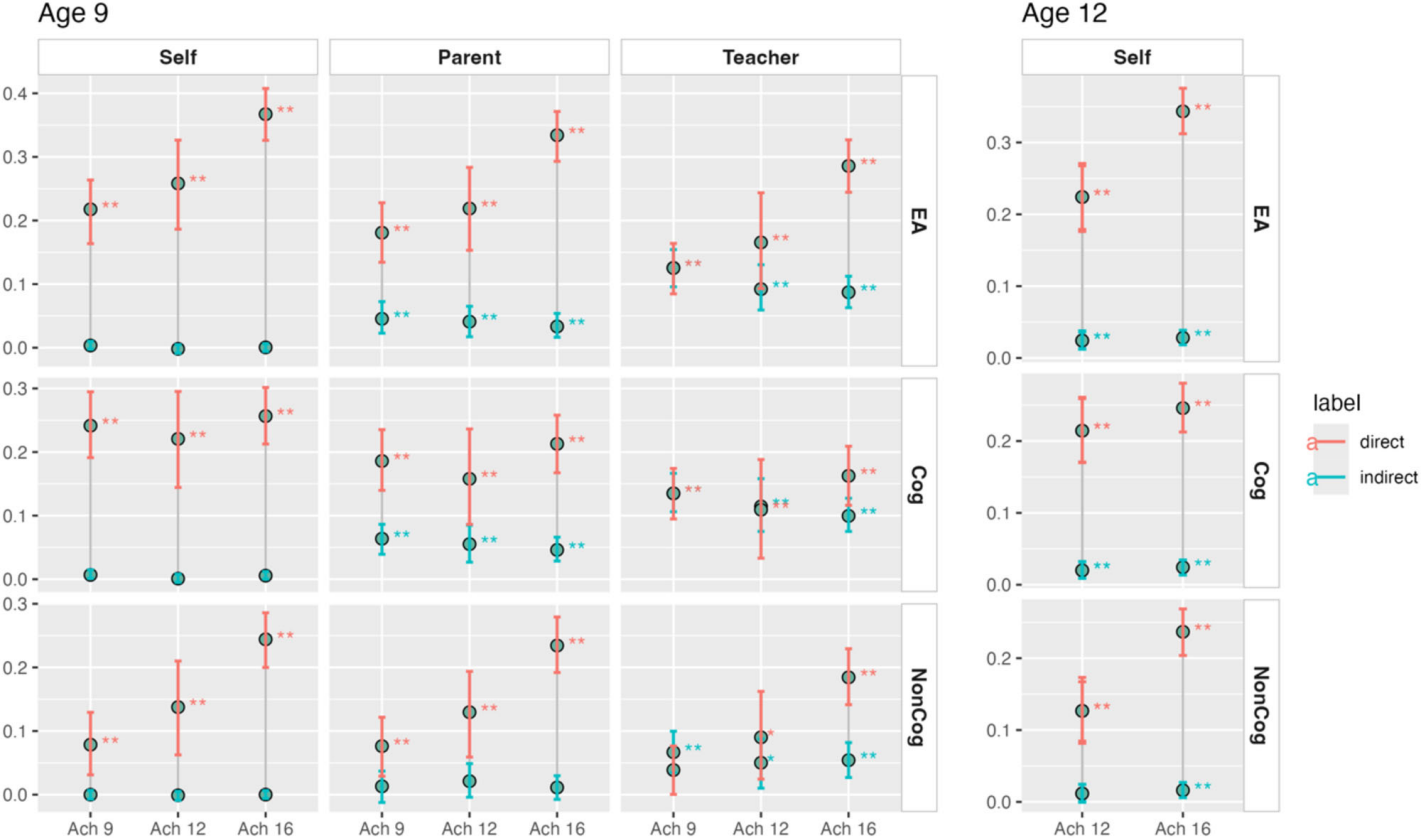
Time-lagged mediating effects of education-specific non-cognitive skills at age 9 (left panel) and age 12 (right panel) on the polygenic score (PGS) predictions of academic achievement across development. The colours represent the direct (red) and indirect (blue) effects (standardised beta coefficients) of the educational attainment^14^ (EA; top panel), cognitive^26^ (Cog) and non-cognitive^26^ (NonCog; bottom panel) polygenic scores on academic achievement at ages 9 (Ach 9), 12 (Ach 12), and 16 (Ach 16). Error bars indicate 95% confidence intervals. Effects were estimated using mediation models (see Methods). All statistical tests are two-sided, and *P* values are adjusted using the Benjamini–Hochberg false discovery rate (FDR) procedure. *p < 0.05, **p < 0.01 after applying FDR correction (two-tailed). Exact *P* values and sample sizes for each model are reported in Supplementary Data 11–12.

Given the consistent mediation effects of education-specific non-cognitive skills that emerged from our cross-sectional analyses, we next examined time-lagged effects longitudinally, for example, whether non-cognitive skills measured at age 9 mediated polygenic score associations with academic achievement measured at ages 12 and 16. We observed persistent mediation effects even in these time-lagged analyses, although effects were slightly attenuated in some, but not all, cases (Fig. 3). For example, the role of self-rated non-cognitive skills measured at age 12 as mediator of the association between educational attainment polygenic score and academic achievement measured at age 16 remained substantial (right panel of Fig. 3). Findings were highly consistent when we examined Cog and NonCog polygenic score effects (see middle and bottom panel of Fig. 3 and Supplementary Data 11) and for individual non-cognitive measures (see Supplementary Data 12).

### Within-family mediation analyses

We applied multi-level mediation models (see Methods) to separate polygenic score associations into within- and between-family effects and further disentangle the processes underlying the role of non-cognitive skills in these associations. The number of sibling pairs included in the analyses ranged between 1104 and 2895, depending on the data collection wave and measures included. Family-level intraclass correlations (ICCs) for latent non-cognitive factors ranged between 0.23 and 0.52 (see Supplementary Data 13), indicating moderate to substantial family-related effects on the development of non-cognitive skills.

**Fig. 4:**
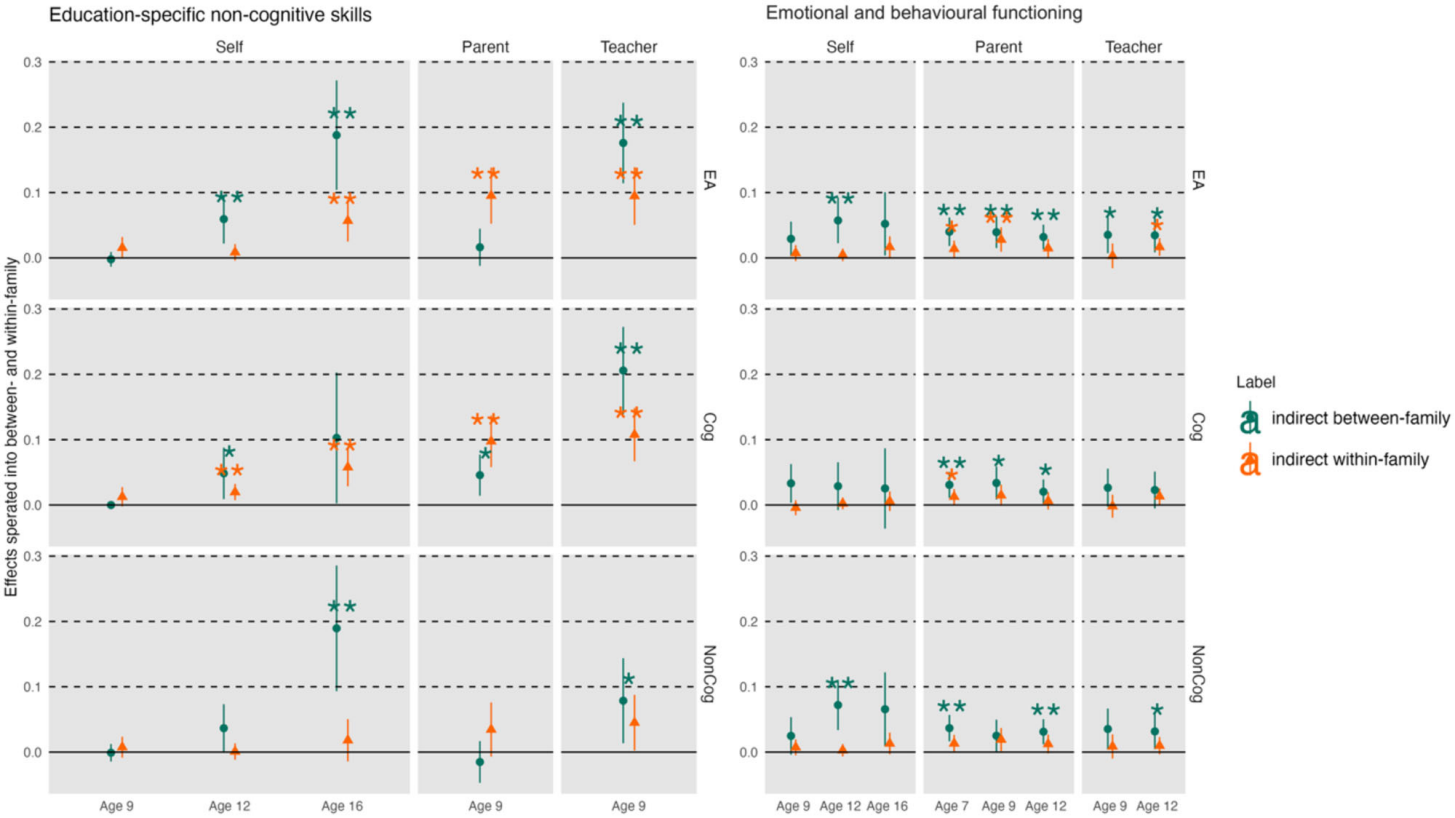
Non-cognitive mediation of the polygenic score predictions of academic achievement between- and within-families. Each dot represents the effect of the polygenic score prediction (standardised beta coefficient) of academic achievement across development (ages 7, 9, 12 and 16) separated into between- and within-family effects. Each error bar indicates 95% confidence intervals.  The left panel depicts the results for latent factors of education specific non-cognitive skills, while the right panel represents those for latent factors of emotional and behavioural functioning. Educational attainment polygenic score (EA)^14^: top panel. Cognitive polygenic score (Cog)^26^: middle panel. Non-cognitive polygenic score (NonCog)^26^: bottom panel. Effects were estimated using *1-1-1* two-levels mediation models (see Methods). All statistical tests are two-sided, and *P* values are adjusted using the Benjamini–Hochberg false discovery rate (FDR) procedure. *p < 0.05, **p < 0.01 after applying FDR correction (two-tailed). Exact *P* values and sample sizes for each model are reported in Supplementary Data 14–17.

Consistent with previous research^13,26,38^, we found that the associations between the educational attainment polygenic score and academic achievement across development were attenuated when looking at differences within families. However, young people’s non-cognitive skills remained significant mediators of these associations even when comparing siblings (Fig. 4 top panel and Supplementary Data 14). Siblings with a higher education-related polygenic score showed on average greater academic achievement, and this association was partly accounted for by higher levels of non-cognitive skills. Notably, our finding of a developmental increase in the mediating role of self-reported non-cognitive skills replicated at the within-family level (see left panel of Fig. 4).

For parent-rated non-cognitive skills, mediation effects were mainly evident at the within-family level rather than between-families (i.e., between-family mediation effect: β = 0.02 [95% CI, −0.01, 0.05], *p* = 0.349; within-family mediation effect: β = 0.10 [95% CI, 0.05, 0.14], *p* < 0.001), accounting for 83% of the total mediation effects. Polygenic score associations with academic achievement that are unique to each child, rather than shared by siblings, were more likely to be mediated by parents’ perceptions of non-cognitive profiles unique to each child. While this was observed for education-specific non-cognitive skills, associations were different for emotional and behavioural functioning. When considering children’s unique emotional and behavioural functioning profiles, most mediation effects were attenuated and did not reach significance in within-family analyses. We observed a similar pattern of results when repeating mediation analyses separating education-related genetics into cognitive and non-cognitive polygenic scores (middle and bottom panel of Fig. 4; Supplementary Data 15 and 16). Results were also consistent when we examined the mediating role of each education-specific non-cognitive skill individually (Supplementary Data 17).

We further investigated whether within-family mediation effects could be observed longitudinally. We found that time-lagged mediation effects for education-specific non-cognitive measures persisted even when looking within families. For example, parent-rated education-specific non-cognitive skills measured at age 9 mediated the educational atainment polygenic score associations with academic achievement measured at both age 12 (β = 0.08 [95% CI, 0.03, 0.12], *p* = 0.004) and age 16 (β = 0.08 [95% CI, 0.05, 0.11], *p* < 0.001). The effect sizes were comparable to those observed cross-sectionally in the association with academic achievement measured at age 9 (that is, β = 0.10 [95% CI, 0.05, 0.14], *p* < 0.001). The full results are presented in Supplementary Data 18–20 and provide evidence for the long-lasting mediating role of education-specific non-cognitive skills in the association between polygenic score and achievement across compulsory education. When examining the effects of each education-related non-cognitive skill individually, we found that parent-rated academic perceived ability and academic interest evinced the strongest mediation effects (β = 0.09 [95% CI, 0.04, 0.15], *p* < .001 and β = 0.08 [95% CI, 0.05, 0.11], *p* < 0.001, see Supplementary Data 21).

### Adjusting for general cognitive ability (g) and family socio-economic status (SES)

In the current study, we considered ‘non-cognitive’ those measures of behaviours, attitudes and motivation that have been found to relate to academic outcomes beyond performance in cognitive tests^26,34,39^. However, because previous research has shown that students’ non-cognitive skills correlate with students’ cognitive ability^26,28^, we tested the possibility that cognitive ability could account for the observed mediation effects. Specifically, we repeated our mediation analyses, including general cognitive ability as a second mediator (see Methods). Results showed that non-cognitive skills remained significant, although slightly attenuated, mediators of the polygenic scores-achievement associations (Supplementary Fig. 3 and Supplementary Data 22). Results were similar across compulsory education and across the different polygenic scores (Supplementary Figs. 4 and 5; Supplementary Data 22). Non-cognitive skills also remained significant mediators after accounting for family socio-economic status (Supplementary Fig. 6 and Supplementary Data 23), with similar results observed across polygenic scores and developmentally (Supplementary Figs. 7 and 8; Supplementary Data 23).

## Discussion

While it is well-established that genetic differences between students partly explain variation in academic achievement^6,7^, how they manifest remains under-investigated. To address this gap in our knowledge, the present study provides an in-depth examination of the role that *non-cognitive skills* (e.g., academic motivation, attitudes and goals) play in the association between genetic disposition and observed academic achievement across compulsory education. Our analyses revealed three key findings. First, non-cognitive skills, particularly those skills closely linked to learning (e.g., academic interest, curiosity, and academic self-perceived ability), play a critical role in translating education-associated genetic dispositions into observed academic achievement. Second, our time-lagged analyses showed that the mediating role of non-cognitive skills persisted throughout development. Third, these mediation effects could also be observed when examining differences between siblings. Mediation effects were small yet consistent and robust to adjusting for family socio-economic status and general cognitive ability, highlighting the independent contribution of non-cognitive skills to academic success.

Our cross-sectional analyses showed that the latent dimensions of education-specific non-cognitive skills and emotional and behavioural functioning both mediated the associations between education-related genetic dispositions and academic performance. However, while the role of emotional and behavioural functioning was stable across compulsory education, the mediating role of education-specific non-cognitive skills increased developmentally, pointing to the growing importance of students’ perceived non-cognitive profiles and experiences in their academic journeys. This developmental increase is consistent with the possibility that, as they grow up, children become more aware of their aptitudes and appetites towards learning. As they gain greater self-awareness and autonomy, students might become increasingly more able to shape their environmental contexts in ways that allow them to cultivate these non-cognitive skills and, in turn, foster their academic performance^40^.

The magnitude of mediation effects also differed depending on raters. Teacher reports of education-specific non-cognitive skills evinced the strongest effects, followed by parent reports, and self-reports. These stronger effects may reflect rater bias, as teachers evaluated both students’ performance and their learning-related attitudes^41^. However, they may also reflect the greater expertise and objectivity that characterise teachers’ assessments^42^. This is supported by our finding that teacher-rated non-cognitive skills had long-lasting mediation effects in our time-lagged analyses, since different teachers assessed achievement at different ages. Teachers have first-hand experience of how children express their attitudes and appetites within academic settings and might, therefore, be able to capture students’ non-cognitive competencies related to learning more accurately^43^.

In addition to latent factors that capture shared variance across education-specific characteristics and emotional and behavioural functioning, we also examined individual non-cognitive measures. We conducted a set of fine-grained analyses to identify which aspects of non-cognitive skills mediated polygenic score associations with academic achievement across development. This offered a more detailed view of construct-specific associations and reduced the risk of overinterpreting the results emerging from broad latent dimensions. First, we observed that specific student-centred academic attitudes and perceptions, such as academic interest, curiosity, and perceived ability, evinced stronger mediation effects as compared to perceptions of the learning environment that were external to each individual, such as perception of the classroom or the learning environments. These findings align with previous work, which emphasised the critical role of intrinsic, student-centred non-cognitive skills (e.g., intellectual curiosity and academic self-concept) in gene-environment transactions^40,44^, driving students to actively engage in academic settings beyond what environmental factors alone can achieve^45^.

We examined whether the mediating role of non-cognitive skills was more strongly linked to cognitive or non-cognitive polygenic scores. We found that mediation effects were comparable when separating education-related genetic variants into cognitive and non-cognitive polygenic scores. Interestingly, for some student-centred non-cognitive skills such as academic interest and academic self-concept, mediating effects were stronger for the path from cognitive genetics to academic achievement. This is consistent with observations of moderate associations between cognitive and non-cognitive skills across compulsory education. In fact, although the term *non-cognitive* skills was coined to highlight their independent contribution to academic performance beyond cognitive skills, the two constructs are related and developmentally intertwined. These results also align with propositions that non-cognitive skills like interest, curiosity and self-discipline help students leverage their cognitive competencies more effectively, therefore facilitating academic performance^46^.

Our time-lagged analysis confirmed that the mediating role of non-cognitive skills lasted throughout compulsory education. These findings reinforce the pivotal role of non-cognitive skills in translating genetic dispositions into academic achievement, and underscore the importance of fostering non-cognitive skills to enhance students’ long term academic outcomes, consistent with previous studies on the effectiveness of interventions for improving non-cognitive skills. For instance, interventions focusing on self-control and social skills at age 7 were found to have a positive impact on academic achievement in early adulthood^47^. A recent systematic review and meta-analysis showed that early-life interventions aimed at improving self-regulation and perseverance were associated with modest improvements in later-life academic and psychosocial outcomes^48^.

Our within-family analyses provided us with a more nuanced understanding of the potential mechanisms through which genetic dispositions and non-cognitive experiential processes combine and contribute to differences between students in academic achievement. Examining differences between siblings, dizygotic twins in this case, we were able to separate the effects of family-wide processes from those processes that are unique to each child within a family. We found that the mediating role of non-cognitive skills could be observed not only between-families, but also when looking at differences between siblings, accounting for a substantial portion of the total association. Children’s unique genetic dispositions were associated with academic achievement partly through their non-cognitive profiles. These effects emerged beyond family-shared genetic, environmental and demographic factors such as assortative mating^37^, population stratification^36^, and passive gene-environment correlation. This underscores the key role played by individual-specific non-cognitive profiles in propelling children down different learning pathways. Our findings are in line with transactional models of human development rooted in evocative/active gene-environment correlation^40,49^ that propose that, partly in line with their genetic dispositions, children actively seek out different environmental experiences on the basis of their non-cognitive characteristics (e.g., interests, preferences, and aptitudes), and in turn, these will lead to different learning outcomes.

A number of limitations should be acknowledged. First, the reliance on polygenic scores as proxies for genetic influences on academic achievement is an inherent constraint, as these scores are imperfect representations of the complex genetic architecture underlying academic performance^14,50^. This is particularly relevant to our non-cognitive polygenic score analyses, since this score is indirectly constructed by isolating those genetic variants related to education that are not associated with cognitive skills, and not directly from a GWAS of actual non-cognitive measures^26^. It would be of interest to replicate these results when adequately powered GWAS of non-cognitive skills will become available. Second, our non-cognitive measures may not fully capture the breadth and depth of factors influencing individuals’ non-cognitive profiles relevant to academic success^24,51^. Extant literature on the links between non-cognitive skills and educational outcomes is complex and characterised by inconsistencies, low-quality studies and publication bias^48^. Greater clarity regarding the taxonomy of non-cognitive skills that relate to education, better measures, and high-quality longitudinal studies are urgently needed.

A third limitation concerns the heterogeneity of the constructs grouped under the umbrella of non-cognitive skills. While this label has been widely used in both the psychological and economic literature, it encompasses a widely diverse set of characteristics, ranging from motivational beliefs and self-regulatory strategies to attitudes and perceptions of the learning environment, that differ in their specificity, developmental stability, and contextual embedding^24,34^. For instance, classroom satisfaction may reflect environmental quality as well as a dispositional characteristic. We retained the term ‘non‑cognitive skills’ for consistency with prior work and to facilitate comparison with the extant literature^24,26,34^, but we acknowledge its limitations. In fact, one of our goals was to empirically examine which of these constructs serve as meaningful mediators of the polygenic score prediction of academic achievement, to inform future efforts toward a clearer taxonomy of education‑related non‑cognitive characteristics.

Fourth, to improve measurement reliability and reduce error, we employed latent factor models that captured shared variance across conceptually related constructs. This strategy was based on prior validation using the same dataset^26^, where latent composites showed stronger predictive utility than individual non-cognitive indicators—many of which are known to exhibit low reliability and substantial response heterogeneity^52^. However, we also acknowledge that model fit varied across timepoints, with relatively poor fit observed for some models, particularly at younger ages (e.g., age 9 self- and parent-reported constructs; see Supplementary Data 25–26). These discrepancies likely reflect both developmental variability and limited item coverage^53^. As such, we reported results based on both latent factors and individual indicators (see Figs. 1 and 2). The SDQ was used as a latent factor capturing broad emotional and behavioural functioning—consistent with its treatment in prior economics-based research^39,54^, rather than a narrow self-regulation measure. Future work might explore alternative structures (e.g., bifactor models) and develop developmentally sensitive, structurally valid measures of non-cognitive characteristics that allow for more detailed cross-age and cross-context analyses.

Fifth, we cannot exclude potential genetic confounding in our mediation analyses. Mediation pathways may be under-corrected for genetic influences in phenotypic variables^55–57^, which could lead to an overestimation of the polygenic score associations mediated by non-cognitive skills. Sixth, adjusting for heritable covariates such as family SES and general cognitive ability, could introduce collider bias, potentially distorting the relationship between non-cognitive skills and academic achievement^58^. Seventh, our study was conducted within a UK-based twin sample, primarily focused on individuals of White-European ancestry, which, although representative of the UK population for their birth cohort, limits the generalisability of our findings to other populations with different socio-cultural and ancestral backgrounds. Increasingly diverse samples and methodological advances in genetic research, such as multi-ancestry GWASs^59^ and novel GWAS methods^60^, will help bridge this gap in future studies. Furthermore, dizygotic twins may not accurately represent singleton populations. Although recent large‑scale studies^61^ report negligible mean differences in academic achievement between twins and singletons, caution is still warranted when generalising from a twin sample to singleton populations. Finally, academic achievement was measured using teacher ratings rather than standardized test scores. However, prior work in TEDS has demonstrated that these ratings provide reliable estimates of academic performance and correlate strongly with standardized assessments across compulsory education^62^. While we did not consider standardized test-based measures of academic abilities (e.g., numeracy or reading accuracy) in the current study due to their limited utility for our developmental mediation framework, previous work in the TEDS sample has shown that teacher-rated and exam scores are strongly correlated with test-based abilities^63^. Furthermore, our results were highly consistent after we statistically controlled for general cognitive ability (g), measures as a composite of verbal and non-verbal reasoning tests, which are strongly associated with academic outcomes^64,65^.

## Methods

### Participants

Our study involved twins who were part of the Twins Early Development Study (TEDS)^66^. All twins born between 1994 and 1996 in England and Wales, as identified through birth records, were invited via their parents to participate. The invitations were sent to families by the UK Office for National Statistics after screening for infant mortality, and 16,810 families expressed interest in taking part. The initial data collection involved the participation of over 13,000 twin pairs, and after almost three decades, TEDS maintains an active membership of over 10,000 families. TEDS sample remains largely representative of the demographic characteristics of the UK population for their respective generation in terms of ethnicity and socio-economic status^66,67^. The subsample included in our current analyses comprised 5,016 individuals (2,895 DZ sibling pairs for the within-family design) whose families had contributed data on academic achievement, non-cognitive skills, and had available genotype information. The gender distribution within the sample was approximately 51.1% female and 48.9% male. Our examination spanned four waves of data collection: age 7 (Mean age = 7.20), age 9 (Mean age = 9.03), age 12 (Mean age = 11.52), and age 16 (Mean age = 16.31). Mean ages are derived from teacher assessment of academic achievement. Participants with significant medical, genetic, or neurodevelopmental conditions were excluded from our analyses, resulting in a variable sample size ranging from 1293 to 5016 due to the inclusion of different variables. TEDS has received ethical approval from the research ethics committee of Kings College London (references: PNM/09/10–104 and HR/DP-20/21–22060). Consent was obtained before data collection at every wave.

### Measures

Data were collected by means of questionnaires and tests administered to parents, teachers, and the twins themselves by post, telephone, and online, as described in detail in this overview of the TEDS study^67^ and in the TEDS data dictionary (https://www.teds.ac.uk/datadictionary/home.htm).

#### Academic achievement

At ages 7, 9 and 12, academic achievements were provided by the teachers who assessed students’ performance based on the UK National Curriculum guidelines designed by the National Foundation for Educational Research (NFER; http://www.nfer.ac.uk/index.cfm) and the Qualifications and Curriculum Authority (QCA; http://www.qca.org.uk). Consistent with the standardised assessment at Key Stage 1 (KS1), at age 7 academic achievement was measured as a composite of standardised performance in English and mathematics. At age 9 and 12, and consistent with the standardised assessment at KS2 and KS3, achievement was measured as a composite score of performance across three subjects: English, mathematics, and science. To ensure comparability across ages, we conducted additional sensitivity analyses using a composite score including only English and mathematics at ages 7, 9 and 12, as well as analyses examining individual subjects separately (Supplementary Data 7).

Academic achievement at age 16 was measured as the mean grade score for the General Certificate of Secondary Education (GCSE) three subjects (English, mathematics and science). GCSEs are standardized tests taken at age 16, which in the UK for the TEDS sample was the end of compulsory education. The exams are graded on a scale ranging from A* to G, with a U grade assigned for unsuccessful attempts. The grades were coded on a scale from 11 (A*) to 4 (G, the lowest passing grade), and the mean of the grade obtained from the GCSE core subjects was used as our measure of academic achievement at age 16. Data on GCSE exam results were collected from parental and self-reports via questionnaires sent over mail or telephone. Our previous research has shown that teacher ratings and self-reported GCSE grades are valid, reliable, and correlate very strongly with standardized exam scores taken at specific moments in the educational curriculum (Key Stages) obtained from the National Pupil Database^62^.

#### Non-cognitive skills

Our measures of non-cognitive skills included measures of behaviours, attitudes and motivations that have been found to associate with academic outcomes beyond performance in cognitive tests^28,34,39,48^. A full list of included variables and their sources is provided below. More details can be found in the TEDS data dictionary (https://www.teds.ac.uk/datadictionary), which provided detailed description of each measure and information on the items included in each construct. Reliability information for non-cognitive measurements is provided in Supplementary Note 2.

### Education-specific non-cognitive skills

At age 9 data on education-specific non-cognitive skills were collected from twins themselves, their parents and teachers. Measures of academic self-perceived ability^68^, academic interest^68^ and the Classroom Environment Questionnaire (CEQ^69^) were available from all raters. The CEQ included the following subscales rated by parents and twins: (1) CEQ classroom satisfaction scale; (2) CEQ educational opportunities scale; (3) CEQ adventures scales, assessing enjoyment of learning. Ratings on the CEQ classroom satisfaction scale were also provided by the teachers.

At age 12, data on education-specific non-cognitive skills were collected from self-reports. We collected the following measures: academic interest^68^, academic self-perceived ability^68^, the literacy environment questionnaire^70,71^ and the mathematics environment questionnaire^72^. The questionnaires asked several questions related to literacy and mathematics, for example: *‘How much do you like reading?’;* rated on 5-point Likert scale from 1 = *‘Don’t like it at all’* to 5 = *‘Like it very much’*, and *‘How good do you think you are at doing maths in your head?’* rated on 5-point Likert scale from 1 = *‘Not at all good’* to 5 = *‘Very good’*. Items were reverse-coded where necessary so that higher scores indicate greater perceived ability or liking.

At age 16 education-specific non-cognitive skills were assessed via self-reports provided by the twins. The battery of education-specific non-cognitive constructs included the following measures:

Academic self-concept: 10 items of a brief academic self-concept scale (adapted from^73^, e.g., *‘I am good at doing tests’)* rated on a 5-point Likert scale from 5 = *‘Very much like me’* to 1 = *‘Not like me at all’*. Items were reverse coded where necessary with higher scores represent greater academic self-concept.

School engagement^74^: a composite includes 5 subscales: future aspirations and goals; control and relevance of schoolwork; teacher-student relations; peer support for learning; family support for learning. The scale includes items rated on four-point Likert scale from 1 = *‘Strongly disagree’* to 4 = *‘Strongly agree’*, such as: ‘*School is important for achieving my future goals’*, ‘*I feel like I have a say about what happens to me at school’*, ‘*I enjoy talking to the teachers at my school’*, and ‘*When I have problems at school, my family/carer(s) are willing to help me’*).

Grit was measured with 8 items from the Short Grit Scale (GRIT-S)^75^ asking the twins to report on their academic perseverance answering questions (e.g., ‘*Setbacks don’t discourage me*, and *I am a hard worker’)* rated on a 5-point Likert scale from 5 = *‘Very much like’*, to 1 = *‘Not like me at all’*. Items were reverse-coded where necessary with higher scores represent greater grit.

Academic ambition^76^ was assessed with 5 items asking participants to rate statements like the following: ‘*I am ambitious* and *achieving something of lasting importance is the highest goal in life’*, on a 5-point Likert scale from 5 = *‘Very much like’*, to 1 = *‘Not like me at all’*. Items were reverse-coded where necessary with higher scores represent greater academic ambition.

Time spent on mathematics was measured with 3 items asking participants how much time every week they spent in, including ‘*Regular lessons in mathematics at school’, ‘Out-of school-time lessons in mathematics’*, and ‘*Study or homework in mathematics by themselves’*. The items were rated on 5-point Likert scale, from 0 = *‘No time’* to 4 = *‘6 or more hours’*.

Mathematics self-efficacy (OECD Programme for International Student Assessment (PISA)) was assessed with 8 items rated on four-point Likert scale. The scale including questions to measure how confident the student felt about having to conduct different mathematics tasks (e.g., ‘*Calculating how many square metres of tiles you need to cover a floor* and *Understanding graphs presented in newspapers’*).

Mathematics interest (OECD Programme for International Student Assessment (PISA)) was assessed with 3 questions, rated on 4-point Likert scale from 1 = *‘Strongly disagree’* to 4 = *‘Strongly agree’*, asking participants about how their interest in mathematics, such as: ‘*I do mathematics because I enjoy it* and *I am interested in the things I learn in mathematics’*.

Curiosity was assessed with 7 items^77^ rated on 7-point Likert scale, from 7 = *‘Strongly agree’* to 1 = *‘Strongly disagree’*. Students were asked to rate statements including: ‘*When I am actively interested in something, it takes a great deal to interrupt me* and ‘*Everywhere I go, I am looking out for new things or experiences’*.

Attitudes towards school was assessed using the OECD Programme for International Student Assessment (PISA) attitudes to school measure which included 4 items rated on a 4-point Likert scale from 1 = *‘Strongly disagree’*, to 4 = *‘Strongly agree’*, such as: ‘*School has helped give me confidence to make decisions’* and ‘*School has taught me things which could be useful in a job’*.

### Emotional and behavioural functioning

The Strengths and Difficulties Questionnaire (SDQ)^78^ was used to evaluate emotional and behavioural functioning across all ages, consistent with previous work using the same dataset^26^ and aligned with the economics literature^39,54^. Data on emotional and behavioural functioning was collected from parents, teachers, and self-reports of the twins and rated on 3-point Likert scale from 0 = *‘Not true’* to 2 = *‘Very true’*. The SDQ comprises five subscales: conduct problems, peer problems, hyperactivity, emotional difficulties, and prosocial behaviour. Scores from all subscales, except prosocial behaviour, were reversed to ensure higher scores indicate better emotional and behavioural functioning. Parents rated emotional and behavioural functioning at age 7, while assessments at ages 9 and 12 involved inputs from parents, teachers, and the twins. At age 16, twins reported their own emotional and behavioural functioning.

#### Cognitive ability

Cognitive ability at age 7 was evaluated through four tests administered via telephone by trained research assistants. Verbal cognitive ability was measured using two tests adapted from the Wechsler Intelligence Scale for Children (WISC-III)^79^: a 13-item Similarity test and an 18-item Vocabulary test. Nonverbal cognitive ability was evaluated with a 9-item Conceptual Groupings Test^80^ and a 21-item WISC Picture Completion Test^79^. Composite scores for verbal and nonverbal abilities were calculated by taking the average of the standardized scores within each respective domain. Furthermore, a general cognitive ability composite (*g*) was established by averaging the standardized scores obtained from the two verbal and two nonverbal tests.

Cognitive ability at age 9 was evaluated through four booklets sent to TEDS families by mail. Verbal skills were assessed using the initial sections (20 items) of the WISC-III-PI Words and General Knowledge tests^81^. Nonverbal abilities were measured with the Shapes test (CAT3 Figure Classification^82^ and the Puzzle test (CAT3 Figure Analogies)^82^. Composite scores for verbal and nonverbal skills were created by averaging the standardized scores within each area. Additionally, a *g* composite was calculated by averaging the standardized scores from the two verbal and two nonverbal tests.

Cognitive ability at age 12 was evaluated through four tests administered online. Verbal skills were assessed using the complete versions of the verbal tests conducted at age 9: the entire 30 items from the WISC-III-PI Words test^81^ and 30 items from the WISC-III-PI General Knowledge test^81^. Nonverbal abilities were measured with the 24-item Pattern test (derived from Raven’s Standard Progressive Matrices)^83^ and the 30-item Picture Completion test (WISC-III-UK)^79^. Composite scores for verbal and nonverbal abilities were derived by averaging the standardized scores within each respective domain. Furthermore, a *g* composite was computed by averaging the standardized scores from the two verbal and two nonverbal tests.

Cognitive ability at age 16 was assessed through a comprehensive evaluation consisting of one verbal test and one nonverbal test administered online. Verbal proficiency was measured using a modified iteration of the Mill Hill Vocabulary test^84^, while nonverbal abilities were gauged through an adapted version of the Raven’s Standard Progressive Matrices test^83^. A *g* composite was determined by averaging the standardized scores derived from the results of both tests.

#### Family socio-economic status

We used socioeconomic status data collected at the first contact for the models at ages 7, 9, and 12. For the models at age 16, we used the socioeconomic status data available at that age. Specifically, at first contact, TEDS twins’ parents were contacted via postal mail and asked to complete a questionnaire concerning their educational background, employment status, and the age at which the mothers had their first child. A composite measure of socioeconomic status was created by standardizing these three variables and calculating their average. At the age of 16, socioeconomic status information was collected through a web-based questionnaire, and a comprehensive score was generated by averaging the standardized values of five items: household income, the highest educational attainment of both parents, and the employment status of both parents.

#### Genetic data

Two different genotyping platforms were utilized due to genotyping being conducted in two distinct phases, with a 5-year interval between them. Affymetrix GeneChip 6.0 SNP arrays were employed to genotype 3665 individuals, while Illumina HumanOmniExpressExome-8v1.2 arrays were used for genotyping 8122 individuals (including 3607 DZ co-twin samples). Following quality control procedures, genotypes from a total of 10,346 samples (comprising 3320 DZ twin pairs and 7026 unrelated individuals) were deemed acceptable, with 3057 individuals genotyped on Affymetrix and 7289 individuals genotyped on Illumina arrays. The final dataset included 7,363,646 genotyped or well-imputed SNPs. For further details regarding the handling of these samples, refer to the provided source^85^.

#### Polygenic scores

Following the implementation of DNA quality control measures recommended for chip-based genomic data, we developed genome-wide polygenic scores (PGS) utilizing summary statistics obtained from four distinct genome-wide association studies: *educational attainment*^14^, *cognitive ability*^26,86^ and *non-cognitive skills*^26,33^. Each PGS was computed as the weighted sum of an individual’s genotype across all single nucleotide polymorphisms (SNPs), with adjustments made for linkage disequilibrium using LDpred^87^. LDpred is a Bayesian shrinkage method that adjusts for local linkage disequilibrium (the correlation between SNPs) by utilizing data from a reference panel. In our study, we used the target sample (TEDS), limited to unrelated individuals, along with a prior reflecting the genetic architecture of the trait. We constructed polygenic scores (PGS) using an infinitesimal prior, assuming that all SNPs contribute to the genetic architecture of the trait. This approach has been shown to work well for highly polygenic traits like educational attainment (EA). In our regression analyses, both the cognitive (Cog) and non-cognitive (NonCog) PGSs were included in multiple regression models, along with covariates such as age, sex, the first ten principal components of ancestry, and genotyping chip and batch. To address the non-independence of observations, we used the generalized estimating equation (GEE) approach^88^ from the R package.

### Analytic strategies

All scripts are available on our research lab GitHub page (https://github.com/CoDEresearchlab/Noncognitive_mediation)^89^.

#### Data preparation

To control for potential confounding factors, we regressed all continuous variables (academic achievement, non-cognitive skills, and general cognitive ability) on age and sex collected at the same wave and we used the standardised residuals in all our analyses. Supplementary Data 1 presents descriptive statistics for the standardised and age and sex-adjusted variables.

#### Construction of latent factors of non-cognitive skills and general cognitive ability

Confirmatory factor analysis (CFA)^90^ was used to create latent constructs of general cognitive ability and non-cognitive skills across various developmental stages. In alignment with established research on general cognitive ability (*g*) and prior investigations in the TEDS dataset^91^, we modelled a single factor capture general cognitive ability (*g*) at each developmental stage. Each *g* factors was constructed from the weighted loadings of two verbal and two nonverbal tests (see Supplementary Data 23).

CFA was also used to create dimensions reflecting non-cognitive skills. Drawing from previous meta-analytic research on the non-cognitive factors influencing educational outcomes, we adopted a theoretical differentiation between education-specific non-cognitive traits (such as academic related attitudes, motivation, and goals) and broader, more universally applicable measures of emotional and behavioural functioning^24,34^. Consequently, distinct factors were established for a) education-specific non-cognitive skills (NCS) and b) emotional and behavioural functioning, considering the various measures available at each age for each evaluator. Full details of latent factor construction and model fit indices are reported in Supplementary Note 3 and Supplementary Data 24 and 25.

#### Mediation analyses

Following the removal of outliers (scores outside +/− 4 standard deviations), we employed Structural Equation Modelling (SEM) for conducting mediation analyses^92^ using *lavaan* in R^93^. A comprehensive description of the mediation models is provided in Supplementary Note 3. These mediation models enabled us to dissect the effect size of each polygenic score’s prediction on academic achievement throughout development into direct and indirect effects mediated by both latent dimensions of, and individual non-cognitive skill measures. We used Full Information Maximum Likelihood (FIML) to account for missing data. To address the risk of multiple testing, we employed the Benjamini-Hochberg false discovery rate correction (FDR)^94^. Furthermore, to address the non-independence of observations in the sample (i.e., relatedness), we randomly selected one twin from each pair for analysis. Also, we applied bootstrap method^95^ within *lavaan* package, which allows us to assess the variability of our estimates without relying on the assumption of normality in the sampling distribution. We used the default of 1,000 bootstrap samples in *lavaan* to estimate standard errors and confidence intervals for indirect effects, given their typically non-normal sampling distribution.

#### Cross-sectional analyses

We first conducted mediation models for the non-cognitive skills and academic achievement at the same developmental stage across compulsory education, examining the pathway from polygenic scores to non-cognitive skills and academic achievement measured at the same age. For example, we investigated whether the education-specific non-cognitive skills assessed at age 9 mediated the polygenic score effects on academic achievement at the same age. We selected mediators based on data availability during the same collection wave as each academic achievement outcome.

#### Longitudinal mediation analyses

We further applied time-lagged models, using non-cognitive skills measured at earlier ages as mediators for later academic achievement to investigate whether the non-cognitive skills mediated genetic disposition with academic achievement longitudinally. For instance, we conducted mediation models by including education-specific non-cognitive skills assessed at age 9 and academic achievement at ages 12 and 16.

#### Two-mediator mediation analyses

To account for potential confounding effects, we utilized *two-mediator mediation models*^96^ to expand our examination of mediation effects and explore whether the mediating role of non-cognitive skills was primarily influenced by either family socioeconomic status (SES) or general cognitive ability (*g*). Therefore, we reiterated our mediation models by simultaneously considering each non-cognitive skill alongside family SES and the *g* composite.

#### Multi-level mediation analysis: separating between from within-family effects

We further utilized *1-1-1 two-level mediation models*^97^ to disentangle within-family effects from between-family effects. This statistical framework enabled us to assess the indirect impact of a predictor on an outcome while considering clustered mediation data by mean-centring the siblings’ measurements and taking the departure from the mean for each sibling. In our analyses, we organized our data by family, with each family representing a cluster comprising two members (the two dizygotic twins). By employing *1-1-1* multilevel mediation models, we could separate the between-sibling polygenic score effects, as well as mediation effects. Between-family effects estimated from samples of unrelated individuals are typically biased by sibling-invariant family level demographic factors such as genetic nurture and population stratification and passive gene-environment correlation^17,20,98^. Within-family analyses rely on the randomization of allele transmission during meiosis, ensuring that siblings have an equal chance of inheriting any given allele, independent of environmental processes. Consequently, genetic differences between siblings are unaffected by environmental factors making within-family effects robust to major sources of environmental contributions. Our within-family mediation estimates are therefore indicative of how each sibling’s genetic disposition towards education combines with individual-specific socio-emotional processes to lead to differential academic outcomes through evocative/active gene-environment correlation processes. In these analyses, only dizygotic (DZ) twins were included, as our objective was to explore how differences in polygenic scores between siblings predicted variation in academic achievement through disparities in the non-cognitive skills.

All analyses were preregistered prior to data examination on 19 March 2024 (https://osf.io/vmpf7/), and the reported analyses are consistent with the preregistered plan with no deviations.

### Reporting summary

Further information on research design is available in the Nature Portfolio Reporting Summary linked to this article.

## Supplementary information

Supplementary Information

Description of Additional Supplementary Files

Supplementary Data 1-26

Reporting Summary

Transparent Peer Review file

## Data Availability

The data that support the findings of this study are from the Twins Early Development Study (TEDS), held by King’s College London. Data can be made available subject to a data sharing agreement, as detailed at https://www.teds.ac.uk/researchers/teds-data-access-policy.

## Code availability

The code is available at: https://github.com/CoDEresearchlab/Noncognitive_mediation^89^.

## References

[1] Cutler, D. M. & Lleras-Muney, A. Education and Health: Insights from International Comparisons. Working Paper at 10.3386/w17738 (2012).

[2] Murray-Harvey, R. Relationship influences on students’ academic achievement, psychological health and well-being at school. *Educ. Child Psychol.* **27**, 104–115 (2010).

[3] Watts, T. W. Academic achievement and economic attainment: reexamining associations between test scores and long-run earnings. *AERA Open* **6**, 2332858420928985 (2020).

[4] Currie, J. & Thomas, D. Early test scores, socioeconomic status and future outcomes. *Working Paper* at 10.3386/w6943 (1999).

[5] Montez, J. K. & Hayward, M. D. Cumulative childhood adversity, educational attainment, and active life expectancy among U.S. adults. *Demography* **51**, 413–435 (2014). 24281740 10.1007/s13524-013-0261-x PMC4465758

[6] Malanchini, M., Rimfeld, K., Allegrini, A. G., Ritchie, S. J. & Plomin, R. Cognitive ability and education: how behavioural genetic research has advanced our knowledge and understanding of their association. *Neurosci. Biobehav. Rev.* **111**, 229–245 (2020). 31968216 10.1016/j.neubiorev.2020.01.016 PMC8048133

[7] Rimfeld, K. et al. The stability of educational achievement across school years is largely explained by genetic factors. *Npj Sci. Learn.* **3**, 1–10 (2018). 30631477 10.1038/s41539-018-0030-0 PMC6220264

[8] Branigan, A. R., McCallum, K. J. & Freese, J. Variation in the heritability of educational attainment: an international meta-analysis. *Soc. Forces* **92**, 109–140 (2013).

[9] Trzaskowski, M. et al. DNA evidence for strong genome-wide pleiotropy of cognitive and learning abilities. *Behav. Genet.* **43**, 267–273 (2013). 23609157 10.1007/s10519-013-9594-x PMC3690183

[10] Krapohl, E. & Plomin, R. Genetic link between family socioeconomic status and children’s educational achievement estimated from genome-wide SNPs. *Mol. Psychiatry* **21**, 437–443 (2016). 25754083 10.1038/mp.2015.2 PMC4486001

[11] Dudbridge, F. Power and predictive accuracy of polygenic risk scores. *PLOS Genet* **9**, e1003348 (2013). 23555274 10.1371/journal.pgen.1003348 PMC3605113

[12] Belsky, D. W. & Harden, K. P. Phenotypic annotation: using polygenic scores to translate discoveries from genome-wide association studies from the top down. *Curr. Dir. Psychol. Sci.* **28**, 82–90 (2019). 38736689 10.1177/0963721418807729 PMC11086979

[13] Okbay, A. et al. Polygenic prediction of educational attainment within and between families from genome-wide association analyses in 3 million individuals. *Nat. Genet*. 1–13 10.1038/s41588-022-01016-z (2022). 10.1038/s41588-022-01016-z PMC9005349 35361970

[14] Lee, J. J. et al. Gene discovery and polygenic prediction from a genome-wide association study of educational attainment in 1.1 million individuals. *Nat. Genet.* **50**, 1112–1121 (2018). 30038396 10.1038/s41588-018-0147-3 PMC6393768

[15] Allegrini, A. G. et al. Genomic prediction of cognitive traits in childhood and adolescence. *Mol. Psychiatry* **24**, 819–827 (2019). 30971729 10.1038/s41380-019-0394-4 PMC6986352

[16] von Stumm, S. et al. Predicting educational achievement from genomic measures and socioeconomic status. *Dev. Sci.* **23**, e12925 (2020). 31758750 10.1111/desc.12925 PMC7187229

[17] Zhou, Q. et al. Gene-environment correlation: the role of family environment in academic development. *Mol. Psychiatry* **30**, 999–1008 (2025). 39232197 10.1038/s41380-024-02716-0 PMC11835719

[18] Plomin, R., DeFries, J. C. & Loehlin, J. C. Genotype-environment interaction and correlation in the analysis of human behavior. *Psychol. Bull.* **84**, 309–322 (1977). 557211

[19] Austerberry, C. et al. Evocative effects on the early caregiving environment of genetic factors underlying the development of intellectual and academic ability. *Child Dev.* **95**, 2082–2101 (2024). 39081003 10.1111/cdev.14142 PMC11579646

[20] Kong, A. et al. The nature of nurture: effects of parental genotypes. *Science* **359**, 424–428 (2018). 29371463 10.1126/science.aan6877

[21] Koellinger, P. D. & Harden, K. P. Using nature to understand nurture. *Science* **359**, 386–387 (2018). 29371452 10.1126/science.aar6429 PMC7162683

[22] Wertz, J. et al. Using DNA from mothers and children to study parental investment in children’s educational attainment. *Child Dev.* **91**, 1745–1761 (2020). 31657015 10.1111/cdev.13329 PMC7183873

[23] Heckman, J. J. & Rubinstein, Y. The importance of noncognitive skills: lessons from the GED testing program. *Am. Econ. Rev.* **91**, 145–149 (2001).

[24] Schneider, M. & Preckel, F. Variables associated with achievement in higher education: a systematic review of meta-analyses. *Psychol. Bull.* **143**, 565–600 (2017). 28333495 10.1037/bul0000098

[25] Krapohl, E. et al. The high heritability of educational achievement reflects many genetically influenced traits, not just intelligence. *Proc. Natl. Acad. Sci. USA* **111**, 15273–15278 (2014). 25288728 10.1073/pnas.1408777111 PMC4210287

[26] Malanchini, M. et al. Genetic associations between non-cognitive skills and academic achievement over development. *Nat. Hum. Behav.* **8**, 2034–2046 (2024). 39187715 10.1038/s41562-024-01967-9 PMC11493678

[27] Chamorro-Premuzic, T., Harlaar, N., Greven, C. U. & Plomin, R. More than just IQ: a longitudinal examination of self-perceived abilities as predictors of academic performance in a large sample of UK twins. *Intelligence* **38**, 385–392 (2010). 25473141 10.1016/j.intell.2010.05.002 PMC4248677

[28] Malanchini, M., Engelhardt, L. E., Grotzinger, A. D., Harden, K. P. & Tucker-Drob, E. M. Same but different’: associations between multiple aspects of self-regulation, cognition and academic abilities. *J. Pers. Soc. Psychol.* **117**, 1164–1188 (2019). 30550329 10.1037/pspp0000224 PMC6565522

[29] Muenks, K., Wigfield, A., Yang, J. & O’Neal, C. How true is grit? Assessing its relations to high school and college students’ personality characteristics, self-regulation, engagement, and achievement. *J. Educ. Psychol.* **109**, 599–620 (2017).

[30] Tangney, J. P., Baumeister, R. F. & Boone, A. L. High self-control predicts good adjustment, less pathology, better grades, and interpersonal success. *J. Pers.* **72**, 271–324 (2004). 15016066 10.1111/j.0022-3506.2004.00263.x

[31] Belsky, D. W. et al. The genetics of success: how single-nucleotide polymorphisms associated with educational attainment relate to life-course development. *Psychol. Sci.* **27**, 957–972 (2016). 27251486 10.1177/0956797616643070 PMC4946990

[32] Austerberry, C. et al. Early manifestations of intellectual performance: Evidence that genetic effects on later academic test performance are mediated through verbal performance in early childhood. *Child Dev.* **93**, e188–e206 (2022). 34783370 10.1111/cdev.13706 PMC10861934

[33] Demange, P. A. et al. Investigating the genetic architecture of noncognitive skills using GWAS-by-subtraction. *Nat. Genet.* **53**, 35–44 (2021). 33414549 10.1038/s41588-020-00754-2 PMC7116735

[34] Richardson, M., Abraham, C. & Bond, R. Psychological correlates of university students’ academic performance: a systematic review and meta-analysis. *Psychol. Bull.* **138**, 353–387 (2012). 22352812 10.1037/a0026838

[35] Price, T. S. & Jaffee, S. R. Effects of the family environment: gene-environment interaction and passive gene-environment correlation. *Dev. Psychol.* **44**, 305–315 (2008). 18331124 10.1037/0012-1649.44.2.305

[36] Baier, T. & Lang, V. The social stratification of environmental and genetic influences on education: new evidence using a register-based twin sample. *Sociol. Sci.* **6**, 143–171 (2019).

[37] Robinson, M. R. et al. Genetic evidence of assortative mating in humans. *Nat. Hum. Behav.* **1**, 1–13 (2017).

[38] Selzam, S. et al. Comparing within- and between-family polygenic score prediction. *Am. J. Hum. Genet.* **105**, 351–363 (2019). 31303263 10.1016/j.ajhg.2019.06.006 PMC6698881

[39] Conti, G. & Heckman, J. J. Understanding the early origins of the education–health gradient: a framework that can also be applied to analyze gene–environment interactions. *Perspect. Psychol. Sci.* **5**, 585–605 (2010). 21738556 10.1177/1745691610383502 PMC3129786

[40] Tucker-Drob, E. M. & Harden, K. P. A behavioral genetic perspective on noncognitive factors and academic achievement. *Genet. Ethics Educ*. 134–158 (2017).

[41] İnan-Kaya, G. & Rubie-Davies, C. M. Teacher classroom interactions and behaviours: indications of bias. *Learn. Instr.* **78**, 101516 (2022).

[42] Chang, C.-C., Tseng, K.-H. & Lou, S.-J. A comparative analysis of the consistency and difference among teacher-assessment, student self-assessment and peer-assessment in a Web-based portfolio assessment environment for high school students. *Comput. Educ.* **58**, 303–320 (2012).

[43] Flake, J. K. & Petway II, K. T. Methodologies for investigating and interpreting student–teacher rating incongruence in noncognitive assessment. *Educ. Meas. Issues Pract.* **38**, 63–77 (2019).

[44] Marsh, H., Trautwein, U., Ludtke, O., Koller, O. & Baumert, J. Academic self-concept, interest, grades, and standardized test scores: reciprocal effects models of causal ordering. *CHILD Dev.* **76**, 397–416 (2005). 15784090 10.1111/j.1467-8624.2005.00853.x

[45] von Stumm, S., Hell, B. & Chamorro-Premuzic, T. The hungry mind: intellectual curiosity is the third pillar of academic performance. *Perspect. Psychol. Sci.* **6**, 574–588 (2011). 26168378 10.1177/1745691611421204

[46] Heckman, J. J. & Kautz, T. Fostering and measuring skills: interventions that improve character and cognition. Working Paper at 10.3386/w19656 (2013).

[47] Algan, Y., Beasley, E., Vitaro, F. & Tremblay, R. E. The impact of non-cognitive skills training on academic and non-academic trajectories: from childhood to early adulthood. Sciences Po Working Paper hal-03429906 (2014).

[48] Smithers, L. G. et al. A systematic review and meta-analysis of effects of early life non-cognitive skills on academic, psychosocial, cognitive and health outcomes. *Nat. Hum. Behav.* **2**, 867–880 (2018). 30525112 10.1038/s41562-018-0461-x PMC6277013

[49] Plomin, R. Genotype-environment correlation in the era of DNA. *Behav. Genet.* **44**, 629–638 (2014). 25195166 10.1007/s10519-014-9673-7 PMC4234822

[50] Non, A. L. & Cerdeña, J. P. Considerations, caveats, and suggestions for the use of polygenic scores for social and behavioral traits. *Behav. Genet.* **54**, 34–41 (2024). 37801150 10.1007/s10519-023-10162-x PMC10822803

[51] Gutman, L. M. & Schoon, I. *The Impact of Non-Cognitive Skills on Outcomes for Young People. A Literature Review*. *Education Endowment Foundation*: *London, UK*. https://educationendowmentfoundation.org.uk/evidence-summaries/evidence-reviews/essential-life-skills/ (2013).

[52] Morris, T. T., Davey Smith, G., van den Berg, G. & Davies, N. M. Consistency of noncognitive skills and their relation to educational outcomes in a UK cohort. *Transl. Psychiatry* **11**, 1–10 (2021). 34741011 10.1038/s41398-021-01661-8 PMC8571267

[53] Duckworth, A. L. & Yeager, D. S. Measurement matters: assessing personal qualities other than cognitive ability for educational purposes. *Educ. Res.* **44**, 237–251 (2015). 27134288 10.3102/0013189X15584327 PMC4849415

[54] Attanasio, O., Blundell, R., Conti, G. & Mason, G. Inequality in socio-emotional skills: a cross-cohort comparison. *J. Public Econ.* **191**, 104171 (2020). 34720241 10.1016/j.jpubeco.2020.104171 PMC8543077

[55] Pingault, J.-B. et al. Research review: how to interpret associations between polygenic scores, environmental risks, and phenotypes. *J. Child Psychol. Psychiatry* **63**, 1125–1139 (2022). 35347715 10.1111/jcpp.13607 PMC9790749

[56] Allegrini, A. G. et al. Multivariable G-E interplay in the prediction of educational achievement. *PLOS Genet* **16**, e1009153 (2020). 33201880 10.1371/journal.pgen.1009153 PMC7721131

[57] MacKinnon, D. P., Krull, J. L. & Lockwood, C. M. Equivalence of the mediation, confounding and suppression effect. *Prev. Sci. J. Soc. Prev. Res.* **1**, 173–181 (2000). 10.1023/a:1026595011371 PMC2819361 11523746

[58] Akimova, E. T., Breen, R., Brazel, D. M. & Mills, M. C. Gene-environment dependencies lead to collider bias in models with polygenic scores. *Sci. Rep.* **11**, 9457 (2021). 33947934 10.1038/s41598-021-89020-x PMC8097011

[59] Yengo, L. et al. A saturated map of common genetic variants associated with human height. *Nature* **610**, 704–712 (2022). 36224396 10.1038/s41586-022-05275-y PMC9605867

[60] Campos, A. I. et al. Boosting the power of genome-wide association studies within and across ancestries by using polygenic scores. *Nat. Genet*. 1–8 10.1038/s41588-023-01500-0 (2023). 10.1038/s41588-023-01500-0 37723263

[61] Christensen, K. et al. Comparison of academic performance of twins and singletons in adolescence: follow-up study. *BMJ* **333**, 1095 (2006). 17012267 10.1136/bmj.38959.650903.7C PMC1661694

[62] Rimfeld, K. et al. Teacher assessments during compulsory education are as reliable, stable and heritable as standardized test scores. *J. Child Psychol. Psychiatry* **60**, 1278–1288 (2019). 31079420 10.1111/jcpp.13070 PMC6848749

[63] Malanchini, M. et al. Genetic factors underlie the association between anxiety, attitudes and performance in mathematics. *Transl. Psychiatry* **10**, 1–11 (2020). 32066693 10.1038/s41398-020-0711-3 PMC7026074

[64] Ritchie, S. J., Bates, T. C. & Plomin, R. Does learning to read improve intelligence? A longitudinal multivariate analysis in identical twins from age 7 to 16. *Child Dev.* **86**, 23–36 (2015). 25056688 10.1111/cdev.12272 PMC4354297

[65] Deary, I. J., Strand, S., Smith, P. & Fernandes, C. Intelligence and educational achievement. *Intelligence* **35**, 13–21 (2007).

[66] Lockhart, C. et al. Twins Early Development Study (TEDS): a genetically sensitive investigation of mental health outcomes in the mid-twenties. *JCPP Adv.* **3**, e12154 (2023). 37753150 10.1002/jcv2.12154 PMC10519737

[67] Rimfeld, K. et al. Twins early development study: a genetically sensitive investigation into behavioral and cognitive development from infancy to emerging adulthood. *Twin Res. Hum. Genet.* **22**, 508–513 (2019). 31544730 10.1017/thg.2019.56 PMC7056571

[68] Spinath, B., Spinath, F. M., Harlaar, N. & Plomin, R. Predicting school achievement from general cognitive ability, self-perceived ability, and intrinsic value. *Intelligence* **34**, 363–374 (2006).

[69] Walker, S. O. & Plomin, R. Nature, nurture, and perceptions of the classroom environment as they relate to teacher-assessed academic achievement: a twin study of nine-year-olds. *Educ. Psychol.* **26**, 541–561 (2006).

[70] The Nation’s Report Card, Grade 4, Student Background Questionnaire on Reading (NAEP, 2005).

[71] The Nation’s Report Card: Reading 2005. https://nces.ed.gov/pubsearch/pubsinfo.asp?pubid=2006451 (2005).

[72] The Nation’s Report Card, Grade 4, Student Background Questionnaire on Mathematics (NAEP, 2005).

[73] Burden, R. Assessing children’s perceptions of themselves as learners and problem-solvers: the construction of the myself-as-learner scale (MALS). *Sch. Psychol. Int.* **19**, 291–305 (1998).

[74] Appleton, J. J., Christenson, S. L., Kim, D. & Reschly, A. L. Measuring cognitive and psychological engagement: validation of the student engagement instrument. *J. Sch. Psychol.* **44**, 427–445 (2006).

[75] Duckworth, A. L. & Quinn, P. D. Development and validation of the short grit scale (Grit–S). *J. Pers. Assess.* **91**, 166–174 (2009). 19205937 10.1080/00223890802634290

[76] Duckworth, A. L., Peterson, C., Matthews, M. D. & Kelly, D. R. Grit: perseverance and passion for long-term goals. *J. Pers. Soc. Psychol.* **92**, 1087–1101 (2007). 17547490 10.1037/0022-3514.92.6.1087

[77] Kashdan, T. B., Rose, P. & Fincham, F. D. Curiosity and exploration: facilitating positive subjective experiences and personal growth opportunities. *J. Pers. Assess.* **82**, 291–305 (2004). 15151805 10.1207/s15327752jpa8203_05

[78] Goodman, R. The strengths and difficulties questionnaire: a research note. *J. Child Psychol. Psychiatry* **38**, 581–586 (1997). 9255702 10.1111/j.1469-7610.1997.tb01545.x

[79] Wechsler, D. *Wechsler Intelligence Scale for Children, Manual*. v, 113 (The Psychological Corp., Oxford, England, 1949).

[80] McCarthy, D. *Manual for the McCarthy Scales of Children’s Abilities* (The Psychological Corporation, New York, New York, 1972).

[81] Kaplan, E. *Wechsler Intelligence Scale for Children as a Process Instrument: -WISC-III PI* (Psychological Corporation, 1999).

[82] Smith, P., Fernandes, C. & Strand, S. Cognitive abilities test 3 (CAT3). *Windsor NferNELSON* (2001).

[83] Raven, J. C., Court, J. H. & Raven, J. *Manual for Raven’s Progressive Matrices and Vocabulary Scales*. *Section 3, Standard Progressive Matrices* (Oxford Psychologists Press, Oxford, 1996).

[84] Raven, J. C., Raven, J. E. & Court, J. H. *Mill Hill Vocabulary Scale* (Psychological Corporation, 1989).

[85] Selzam, S. et al. Evidence for gene-environment correlation in child feeding: links between common genetic variation for BMI in children and parental feeding practices. *PLOS Genet* **14**, e1007757 (2018). 30457987 10.1371/journal.pgen.1007757 PMC6245504

[86] Savage, J. E. et al. Genome-wide association meta-analysis in 269,867 individuals identifies new genetic and functional links to intelligence. *Nat. Genet.* **50**, 912–919 (2018). 29942086 10.1038/s41588-018-0152-6 PMC6411041

[87] Vilhjálmsson, B. J. et al. Modeling linkage disequilibrium increases accuracy of polygenic risk scores. *Am. J. Hum. Genet.* **97**, 576–592 (2015). 26430803 10.1016/j.ajhg.2015.09.001 PMC4596916

[88] Vanegas, L. H., Rondón, L. M. & Paula, G. A. Generalized estimating equations using the new R package glmtoolbox. *R. J.* **15**, 105–133 (2023).

[89] Quan Zhou. CoDEresearchlab/Noncognitive_mediation: Code for [Non-cognitive skills mediate genetic effects on academic achievement across development]. *Zenodo*10.5281/ZENODO.19330502 (2026).

[90] Brown, T. A. *Confirmatory Factor Analysis for Applied Research* 2nd edn (Guilford Publications, 2015).

[91] Stumm, S. von, Malanchini, M. & Fisher, H. L. The developmental interplay between the p-factor of psychopathology and the g-factor of intelligence from age 7 through 16 years. *Dev. Psychopathol.* **36**, 1479–1488 (2024). 10.1017/S095457942300069X 37403365

[92] Gunzler, D., Chen, T., Wu, P. & Zhang, H. Introduction to mediation analysis with structural equation modeling. *Shanghai Arch. Psychiatry* **25**, 390–394 (2013). 24991183 10.3969/j.issn.1002-0829.2013.06.009 PMC4054581

[93] Rosseel, Y. lavaan: an R package for structural equation modeling. *J. Stat. Softw.* **48**, 1–36 (2012).

[94] Benjamini, Y. & Hochberg, Y. On the adaptive control of the false discovery rate in multiple testing with independent statistics. *J. Educ. Behav. Stat.* **25**, 60–83 (2000).

[95] DiCiccio, T. J. & Efron, B. Bootstrap confidence intervals. *Stat. Sci.* **11**, 189–228 (1996).

[96] VanderWeele, T. J. & Vansteelandt, S. Mediation analysis with multiple mediators. *Epidemiol. Methods* **2**, 95–115 (2014). 25580377 10.1515/em-2012-0010 PMC4287269

[97] Tofighi, D., West, S. G. & MacKinnon, D. P. Multilevel mediation analysis: the effects of omitted variables in the 1–1–1 model. *Br. J. Math. Stat. Psychol.* **66**, 290–307 (2013). 22594884 10.1111/j.2044-8317.2012.02051.x PMC4814716

[98] Morris, T. T., Davies, N. M., Hemani, G. & Smith, G. D. Population phenomena inflate genetic associations of complex social traits. *Sci. Adv.* **6**, eaay0328 (2020). 32426451 10.1126/sciadv.aay0328 PMC7159920

