# Supplementary material for: Non-cognitive skills mediate education-related polygenic score associations with academic achievement across development
Source: Nat Commun. 2026 May 8;17:5133. doi: 10.1038/s41467-026-72838-2 (PMC13249983; doi:10.1038/s41467-026-72838-2)
Supplement: Supplementary file 1 — Supplementary Information [file 41467_2026_72838_MOESM1_ESM.pdf]

Non-cognitive skills mediate education-related polygenic score associations with academic achievement across development

Quan Zhou[1], Wangjingyi Liao[1], Andrea G. Allegrini[2,3], Kaili Rimfeld[3,4], Jasmin Wertz[5], Tim T. Morris[6], Laurel Raffington[7], Robert Plomin[3], Margherita Malanchini[1,3]

[1] Centre for Brain and Behaviour, School of Biological and Behavioural Sciences, Queen Mary University of London, London, UK
[2] Division of Psychology and Language Sciences, University College London, London, UK
[3] Social, Genetic & Developmental Psychiatry Centre, Institute of Psychiatry, Psychology & Neuroscience, King's College London, London, UK
[4] Department of Psychology, Royal Holloway University of London, London, UK
[5] School of Philosophy, Psychology and Language Sciences, The University of Edinburgh, Edinburgh, UK
[6] Centre for Longitudinal Studies, Social Research Institute, University College London, London, UK
[7] Max Planck Research Group Biosocial – Biology, Social Disparities, and Development; Max Planck Center for Human Development, Berlin, Germany

Table of Contents

Supplementary Notes ____ *3*

Supplementary Note 1: Descriptive statistics. ____ 3

Supplementary Note 2: Description of mediation models. ____ 4

Supplementary Figures ____ *7*

Supplementary Figure 1: Correlation matrix between PGS and academic achievement. ____ 7

Supplementary Figure 2: Correlations between latent factors of non-cognitive skills, academic achievement and education and cognition-associated polygenic scores. ____ 8

Supplementary Figure 3: Comparison of indirect effects of educational attainment prediction between g-uncorrected/ g-corrected noncognitive skills using two-mediators model. ____ 9

Supplementary Figure 4: Comparison of indirect effects of cognitive prediction between g-uncorrected/ g-corrected noncognitive skills using two-mediators model. ____ 10

Supplementary Figure 5: Comparison of indirect effects of noncognitive prediction between g-uncorrected/ g-corrected noncognitive skills using two-mediators model. ____ 11

Supplementary Figure 6: Comparison of indirect effects of educational attainment prediction between SES-uncorrected/ SES-corrected noncognitive skills using two-mediators model. ____ 12

Supplementary Figure 7: Comparison of indirect effects of cognitive prediction between SES-uncorrected/ SES-corrected noncognitive skills using two-mediators model. ____ 13

Supplementary Figure 8: Comparison of indirect effects of noncognitive prediction between SES-uncorrected/ SES-corrected noncognitive skills using two-mediators model. ____ 14

Supplementary Notes

Supplementary Note 1: Descriptive statistics.

Descriptive statistics for the variables used in this study are presented in **Supplementary 1**. These include age- and sex-adjusted standardised scores for academic achievement, non-cognitive skills (both individual indicators and latent factors), cognitive ability, and polygenic scores, collected from different raters across multiple developmental stages. Only participants with available genotype data were included in the final analytic sample (*N* range = 1,293–5,016). All variables were approximately normally distributed.

Supplementary Note 2: Information on the reliability of the non-cognitive measures.

Strengths and Difficulties Questionnaire (SDQ): The Cronbach's α for the SDQ has been reported to be .73 on average, with a mean cross-informant correlation = .34, and mean test–retest stability = .62 after 4–6 months[1].

School Engagement (Student Engagement Instrument; SEI: The Cronbach's α values of SEI ranging from .72 to .88 across subscales in the original validation study[2].

Short Grit Scale (GRIT–S)
The Short Grit Scale[3] demonstrates internal consistency values typically between .73 and .83 across validation samples.

Curiosity and Exploration Inventory (CEI)
The Curiosity and Exploration Inventory[4] shows Cronbach's α values ranging approximately from .58 to .80 depending on the subscale (Exploration, Absorption) and sample.

PISA-derived indices (Mathematics self-efficacy, Mathematics interest, and Attitudes toward school)
According to OECD PISA Technical Reports, internal consistency reliabilities for these indices commonly range from α ≈ .70 to .90 across countries and assessment cycles[5].

Other TEDS-adapted measures:
Other TEDS-adapted scales—including Classroom Environment Questionnaire, Academic Self-Perceived Ability, Academic Interest, Academic Self-Concept, Academic Ambition, and the Literacy and Mathematics Environment questionnaires—were adapted from validated educational instruments. Item-level responses for these scales are not provided in the TEDS data release, precluding internal reliability estimation in the present analyses.

Supplementary Note 3: Description of mediation models.

The SEM for this mediation model for the i th subject ($1 \leq i \leq n$) is given by:

$$z_i = \beta_0 + \beta_{xz} x_i + \varepsilon_{zi},$$
$$y_i = \gamma_0 + \gamma_{zy} z_i + \gamma_{xy} x_i + \varepsilon_{yi}$$

It is posited that the error terms (ϵz_i, ϵy_i) are uncorrelated, a critical presumption for causal inference when conducting mediation analysis. The assumption of multivariate normality for the error terms is also made, as it is an essential precondition for defining direct, indirect, and total effects. It should be highlighted that the two structural equations are interconnected, and the inference drawn from them is concurrent, rather than from two separate standard regression equations. More information can be found here [6].

Mediation analyses

We conducted mediation analyses (Baron & Kenny, 1986; Preacher & Kelley, 2011) using the lavaan package for R to examine the direct and indirect effects of the prediction from genetic predisposition (quantified as the PGSs of educational attainment, cognitive and noncognitive skills) and manifestation of variation in academic achievement (Figure 1).

The mediation model estimates the indirect effect of the predictor (X) on the outcome (Y) via a mediator, i.e., an intervening variable (mediator; M; in this project, the single-timepoint or developmental environmental composite) by regressing M on X and regressing Y on both X and M using two separate equations:

$$1)\ M_i = d_{M.X} + aX_i + e_{M.Xi}$$

Where Mi is the mediator for individual I; dM.X is the intercept for the mediator (M); aXi is the slope of M regressed on the predictor (X) and eM.Xi is the measurement error for individual i.

$$2)\ Y_i = d_{Y.MX} + bM_i + c'X_i + e_{Y.MXi}$$

Where Yi is the outcome for individual I; dY.MX is the intercept for the outcome (Y); bMi is the slope of the outcome (Y) regressed on the mediator (M) controlling for the predictor (X); c'Xi is the slope of the outcome (Y) regressed on the predictor (X) controlling for the mediator (M) and eY.MX is the measurement error for individual i.

The indirect effect of the predictor on the outcome (i.e., the mediation effect) is defined by a^b^, with the sample estimate signified by the circumflex ("^").

When $a^{\wedge} \times b^{\wedge} = c^{\wedge} - c^{\wedge\prime}$, then $c^{\wedge} = a^{\wedge} \times b^{\wedge} + c^{\wedge\prime}$. Implementing SEM allows for, a^ and b^ can be derived simultaneously and for testing more complex models with latent class predictor, outcomes, and mediators.

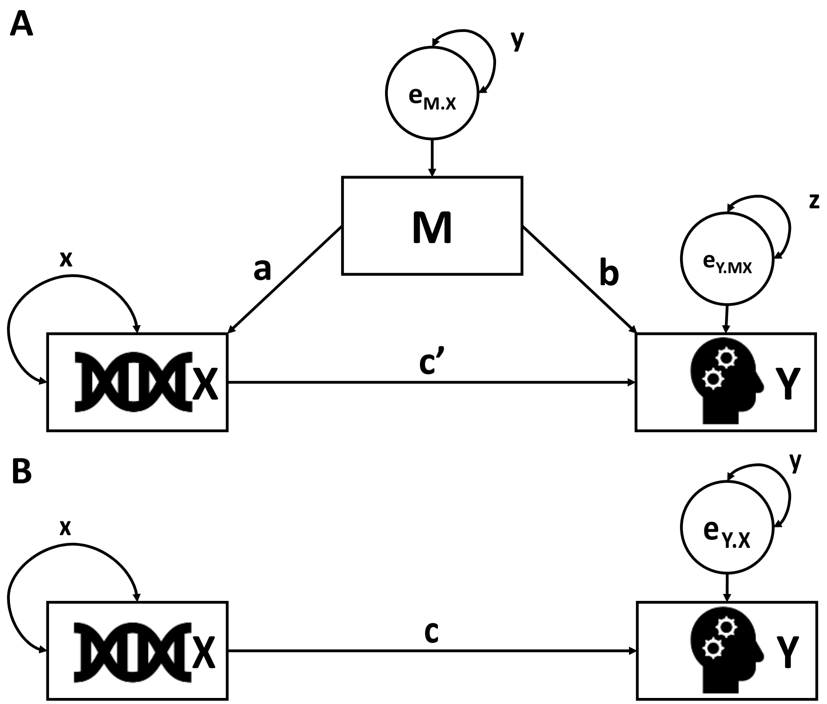

Note: Mediation model of the DNA (X) of cognitive/educational outcomes (Y) that is (panel A) versus is not (panel B) mediated by M, which denotes the noncognitive skills, either single-timepoint or developmental using time-lagged data. Circles indicate residuals. Parameters a, b and c represent regression weights. Parameters x, y and z represent variance parameters.

Supplementary Figures

Supplementary Figure 1: Correlation matrix between PGS and academic achievement.

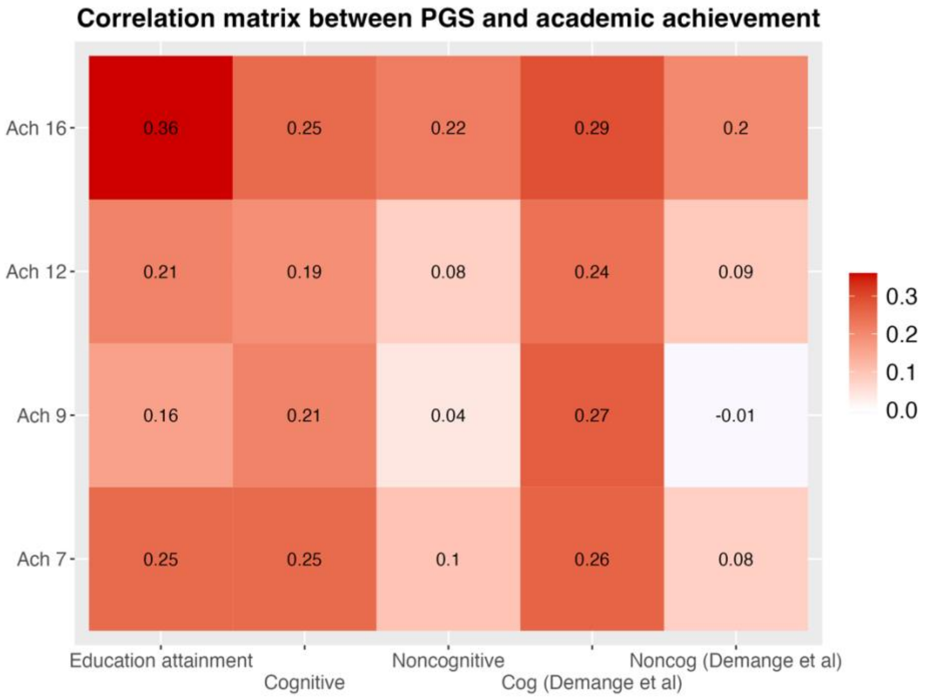

Note: Ach 7: A composite mean score of academic performance at age 7; Ach 9: A composite mean score of academic performance at age 9; Ach 12: A composite mean score of academic performance at age 12; Ach 16: Mean of the grade obtained across the GCSE subjects (English, Maths, and Science); EA: Educational attainment: Educational attainment polygenic score; Cognitive: Cognitive polygenic score; Noncognitive: Non-cognitive polygenic score; Cog (Demange et al): Cognitive polygenic score derived from previous work by Demange et al., 2021; Noncog (Demange et al): Non-cognitive polygenic score derived from previous work by Demange et al., 2021.

Supplementary Figure 2: Correlations between latent factors of non-cognitive skills, academic achievement and education and cognition-associated polygenic scores.

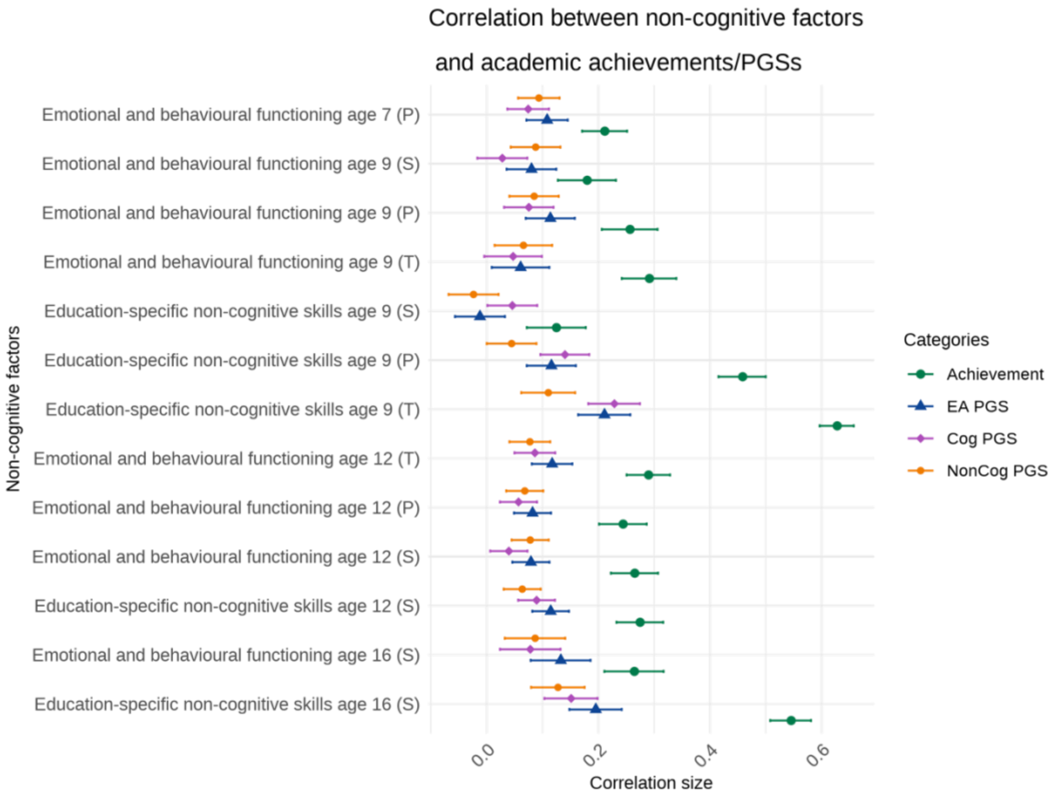

Note: Data on non-cognitive skills was collected from different raters: (P) = parent-reported, (T) = teacher-reported and (S) = Self-reported. Academic achievement was measured contemporaneously with each non-cognitive factor. EA PGS[7] = educational attainment polygenic score. Cog PGS[8] = cognitive skills polygenic score. NonCog PGS[8] = non-cognitive skills polygenic score. Each dot indicates the size of the correlation coefficient, and error bars indicate 95% confidence intervals. Sample size for each correlation analysis is provided in Supplementary Data 2.

Supplementary Figure 3: Comparison of indirect effects of educational attainment PGS prediction between g-uncorrected/ g-corrected noncognitive skills using two-mediators model.

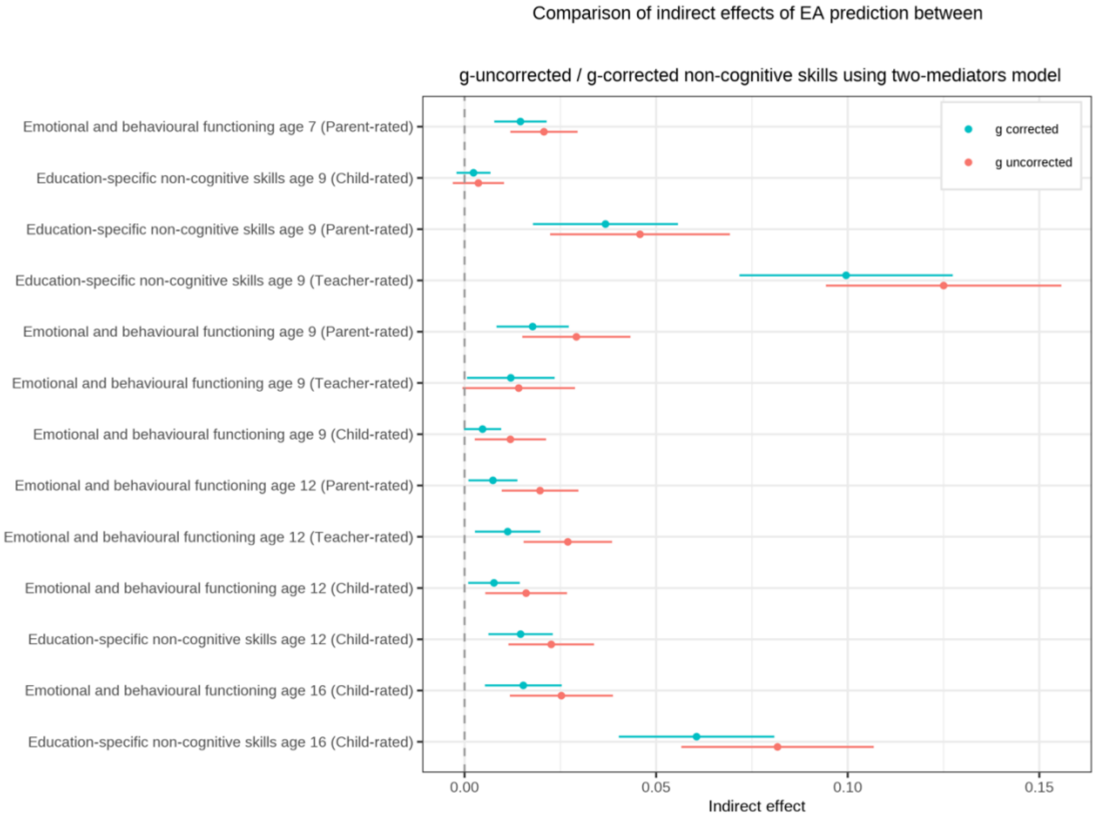

Supplementary Figure 4: Comparison of indirect effects of cognitive PGS prediction between g-uncorrected/ g-corrected noncognitive skills using two-mediators model.

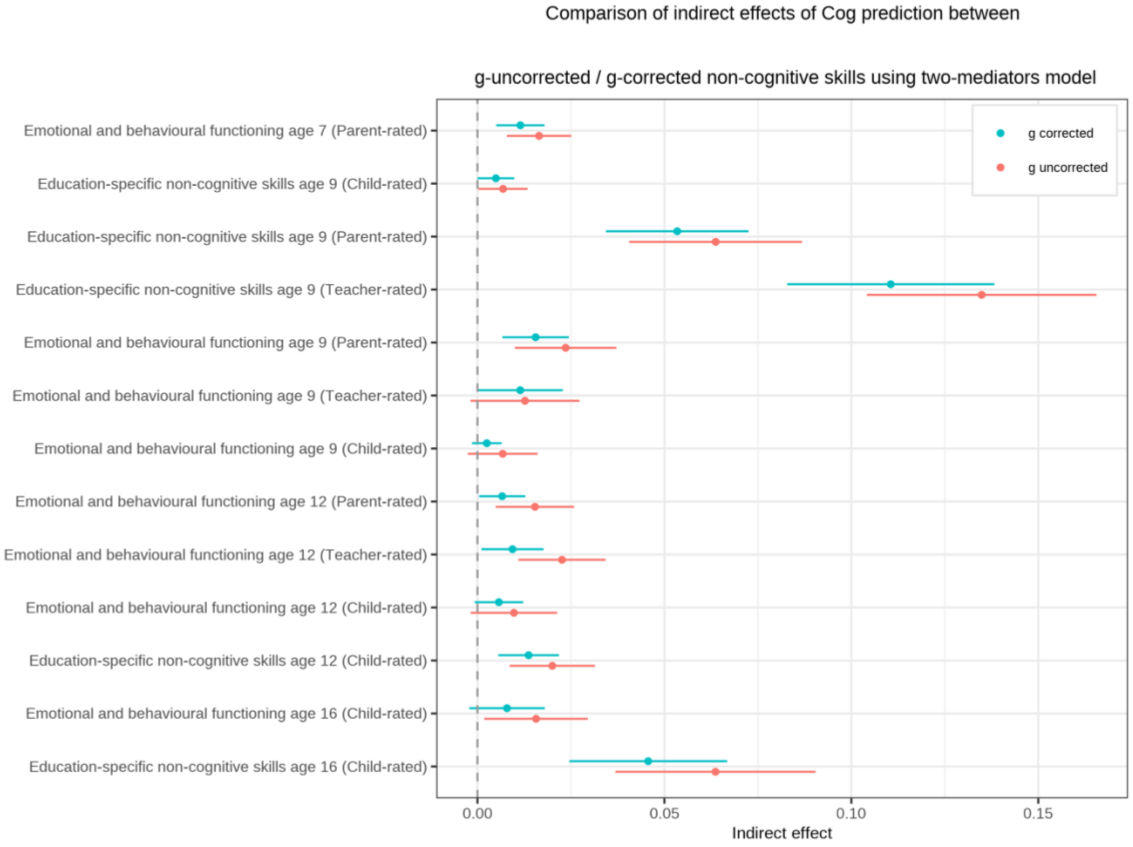

Supplementary Figure 5: Comparison of indirect effects of noncognitive PGS prediction between g-uncorrected/ g-corrected noncognitive skills using two-mediators model.

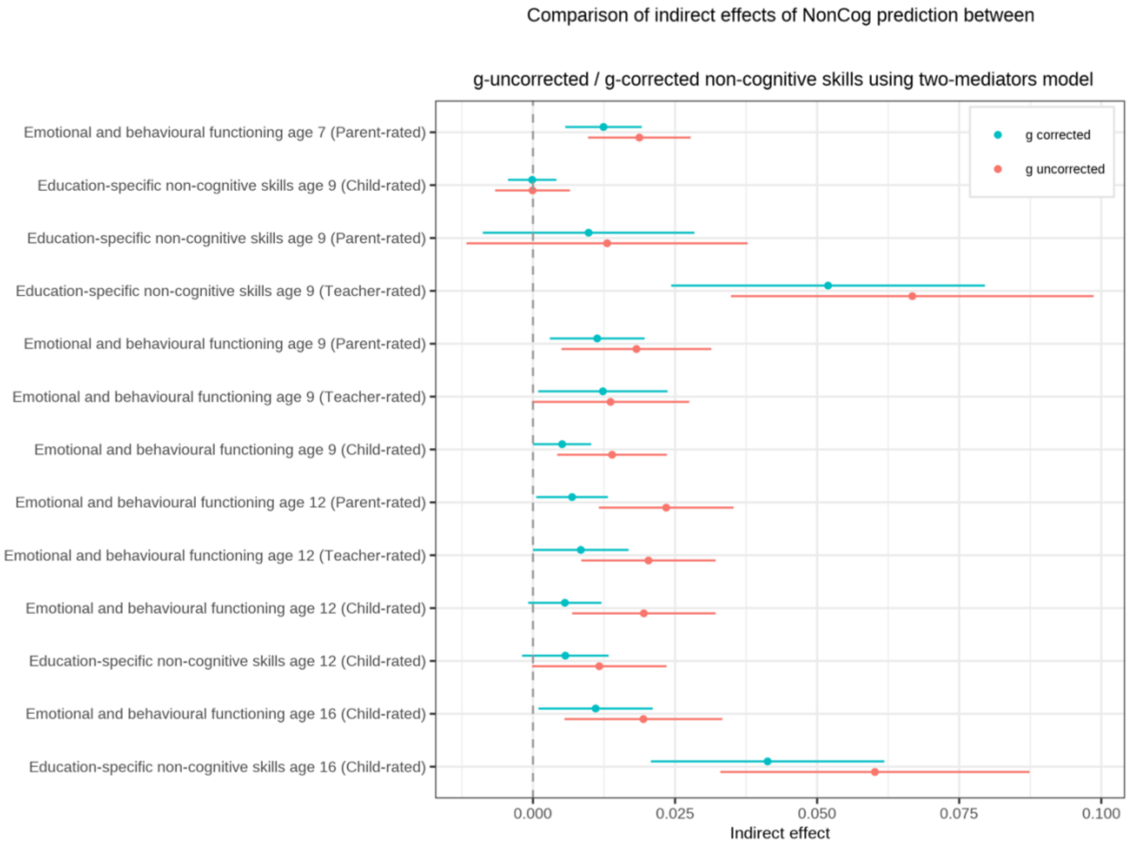

Supplementary Figure 6: Comparison of indirect effects of educational attainment PGS prediction between SES-uncorrected/ SES-corrected noncognitive skills using two-mediators model.

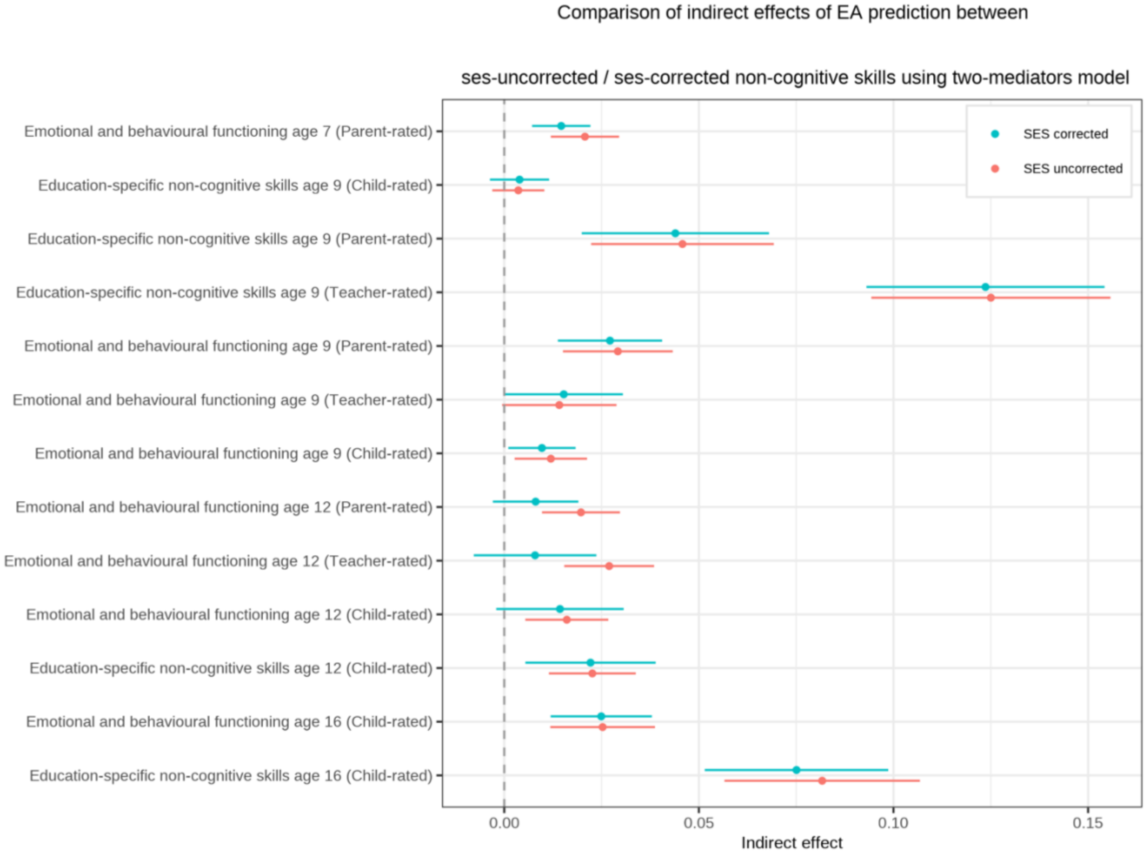

Supplementary Figure 7: Comparison of indirect effects of cognitive PGS prediction between SES-uncorrected/ SES-corrected noncognitive skills using two-mediators model.

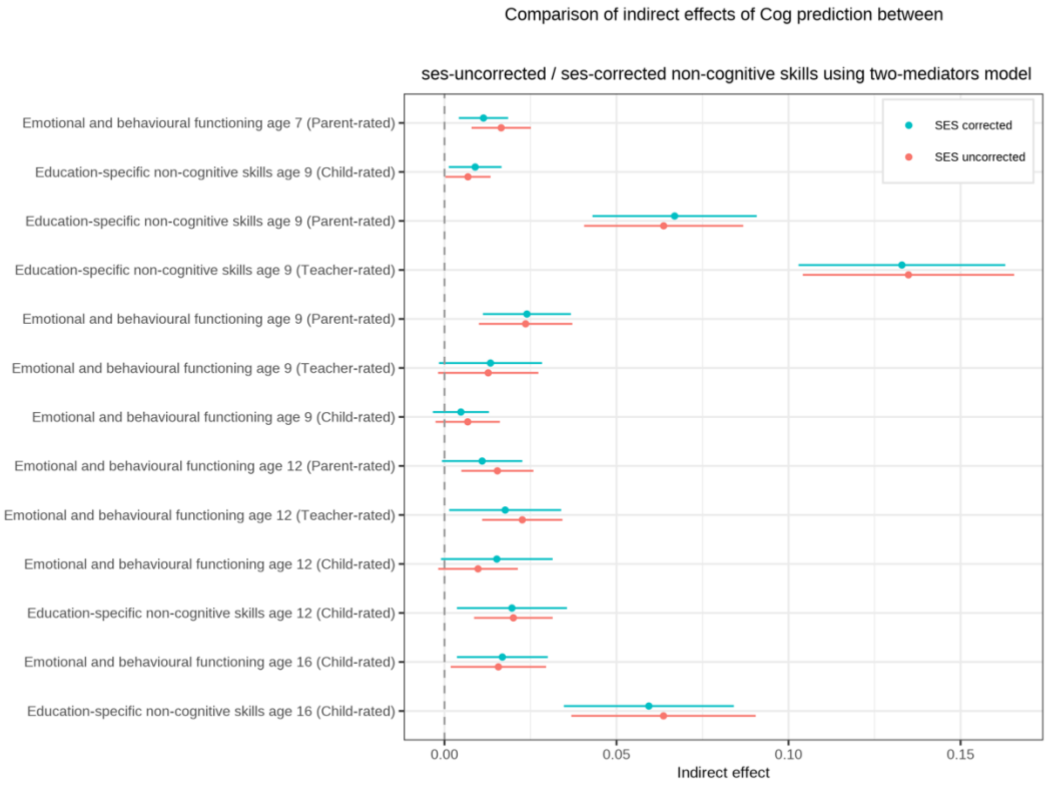

Supplementary Figure 8: Comparison of indirect effects of noncognitive PGS prediction between SES-uncorrected/ SES-corrected noncognitive skills using two-mediators model.

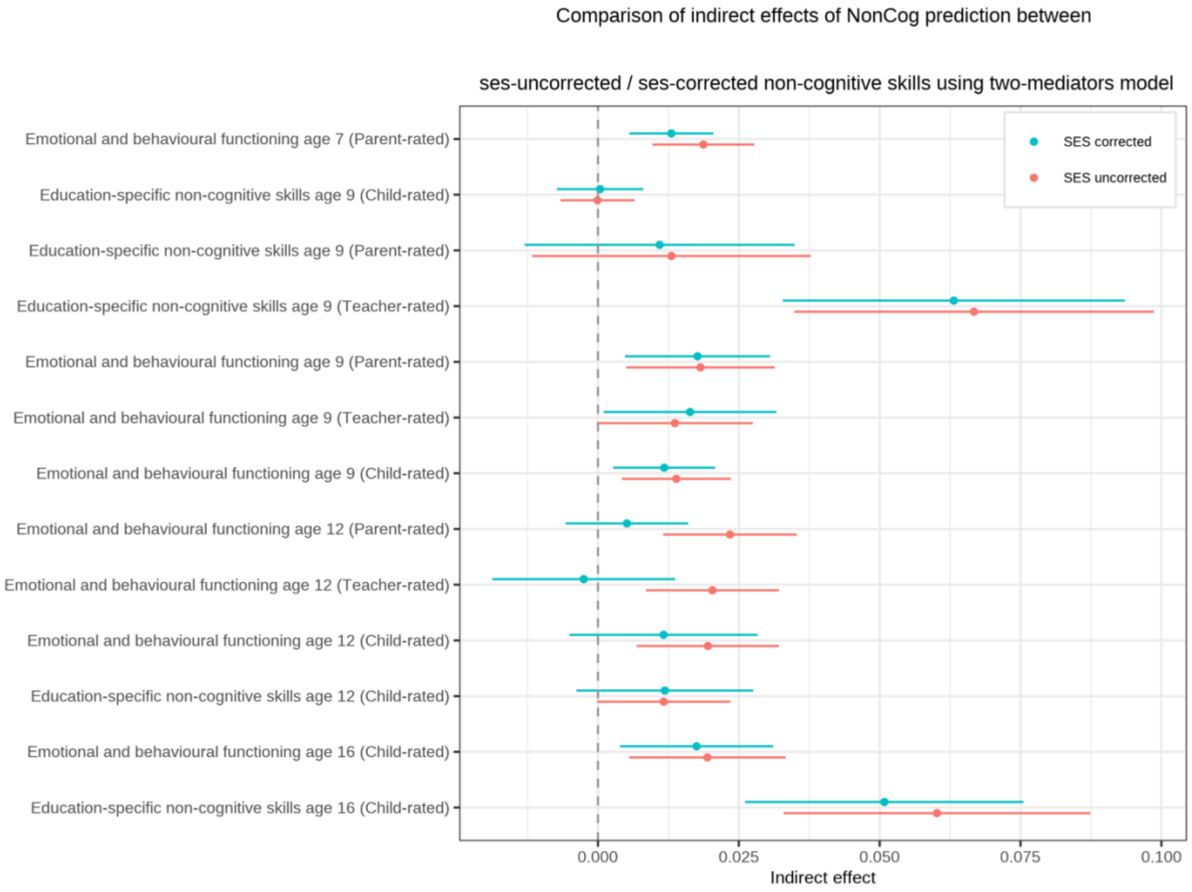

References

1. Goodman, R. Psychometric properties of the strengths and difficulties questionnaire. *J. Am. Acad. Child Adolesc. Psychiatry* **40**, 1337–1345 (2001).
2. Appleton, J. J., Christenson, S. L., Kim, D. & Reschly, A. L. Measuring cognitive and psychological engagement: Validation of the Student Engagement Instrument. *J. Sch. Psychol.* **44**, 427–445 (2006).
3. Duckworth, A. L. & Quinn, P. D. Development and Validation of the Short Grit Scale (Grit–S). *J. Pers. Assess.* **91**, 166–174 (2009).
4. Kashdan, T. B., Rose, P. & Fincham, F. D. Curiosity and Exploration: Facilitating Positive Subjective Experiences and Personal Growth Opportunities. *J. Pers. Assess.* **82**, 291–305 (2004).
5. Organisation for Economic Co-operation and Development (OECD). *PISA 2012 Technical Report*. https://www.oecd.org/pisa/pisaproducts/pisa2012technicalreport.htm (2014).
6. Gunzler, D., Chen, T., Wu, P. & Zhang, H. Introduction to mediation analysis with structural equation modeling. *Shanghai Arch. Psychiatry* **25**, 390–394 (2013).
7. Lee, J. J. *et al.* Gene discovery and polygenic prediction from a genome-wide association study of educational attainment in 1.1 million individuals. *Nat. Genet.* **50**, 1112–1121 (2018).
8. Malanchini, M. *et al.* Genetic associations between non-cognitive skills and academic achievement over development. *Nat. Hum. Behav.* **8**, 2034–2046 (2024).
