# Supplementary material for: Non-cognitive skills mediate education-related polygenic score associations with academic achievement across development
Source: Nat Commun. 2026 May 8;17:5133. doi: 10.1038/s41467-026-72838-2 (PMC13249983; doi:10.1038/s41467-026-72838-2)
Supplement: Supplementary file 2 — Description of Additional Supplementary Files [file 41467_2026_72838_MOESM2_ESM.pdf]

- Supplementary Data 1. Descriptive statistics
- Supplementary Data 2a. Correlation between academic achievement and PGS (Age 7, 9, 12 and 16)
- Supplementary Data 2b. Correlation between noncognitive skills (broader factors) and academic achievements and PGSs
- Supplementary Data 3. Correlation between noncognitive skills (individual measures) and academic achievements and PGSs
- Supplementary Data 4. Mediation model estimates (Noncognitive skill factors) for the prediction from educational attainment PGS on overall academic achievement over development
- Supplementary Data 5. Mediation model estimates (Noncognitive skill factors) for the prediction from Cognitive PGS on overall academic achievement over development
- Supplementary Data 6. Mediation model estimates (Noncognitive skill factors) for the prediction from Noncognitive PGS on overall academic achievement over development
- Supplementary Data 7. Mediation model estimates (full Non-cognitive measures) for the prediction from Educational attainment, Cognitive and Noncognitive PGS on academic achievements over development
- Supplementary Data 8. Mediation model estimates (individual Noncognitive skill measures) for the prediction from EA PGS on overall academic achievement over development
- Supplementary Data 9. Mediation model estimates (individual Noncognitive skill measures) for the prediction from Cognitive PGS on overall academic achievement over development
- Supplementary Data 10. Mediation model estimates (individual Noncognitive skill measures) for the prediction from Noncognitive PGS on overall academic achievement over development
- Supplementary Data 11. Longitudinal (time-lagged) mediation model estimates (education-specific NCS at age 9 and age 12) for the prediction from PGS (EA, Cog and NonCog) on overall academic achievement over development
- Supplementary Data 12. Longitudinal (time-lagged) mediation model estimates (individual education-specific NCS measures at age 9 and age 12) for the prediction from PGS (EA, Cog and NonCog) on overall academic achievement over development
- Supplementary Data 13. Family-level ICCs for noncognitive skill factors
- Supplementary Data 14. Between- and within-family mediation model estimates (noncognitive skill factors) for the prediction from educational attainment PGS on overall academic achievement over development
- Supplementary Data 15. Between- and within-family mediation model estimates (noncognitive skill factors) for the prediction from cognitive PGS on overall academic achievement over development
- Supplementary Data 16. Between- and within-family mediation model estimates (noncognitive skill factors) for the prediction from noncognitive PGS on overall academic achievement over development
- Supplementary Data 17. Between- and within-family mediation model estimates (individual noncognitive skill measures) for the prediction from educational attainment, cognitive and noncognitive PGS on overall academic achievement over development

- Supplementary Data 18. Time-lagged data for between- and within-family mediation model estimates (noncognitive skill factors) for the prediction from educational attainment PGS on overall academic achievement over development
- Supplementary Data 19. Time-lagged data for between- and within-family mediation model estimates (noncognitive skill factors) for the prediction from cognitive PGS on overall academic achievement over development
- Supplementary Data 20. Time-lagged data for between- and within-family mediation model estimates (noncognitive skill factors) for the prediction from noncognitive PGS on overall academic achievement over development
- Supplementary Data 21. Time-lagged data for between- and within-family mediation model estimates (individual education-specific noncognitive skills) for the prediction from educational attainment, cognitive and noncognitive PGS on overall academic achievement over development
- Supplementary Data 22. Comparison of indirect effects of educational attainment, cognitive and noncognitive PGS prediction between uncorrected for general cognitive ability (g) and corrected for g
- Supplementary Data 23. Comparison of indirect effects of educational attainment, cognitive and noncognitive PGS prediction between uncorrected for family socioeconomic status (SES) and corrected for family SES
- Supplementary Data 24. Structure and model fit indices for the latent factors of general cognitive ability over development (a) Age 7; (b) Age 9; (c) Age 12
- Supplementary Data 25. Structure and model fit indices for the latent factors of education-specific noncognitive skills over development (a) Age 9; (b) Age 12; (c) Age 16
- Supplementary Data 26. Structure and model fit indices for the latent factors of domain-general self-regulation skills over development (a) Age 7; (b) Age 9; (c) Age 12; (d) Age 16
