# Supplementary material for: Non-cognitive skills mediate education-related polygenic score associations with academic achievement across development
Source: Nat Commun. 2026 May 8;17:5133. doi: 10.1038/s41467-026-72838-2 (PMC13249983; doi:10.1038/s41467-026-72838-2)
Supplement: Supplementary file 4 — Reporting Summary [file 41467_2026_72838_MOESM4_ESM.pdf]

nature portfolio

Corresponding author(s): Quan Zhou, Margherita Malanchini

Last updated by author(s): 28/03/26

Reporting Summary

Nature Portfolio wishes to improve the reproducibility of the work that we publish. This form provides structure for consistency and transparency in reporting. For further information on Nature Portfolio policies, see our Editorial Policies and the Editorial Policy Checklist.

Statistics

For all statistical analyses, confirm that the following items are present in the figure legend, table legend, main text, or Methods section.

n/a	Confirmed	
☐	☒	The exact sample size (*n*) for each experimental group/condition, given as a discrete number and unit of measurement
☐	☒	A statement on whether measurements were taken from distinct samples or whether the same sample was measured repeatedly
☐	☒	The statistical test(s) used AND whether they are one- or two-sided<br>*Only common tests should be described solely by name; describe more complex techniques in the Methods section.*
☐	☒	A description of all covariates tested
☐	☒	A description of any assumptions or corrections, such as tests of normality and adjustment for multiple comparisons
☐	☒	A full description of the statistical parameters including central tendency (e.g. means) or other basic estimates (e.g. regression coefficient) AND variation (e.g. standard deviation) or associated estimates of uncertainty (e.g. confidence intervals)
☐	☒	For null hypothesis testing, the test statistic (e.g. *F*, *t*, *r*) with confidence intervals, effect sizes, degrees of freedom and *P* value noted<br>*Give P values as exact values whenever suitable.*
☒	☐	For Bayesian analysis, information on the choice of priors and Markov chain Monte Carlo settings
☐	☒	For hierarchical and complex designs, identification of the appropriate level for tests and full reporting of outcomes
☒	☐	Estimates of effect sizes (e.g. Cohen's *d*, Pearson's *r*), indicating how they were calculated

Our web collection on statistics for biologists contains articles on many of the points above.

Software and code

Policy information about availability of computer code

Data collection	Participants are part of the Twins Early Development Study (TEDS), a longitudinal study of twins born in England and Wales between 1994 and 1996. The present study includes data collected in TEDS across multiple waves. Specifically, we analyzed data collected over four waves, when the twins were 7, 9, 12 and 16 years old. Data was collected using paper-pencil, telephone and on line tests and questionnaires. In the paper we provide a brief description of all the measures included in the present study and how they were collected. However, please refer to https://www.teds.ac.uk/datadictionary for detailed descriptions of each measure and information on the items included in each construct.
Data analysis	We used the following software and packages in our data analyses: R package lavaan version 0.6-18 (conducting all Structural Equation Modelling (SEM), such as mediation analyses and multi-level mediation modeling). Packages lavaan, gee, ggplot2 and tidyverse in R. Code is available at https://github.com/CoDEresearchlab/Noncognitive_mediation

For manuscripts utilizing custom algorithms or software that are central to the research but not yet described in published literature, software must be made available to editors and reviewers. We strongly encourage code deposition in a community repository (e.g. GitHub). See the Nature Portfolio guidelines for submitting code & software for further information.

Data

Policy information about availability of data

All manuscripts must include a data availability statement. This statement should provide the following information, where applicable:

- Accession codes, unique identifiers, or web links for publicly available datasets
- A description of any restrictions on data availability
- For clinical datasets or third party data, please ensure that the statement adheres to our policy

The Twins Early Development Study (TEDS) data used in this study are available under restricted access because they contain sensitive human participant data governed by ethical and data protection requirements. Access can be requested through the TEDS data access procedures described at www.teds.ac.uk. Requests are evaluated by the TEDS team and relevant data access committee, and data are made available to qualified researchers for approved research purposes subject to the relevant agreements and approvals. Researchers can apply for access to the TEDS data through their data access mechanism (www.teds.ac.uk/researchers/teds-data-access-policy).

Research involving human participants, their data, or biological material

Policy information about studies with human participants or human data. See also policy information about sex, gender (identity/presentation), and sexual orientation and race, ethnicity and racism.

Reporting on sex and gender	Sex- and gender-based analyses were not conducted because they were not part of the preregistered hypotheses and were not central to the research aims. So that sex was included as a covariate in the analyses. Sex distribution: 51.1% female and 48.9% male.
Reporting on race, ethnicity, or other socially relevant groupings	The families in TEDS are representative of the British population for their cohort in terms of socio-economic distribution, other socially relevant ethnicity and parental occupation.
Population characteristics	Participants are part of the Twins Early Development Study (TEDS), a longitudinal study of twins born in England and Wales between 1994 and 1996. The families in TEDS are representative of the British population for their cohort in terms of socio-economic distribution, ethnicity and parental occupation. Ten thousand families are still actively involved with the TEDS study over twenty years afer the first data collection wave (see57 for additional information on the TEDS sample). The present study includes data collected in TEDS across multiple waves. Recruitment is described in the box below.
Recruitment	Families in England and Wales with twins born between January 1994 and December 1996 were identified using electronic birth records and invited to join TEDS through the Office of National Statistics (ONS). Of the families contacted, 16,810 parents expressed interest in registering their twins to be part of the study, with 13,759 consenting to take part during the first wave when twins were 18 months old. More details are provided in the section below.
Ethics oversight	TEDS has ethical approval from the research ethics committee of Kings College London (references: PNM/09/10–104 and HR/DP-20/21–22060). Consent was obtained before data collection at every wave.

Note that full information on the approval of the study protocol must also be provided in the manuscript.

Field-specific reporting

Please select the one below that is the best fit for your research. If you are not sure, read the appropriate sections before making your selection.

☐ Life sciences ☒ Behavioural & social sciences ☐ Ecological, evolutionary & environmental sciences

For a reference copy of the document with all sections, see nature.com/documents/nr-reporting-summary-flat.pdf

Behavioural & social sciences study design

All studies must disclose on these points even when the disclosure is negative.

Study description	This is a quantitative, developmental study leveraging data collected as part of the Twins early development Study over 9 years, when participants were ages 7,9, 12 and 16.
Research sample	Participants are part of the Twins Early Development Study (TEDS), a longitudinal study of twins born in England and Wales between 1994 and 1996. The families in TEDS are representative of the British population for their cohort in terms of socio-economic distribution, ethnicity and parental occupation. we analyzed data collected over four waves, when the twins were 7, 9, 12 and 16 years old.

Sampling strategy	Families in England and Wales with twins born between January 1994 and December 1996 were identified using electronic birth records and invited to join TEDS through the Office of National Statistics (ONS). According to data from the ONS, there would have been approximately 30,350 multiple births between 1994 and 1996 (Birth Characteristics, 2023; Vital Statistics in the UK, 2021). Of the families contacted, 16,810 parents expressed interest in registering their twins to be part of the study, with 13,759 consenting to take part during the first wave when twins were 18 months old. For detailed information on the TEDS sample, see Lockhart et al. 2023 https://doi.org/10.1002/jcv2.12154
Data collection	Since first contact, data have been collected at 2, 3, 4, 7, 8, 9, 10, 12, 14, 16, 18, 21, and most recently, 26 years. However, budgetary restraints meant the complete sample was not invited to take part in every wave. At ages where the budget was more constrained, the older school cohorts, which contained the majority of TEDS participants, were prioritised. Parents, twins, and teachers have all provided data at various time points, creating a rich multi-informant database. Data linkage efforts, including education records and environmental measures linked using the unique postcodes associated with home addresses, have further extended the TEDS dataset. Twins' parents provided informed consent at each wave of study participation until age 16, after which twins were asked directly. Data have been collected in several forms including paper/pencil questionnaires, telephone and online platforms.
Timing	Core waves of assessment, in which the full contactable sample are invited, took place at ages 4, 7, 8, 12, 14, 16, 18, 21 and 26 years. TEDS is an ongoing study.
Data exclusions	Individuals with severe medical conditions were excluded from analyses. These conditions include detrimental prenatal and postnatal conditions, as well as other conditions that could seriously impact later development. In addition, twins with uncertain and unknown zygosity were excluded from the analyses.
Non-participation	As with any longitudinal cohort study, the TEDS sample has experienced attrition since first contact. However, over 10,000 families remain involved in the study, with more than 6000 families providing data at both ages 21 and 26, see Lockhart et al. 2023 for a detailed description. https://doi.org/10.1002/jcv2.12154
Randomization	Twin analyses compare similarities between monozygotic and dizygotic twins, therefore randomization was not part of these aanalyses.

Reporting for specific materials, systems and methods

We require information from authors about some types of materials, experimental systems and methods used in many studies. Here, indicate whether each material, system or method listed is relevant to your study. If you are not sure if a list item applies to your research, read the appropriate section before selecting a response.

Materials & experimental systems

n/a	Involved in the study	
☒	☐	Antibodies
☒	☐	Eukaryotic cell lines
☒	☐	Palaeontology and archaeology
☒	☐	Animals and other organisms
☒	☐	Clinical data
☒	☐	Dual use research of concern
☒	☐	Plants

Methods

n/a	Involved in the study	
☒	☐	ChIP-seq
☒	☐	Flow cytometry
☒	☐	MRI-based neuroimaging

Plants

Seed stocks	*Report on the source of all seed stocks or other plant material used. If applicable, state the seed stock centre and catalogue number. If plant specimens were collected from the field, describe the collection location, date and sampling procedures.*
Novel plant genotypes	*Describe the methods by which all novel plant genotypes were produced. This includes those generated by transgenic approaches, gene editing, chemical/radiation-based mutagenesis and hybridization. For transgenic lines, describe the transformation method, the number of independent lines analyzed and the generation upon which experiments were performed. For gene-edited lines, describe the editor used, the endogenous sequence targeted for editing, the targeting guide RNA sequence (if applicable) and how the editor was applied.*
Authentication	*Describe any authentication procedures for each seed stock used or novel genotype generated. Describe any experiments used to assess the effect of a mutation and, where applicable, how potential secondary effects (e.g. second site T-DNA insertions, mosiacism, off-target gene editing) were examined.*
