# Supplementary material for: Non-cognitive skills mediate education-related polygenic score associations with academic achievement across development
Source: Nat Commun. 2026 May 8;17:5133. doi: 10.1038/s41467-026-72838-2 (PMC13249983; doi:10.1038/s41467-026-72838-2)
Supplement: Supplementary file 5 — Transparent Peer Review file [file 41467_2026_72838_MOESM5_ESM.pdf]

nature portfolio

Peer Review File

# Non-cognitive skills mediate education-related polygenic score associations with academic achievement across development

Corresponding Author: Dr Quan Zhou

**This file contains all reviewer reports in order by version, followed by all author rebuttals in order by version.**

Version 0:

Reviewer comments:

Reviewer #1

(Remarks to the Author)
This paper makes a valuable and timely contribution to the current literature. The education polygenic score is among the most widely used polygenic scores and a robust predictor of educational (and other important) outcomes. With a complex social outcome such as educational attainment, it is clear that genetics do not exert direct effects in isolation. Rather, multiple, complex, and interacting pathways likely unfold across development to translate genetic propensity into educational outcomes. Yet, despite the widespread use of the education PGS, very little is known about the potentially mediating mechanisms as strikingly few studies have examined them.

In this context, the paper fills a critical gap by examining a wide range of plausible non-cognitive mediators that (to my knowledge) have not been previously tested. The manuscript is rigorous, clear, and well-written, with comprehensive and detailed supplementary materials that complement the main text. However, there are several points that I believe need to be addressed or clarified before the work is disseminated:

**Abstract**

The statements “up to 65%” (p. 1, line 29) and “up to 83%” (p. 1, line 31) highlight only the upper bounds of the mediation effects. It would be helpful to present the full range of estimated effects to give a more complete and transparent picture of the findings.

**Introduction**

In the paragraph on gene-environment correlation (p. 2, lines 57–73), the suggestion that mediation via family environments likely reflects passive rather than evocative processes is consistent with work colleagues and I published last year (Austerberry et al., 2024, https://doi.org/10.1111/cdev.14142). Using an adoption sample (which by design controls for passive gene-environment correlation), we found that the home environment (warm parenting, household chaos, time spent watching TV) did not significantly mediate the effects of education-linked genetic factors on children’s educational performance in middle childhood.

“Non-cognitive skills independently contribute to educational success beyond cognitive ability.” (p. 2, lines 72–80) — I recommend softening this to avoid overly causal language. For example, by adding “appear to” after “skills” or replacing “independently contribute to” with “are associated with”. Similarly, in the statement “… self-efficacy and personality traits significantly influenced academic achievement beyond cognitive ability”, I would suggest replacing “influenced” with something like “were significantly associated with”.

The comment that “… previous research showed that greater self-control… partly explains the genetic link to educational attainment.” (p. 2, lines 84–86) could be tempered slightly as the literature in this area is somewhat mixed. For example, a few years ago, colleagues and I examined, in an adoption sample, whether language or self-regulation (inhibitory control) mediated the effects of children’s cognitive/education-associated genetic propensities. We found that language—but not inhibitory control—was a significant mediator of genetic effects on children’s educational performance in middle childhood

(Austerberry et al., 2021, https://doi.org/10.1111/cdev.13706).

**Results**

The first paragraph, "Latent dimensions of non-cognitive skills" (p.3, lines 111–117), seems more appropriate for the Methods section than the Results.

I found it difficult to interpret the descriptive statistics referenced in the second paragraph of the Results (p. 3, lines 120–121), Supplementary Note 1, and Supplementary Table 1, for a couple of reasons: (1) There is no narrative summary of the key descriptive statistics. (2) The numbers in Supplementary Table 1 do not correspond with the measures descriptions in the Methods. For instance, the Methods describe achievement at 16 as "coded on a scale from 11 (A*) to 4 (G, the lowest passing grade)", yet the mean value reported in the supplementary table is 0.018 (which is not a number on that scale). This makes it difficult to interpret where the sample lies on the grade distribution. The same issue arises with other measures in the table — the scoring ranges and directionality are not clearly indicated and do not seem to correspond with the scaling outlined in the measures section. It would be helpful to include a brief narrative summary of the key descriptive statistics in either the main text or the Supplement, and to align the values in Supplementary Table 1 with the measures descriptions in the Methods.

Regarding the sentence, "…correlations between polygenic scores and academic achievement at all ages were statistically significant, moderately sized (Supplementary Figure 1) and increased across development…" (p. 3, lines 129–131):
(1) Could the authors confirm (as the p-values are not shown) that all correlations in Supplementary Figure 1 were statistically significant?
(2) Describing the correlations as "moderately sized" seems generous, as many are < 0.2 (including some < 0.1) and the conventional threshold for a moderate correlation tends to be 0.3–0.6. This is also true for the correlations with non-cognitive skills.
(3) It may be more accurate to say that the correlations "tended to" increase across development, as not all correlations increased with each developmental timepoint (i.e., not all increases were monotonic).

"The indirect (mediated) effects of education-specific non-cognitive skills at age 9 were larger for parent-rated ($\beta = 0.05$ [95% CI, 0.02, 0.07])" (p. 4, lines 148–150) — Since the confidence intervals for the parent-rated and self-rated effects overlap, I would avoid calling one "larger", as the difference is not statistically significant.

**Methods**

More detail is needed on the measures. Either in the main text or Supplement, it would be helpful to provide consistent information across all measures on: who the rater was, number of items, response scale (e.g., 3-point Likert scale from 1, "Certainly True", to 3, "Not True"), whether items were reverse scored, and what high/low scores indicate (e.g., did higher scores indicate higher/lower perceived ability). This would help readers interpret the descriptive statistics in Supplementary Table 1 more easily. Including information on the reliability and validity of the measures would also strengthen this section.

The Strengths and Difficulties Questionnaire (SDQ) tends to be described as a measure of emotional, behavioural, and relationship functioning, rather than self-regulation. While some researchers have used select SDQ items as indicators of dysregulation (e.g., Holtmann et al., 2011, https://doi.org/10.1159/000318164), the full measure is not typically considered a measure of self-regulation.

Did you have a pre-specified cutoff for acceptable model fit? Several of the CFAs showed poor model fit—particularly:
•General cognitive factor at age 9 (Supplementary Table 23),
•Self- and teacher-reported non-cognitive skills at age 9 (Supplementary Table 24),
•Self-reported non-cognitive skills at age 16 (Supplementary Table 24),
•Teacher- and self-rated SDQ scores at ages 9, 12, and 16 (Supplementary Table 25).
It would be worth investigating why model fit is poor in these cases and considering potential adjustments.

I was unsure whether age and sex were correctly specified in your CFAs. In Supplementary Tables 24 and 25, it appears that they were included as indicators of the latent factors. If the intent was to control for age and sex, they should be entered as exogenous predictors of the latent variables rather than endogenous indicators.

How many bootstrap samples were used in the mediation models? I am assuming from your code that you used the lavvan default of 1000 but it would be helpful to specify.

I could not find mention of how you handled missing data. In the supplementary mediation results, it looks like you used listwise deletion, resulting in substantial data loss. However, in your code, it looks as though you used the lavaan default—which I think is FIML (which is usually the preferable approach for reducing the risk of bias and power loss). Please could you add to the Methods details on whichever approach you took.

With these revisions, I believe the manuscript will make a strong and impactful contribution.

Chloe Austerberry

(Remarks on code availability)

I was able to access and understand the code. The code seemed useable and the analyses reproducible.

Reviewer #2

(Remarks to the Author)
This study explores whether so called non-cognitive skill may act as mediators between polygenic risk score and academic achievement in TEDS sample. In general the paper is well-written and the authors are obviously experts of the genetic methodology and sample they use.
I am not an expert of studies with DNA methods and cannot evaluate the decisions made regarding the polygenic risk scores. My evaluation focuses more on the conceptual issues. I have two main issues with what is done in the manuscript and how the results are interpreted: Both are related to the measures;
The study outcome is academic performance evaluated by teachers at ages 7 (English, mathematics), 9, and 12 (English, mathematics, and science). At age 16, mean grades for English, mathematics, and science was used.
The so called non-cognitive skills included:
-at age 9: academic self-perceived ability , academic interest and the Classroom Environment Questionnaire (satisfaction with classroom; enjoyment of learning, educational opportunities)
- at age 12: academic interest, academic self-perceived ability, the literacy environment questionnaire and the mathematics environment questionnaire
-at age 16: academic self-concept, school engagement, grit, academic ambition, time spent on math, math self-efficacy, math interest, curiosity, and attitudes toward school
Cognitive ability included mainly language and visuo-spatial tasks from WISC. Raven, etc.
First, the non-cognitive skills are not skills, and some are not even characteristics of the child/adolescent. Satisfaction with classroom may not have anything to do with the child, for example. Sometimes teachers and classrooms are indeed not that great and it has nothing to do with the skills of the student in the “non-cognitive skill “domain.
Secondly, and more importantly, this study does not include assessments of the actual reading, mathematics, or science skills. The cognitive skills included are known to be surprisingly weak correlates of reading and math skills and difficulties. Therefore, including them does not help with covarying the cognitive or skill variance in the equations. At the same time, the so-called non-cognitive measures are known to be strongly correlated with the skills and the basic academic skills are correlated also with the outcome. Therefore, the found associations/mediation results may be confounded by the student’s skills in reading and math. Particularly the included motivation-measures and self-reported skills (self-concept of ability and self-efficacy -type measures) share much variance with the basic academic skills. Without including assessments of the skills, these results should not be interpreted as evidence for non-cognitive mediation.
The problem is also to be seen in the interpretations/conclusions; authors conclude that “This might be particularly useful knowledge for interventions aimed at fostering learning. Interventions aimed at enhancing students' academic self-concept, interests, and motivations, such as inquiry-based learning42, positive feedback43, and goal-setting44, might be particularly effective.” First of all, this conclusion is very far from what was examined. Also, based on the available evidence in literature I’m not convinced yet if we should indeed focus on these targets when children’s skills are not in place. Tis is also what these results can conclude. For example, studies examining the associations between reading and math skills and self-concept of ability development suggest that skills predict self-concept appraisals and not the other way around. Same is true for interest in reading. This suggest that motivation and self-beliefs follow skills and if the main target of education is skilled people, then we should rather focus on skills that (false) self-beliefs.

(Remarks on code availability)

Reviewer #3

(Remarks to the Author)
This manuscript is exceptional in many ways. It reports findings from very sophisticated analyses addressing extremely important and well-conceived questions. The findings could inform future interventions to improve educational outcomes within the context of gene-environment correlations. Moreover, the important issues and findings are described in one of the best-written manuscripts I have read in decades. That said, I have some suggestions and serious concerns.
1.The manuscript misrepresents the representativeness of the sample. The sample was not selected to be representative of any population and one cannot claim that dizygotic twins are representative of the general population. That statement needs to be amended.
2. The use of latent variables derived from multiple variables obtained from multiple informants (that differed by wave) is mathematically defensible, but the manuscript needs to warn readers that this bird's eye view of the potential role of non-academic mediators of genetic propensities could be misleading. Readers need to understand that the findings do not tell us if any single variable from a given informant is related to academic achievement. This is extremely important in helping readers translate findings into interventions. Far more detailed analyses would be needed to know which components of latent variables, if any, might be good targets for interventions.
3. I am not at all convinced that a latent variable derived from the variables scales of the Strengths and Difficulties Questionnaire provides a meaningful measure of "self-regulation." This might explain why that construct was not found to be a strong mediator. The factor analysis creates a single latent score based on several psychopathology scales and adaptive prosocial personality. I wonder if the results would have been different had prosociality not been included and a bifactor

model had been used to distinguish general and specific factors. I would like to see the analyses of the SDQ addressed in more detail, perhaps in a separate paper. I would be very surprised if some aspects of maladaptive attention, emotion, and conduct did not mediate the predictions based on the EA PGS.

(Remarks on code availability)

Reviewer #4

(Remarks to the Author)
The study by Zhou et al. examines how non-cognitive skills (as reported by children, parents, and/or teachers) such as self-regulation, learning attitudes and strategies mediate the associations between polygenic indexes for cognitive ability, non-cognitive traits and educational attainment, and subsequent academic achievement in English, maths, and science at ages 7, 9, 12, and 16. The central questioned being tackled is an important one – through what pathways and processes does genetic liability unfold across development to influence academic outcomes? By utilising the twin structure of the cohort the study is also able to control for shared family environment, population stratification and assortative mating, which is a noted strength, as are the multiple informants. While the practical implications are likely some way off, this work generates testable hypotheses about which interventions - and who they should target, and when - might moderate genetic risk to poorer educational outcomes. From my perspective, these findings also underscore the need to account for genetic confounding in measures of student skills and behaviours; teacher-reported measures are free from passive gene–environment correlation, which might make them especially valuable for examining indirect paths (?).

While we find the overall methodological approach appropriate —and note the use of a well-characterised, large longitudinal twin cohort and pre-registration of analyses—the analytical strategy and variable choices are poorly justified, and the relationship of these findings to the group's prior work is not clearly articulated. Our major comments focus on the following:

**Clarify overlap with previous published research**
The authors cite two prior papers from their group (refs 29 and 17) but do not clearly explain how this study differs from or extends them. From a skim, much of the current manuscript appears to replicate results reported in Malanchini et al. (2024), using the same cohort, timepoints, and overall design to test very similar hypotheses—only without a mediation analysis? This overlap should be explicitly acknowledged, with a clear statement of what is novel here.

e.g. lines 329-331-replicating results from ref 29- but not cited, framed as novel finding "the mediating role of education-specific non-cognitive skills increased developmentally, pointing to the growing importance of students' perceived non-cognitive profiles and experiences in their academic journeys."
vs

Malanchini et al. (2024) discussion: "independent genetic contribution of non-cognitive skills to academic achievement increased developmentally"

Relatedly, the manuscript cites Zhou et al. (2024; ref. 17), which seems to employ the same cohort, PGIs, and outcome measures (?) but focuses on family-environment mediators rather than non-cognitive skills. The authors should explicitly state how the present study's findings converge with—or diverge from—those reported by Zhou et al. Drawing these connections will help readers understand how the current study builds on the existing research and will help clarify its unique contribution.

**Choice of PGIs**
It isn't made clear why it is important to look at EA PGI alongside the cog and non-cog PGIs (given the EA PGI is so powerful, what does adding the cognitive PGI tell you?), or how the five PGIs relate (or not) to one another. For example, should the EA and cognitive PGIs be more highly correlated (and show more similar patterns of association) than the non-cognitive PGIs? In what ways does the Demange et al. (ref 31) versus Malanchini et al (ref 29) 'non-cognitive' PGIs differ from each other? Should the reader expect overlapping patterns of associations?
A brief description of why each of the five PGIs were selected, alongside how each PGI was derived—and a correlation matrix showing the inter-relations among the five PGIs—would help the reader interpret the observed patterns of association in the manuscript.

**Choice of mediator variables**
An explanation of inclusion criteria for behavioural dimensions included in the mediator analysis is missing. Was there a specific rationale for the inclusion/exclusion of certain measures, and how were they systematically rated as cognitive vs non-cognitive, and beyond this, education-specific vs non-education specific? For example, if you ask students how good they are at school, their answers will track with how well they actually do – so what are the conceptual and/or data-driven distinctions being made between cognitive and non-cognitive skills?

**Latent factors vs. individual measures**
The manuscript would benefit from a clearer justification and more consistent reporting regarding the use of both latent-factor

composites and individual non-cognitive measures. For instance, why build latent factors and also analyse individual scores? What distinct insights are gained, or which set of results should the reader focus on? We note that Supplementary Table 24 shows that some latent models (e.g. child- and parent-reported non-cognitive skills at age 9) fit poorly, and some do not include all individual measures (age 16). The model fits should be reported so readers understand the strength—and limitations—of the latent constructs before interpreting mediation results. Finally, there seems to be inconsistency across analyses with only education specific NCS mediation reported for individual measures, but not self-regulation?

Minor comments

Abstract
The abstract's focus on mediation at age 9 appears a little cherry-picked (since effects aren't consistent across reporters at age 9?) and overlooks how small the overall PGI–achievement associations can be (e.g., ~0.10 for teacher-rated achievement at age 9 with the non-cognitive PGI). To avoid overstating the findings, please report the absolute size of the indirect (mediated) effect or the percentage of total variance it explains—so readers can see that, despite reported proportions, the effects themselves are modest.

Introduction
As noted, the introduction requires more contextualisation for the study and clearer motivation for the section of variables and analytical strategy. Some of this appears in the Discussion (e.g. lines 366–370, 402–404); it should be introduced and expanded in the Introduction.
The manuscript cites many papers based on the cohort examined in the current study. To strengthen your argument(s), please incorporate findings from other cohorts beyond TEDS where possible.
The key question 3 (lines 91–92 – 'importantly') inadvertently downplays your first two questions. I suggest repositioning this question higher, with more context as to why this is a salient question to ask.

Results
The manuscript utilises non-cognitive skills data obtained from multiple raters, including teacher, parents, and the children themselves (line 103). Could they be dealt with systematically rather than interleaving them, for clarity? That way readers can compare across informants and more easily note where there are gaps.
In a similar vein, the ordering and orientation of figures (e.g., Figure 1 vs. Figure 2 vs Fig 3) are inconsistent, which hampers cross-comparison. Please present mediation figures in the same layout.
Lines 111-118: as mentioned, given the focus of the manuscript, greater transparency is needed about how the CFA was selected, model fits should be stated and if any measures were excluded and why.

Lines: 123-126. I found this generally unclear – I initially read this as three PGIs, but its actually 5…?

Line 130: This line states that all correlations between PGIs and achievement were statistically significant, yet the p-values are not reported in Supp Fig 1. Some of the reported correlations are near zero … (e.g. -0.01)?

Line 132: The stated range is wrong? (it's 0.04-0.22)
Line 134: why isn't the range also presented here for consistency?
Line 135: Now only three PGIs are reported? I am unsure why.
Line 140: Please check if p-values really are exactly zero (across all supp tables)
Line 149: ..'increased across compulsory education within reporter would arguably be a more accurate way to describe the pattern of results in Fig 1? (i.e., indirect effects at age 9 for teacher reported achievement are ~x2 that at age 16)
Line 160: As noted, in my earlier comment that the total effects are sometimes small..
Line 162: ok, so now the results for Demange et al PGIs are being reported separately, but in a different format (tables 5 and 6). Presenting these data in the same format as Figure 1 would facilitate comparison of results across PGIs.

Line 177: As mentioned, can you clarify why you test both latent-factor composites and individual measures, especially since several latent-factor indirect effects (e.g. age-9 self-report with the non-cognitive PGI) were non-significant—and in some cases the PGI doesn't seem to correlate with achievement (e.g. non-cog PGI at age 9)?

Line 190-193: While several NCSs (e.g. math interest, curiosity, academic self-concept) show substantial indirect effects, many of the measures at age 16 do not show significant mediation—yet this narrative overlooks these null findings. To avoid giving a skewed impression, please revise.

Figure 2: The bottom left panel lists several age 16 measures (optimism, openness and conscientiousness) that are not mentioned elsewhere in the paper and so appear entirely new. Please clarify.

Line 221: Malanchini et al. (2024) states future work should explicitly investigate developmental change at the phenotypic, genetic and genomic level, suggesting latent growth curve models (LGCM). The current manuscript uses a simple time-lagged longitudinal model to assess how early skills mediate later polygenic score effects. Can the rationale for this choice of analytical approach over LGCM be provided?

Line 256: Can Supp Tables 14-16 be provided as a figure (akin to Fig 2). For example, table 16 has 4,700 rows making it very difficult to interpret.

Line 360: Can you please use PGI/PGS, not genetics (as you are examining PGS that only capture some of the heritability.

Methods
Line 444: The numbers reported here differ to those reported in Supp note 1 and Supp table 1?

Line 486: Age 12 only has teacher report, not all three raters?

TYPOS

258 child, rather than shared by siblings, were more likely to be mediated *BY parents' perceptions

353 emphasised the critical role of intrinsic, student-*CENTRED non-cognitive skills (e.g., intellectual curiosity and academic self-concept) in gene-environment transactions

(Remarks on code availability)

Reviewer #5

(Remarks to the Author)
I co-reviewed this manuscript with one of the reviewers who provided the listed reports. This is part of the Nature Communications initiative to facilitate training in peer review and to provide appropriate recognition for Early Career Researchers who co-review manuscripts.

(Remarks on code availability)

Version 1:

Reviewer comments:

Reviewer #1

(Remarks to the Author)
I have reviewed the revised manuscript and the accompanying response to reviewers. The authors have done an excellent and thorough job of addressing all of my comments, as well as those raised by the other reviewers. The revisions have substantially strengthened the manuscript, and my previous concerns have been fully resolved.

I am now satisfied with the manuscript in its current form and have no further substantive comments or requests for revision. This paper fills a critical gap in our knowledge of potentially modifiable pathways though which genetic effects may be mediated across development, and I am happy to recommend the paper for publication.

Chloe Austerberry

(Remarks on code availability)

Reviewer #2

(Remarks to the Author)
Thank you for the clear responses on my queries and concerns.

Congratulations on the excellent paper!

(Remarks on code availability)

Reviewer #3

(Remarks to the Author)
NCOMMS25_19683A
I continue to see merit in this manuscript, but I am sorry to say that I am less positive than I was after my first review. This is partly because the other reviewers identified issues that I had not seen, but it is mostly because the author has not been responsive to the key issues raised by the other reviewers and myself.
1. I previously noted that one cannot refer to the TEDS sample as "representative." The author's response to my critique suggests that they think "representative" means "similar to the rest of the population." It does not. It means that every twin pair

was recruited in a simple or complex design that gave each pair of twins in the population an equal chance of participation. That is not how the TEDS sample was recruited and it cannot be referred to as representative. Any sampling method other than representative sampling introduces bias into the sample, often unknowable biases.
2. In my previous review, I raised concerns about the measures and their interpretations. I thought I was being clear at that time, but I see that I was too being polite. At the risk of being blunt, therefore, I will be more clear. There is NO measure of "self-regulation" analyzed for this paper. Any reference to self-regulation is completely misleading. It is absolutely not defensible to refer to the SDQ as a measure of "self-regulation." It is a heterogeneous measure of behavior problems, emotional problems, and prosocial behavior. Although some economists have naively referred to SDQ scores as reflecting failures of self-regulation, that hypothesis has never been supported and almost certainly could never be confirmed. I cannot endorse a manuscript that refers to the SDQ as a measure of self-regulation.
3. I was convinced by the other reviewers that the term "non-cognitive skills" is a misnomer. This is partly because constructs such as self-concept and academic interest cannot reasonably be termed "skills." It is also because it is probably impossible to draw a clear line between cognitive and non-cognitive aspects of behavior, as cognition is part of nearly every aspect of behavior. Furthermore, at least some of the self-concept measures are likely to be results of variations in academic performance rather than mediators. I would be more comfortable with something like "non-academic characteristics."
4. There are a number of typos, such as "overtime" instead of "over time" in line 102.

(Remarks on code availability)

Reviewer #4

(Remarks to the Author)

Thank you for the detailed and thorough response to our review. We appreciate the care taken to address the comments. We have no further concerns and are happy to recommend the paper for publication

(Remarks on code availability)

Reviewer #5

(Remarks to the Author)
I co-reviewed this manuscript with one of the reviewers who provided the listed reports. This is part of the Nature Communications initiative to facilitate training in peer review and to provide appropriate recognition for Early Career Researchers who co-review manuscripts.

(Remarks on code availability)
I co-reviewed this manuscript with one of the reviewers who provided the listed reports. This is part of the Nature Communications initiative to facilitate training in peer review and to provide appropriate recognition for Early Career Researchers who co-review manuscripts.

Version 2:

Reviewer comments:

Reviewer #3

(Remarks to the Author)
The authors responded well to my previous comments and I have no further concerns.

(Remarks on code availability)

**Open Access** This Peer Review File is licensed under a Creative Commons Attribution 4.0 International License, which permits use, sharing, adaptation, distribution and reproduction in any medium or format, as long as you give appropriate credit to the original author(s) and the source, provide a link to the Creative Commons license, and indicate if changes were made.
In cases where reviewers are anonymous, credit should be given to 'Anonymous Referee' and the source.
The images or other third party material in this Peer Review File are included in the article's Creative Commons license, unless indicated otherwise in a credit line to the material. If material is not included in the article's Creative Commons license and your intended use is not permitted by statutory regulation or exceeds the permitted use, you will need to obtain permission directly from the copyright holder.
To view a copy of this license, visit https://creativecommons.org/licenses/by/4.0/

**Queen Mary University of London**
Department of Biological and Experimental Psychology
School of Biological and Behavioural Sciences
Mile End Road
London E1 4NS

London, 14th November 2025

The Editorial Team
Nature Communication
Subject: Article submission.

Dear Editors,

Thank you for the opportunity to revise and resubmit our manuscript: "***From genetic disposition to academic achievement: The mediating role of non-cognitive skills across development***" to *Nature Communications*. We are grateful for the valuable feedback received from five reviewers. Please find below a point-by-point response letter.

Sincerely,
Quan Zhou and Margherita Malanchini

School of Biological and Behavioural Sciences
Queen Mary University of London

---

Reviewer #1 (Remarks to the Author):

This paper makes a valuable and timely contribution to the current literature. The education polygenic score is among the most widely used polygenic scores and a robust predictor of educational (and other important) outcomes. With a complex social outcome such as educational attainment, it is clear that genetics do not exert direct effects in isolation. Rather, multiple, complex, and interacting pathways likely unfold across development to translate genetic propensity into educational outcomes. Yet, despite the widespread use of the education PGS, very little is known about the potentially mediating mechanisms as strikingly few studies have examined them.

In this context, the paper fills a critical gap by examining a wide range of plausible non-cognitive mediators that (to my knowledge) have not been previously tested. The manuscript is rigorous, clear, and well-written, with comprehensive and detailed supplementary materials that complement the main text.

*Response: We thank Dr. Austerberry for this positive evaluation of our work.*

**Abstract**

1) The statements "up to 65%" (p. 1, line 29) and "up to 83%" (p. 1, line 31) highlight

only the upper bounds of the mediation effects. It would be helpful to present the full range of estimated effects to give a more complete and transparent picture of the findings.

*Response: Thank you for this suggestion. We have revised the abstract accordingly (see excerpt below):*

*'The role of environmental, developmental, and psychological processes in translating genetic dispositions into observed academic achievement remains under-investigated. This study examined whether non-cognitive skills—including motivation, attitudes and self-regulation—mediate genetic effects on academic achievement across development. Data were collected from 5,016 children enrolled in the Twins Early Development Study at ages 7, 9, 12, and 16, as well as their parents and teachers. We found that non-cognitive skills mediated between less than 5 and up to 64% of the genetic prediction of academic achievement and that effects were larger and more robust for motivation and attitudes (ß ≈ 0.13) than for self-regulation (ß ≈ 0.01–0.03). This pattern of results held longitudinally and was replicated in within-family analyses, where non-cognitive skills accounted for up to 83% of the total mediation effects. This highlights the contribution of non-cognitive skills beyond shared familial factors, likely reflecting how children evoke and select experiences that align with their genetic propensity and lead to differences in academic development.' [Page 1 of the revised manuscript]*

**Introduction**

2) In the paragraph on gene-environment correlation (p. 2, lines 57–73), the suggestion that mediation via family environments likely reflects passive rather than evocative processes is consistent with work colleagues and I published last year (Austerberry et al., 2024, https://doi.org/10.1111/cdev.14142). Using an adoption sample (which by design controls for passive gene-environment correlation), we found that the home environment (warm parenting, household chaos, time spent watching TV) did not significantly mediate the effects of education-linked genetic factors on children's educational performance in middle childhood.

*Response: We thank Dr. Austerberry for this suggestion, we have included this highly relevant paper in the revised draft:*

*'Consistent with this interpretation, an adoption study that controls for passive gene–environment correlation by design found no evidence that family environments such as warm parenting, household chaos, or time spent watching TV mediated education-linked genetic effects on children's academic performance (18)' [Page 2 of the revised manuscript]*

3) "Non-cognitive skills independently contribute to educational success beyond cognitive ability." (p. 2, lines 72–80) — I recommend softening this to avoid overly causal language. For example, by adding "appear to" after "skills" or replacing

**Queen Mary University of London**
Department of Biological and Experimental Psychology
School of Biological and Behavioural Sciences
Mile End Road
London E1 4NS

"independently contribute to" with "are associated with". Similarly, in the statement "… self-efficacy and personality traits significantly influenced academic achievement beyond cognitive ability", I would suggest replacing "influenced" with something like "were significantly associated with".

*Response: Thank you for this suggestion, we have modified the draft accordingly (see excerpt below).*

*'Non-cognitive skills are associated with educational success beyond cognitive skills. Studies have found that characteristics such as self-efficacy and personality predict academic achievement beyond cognitive ability across compulsory education (25,26)' [Page 2 of the revised manuscript]*

4) The comment that "… previous research showed that greater self-control… partly explains the genetic link to educational attainment." (p. 2, lines 84–86) could be tempered slightly as the literature in this area is somewhat mixed. For example, a few years ago, colleagues and I examined, in an adoption sample, whether language or self-regulation (inhibitory control) mediated the effects of children's cognitive/education-associated genetic propensities. We found that language—but not inhibitory control—was a significant mediator of genetic effects on children's educational performance in middle childhood (Austerberry et al., 2021, https://doi.org/10.1111/cdev.13706).

*Response: Thank you for this comment and for drawing our attention to the mixed evidence regarding self-control. We have revised the manuscript accordingly and in the revised draft we emphasize the importance of distinguishing between different facets of non-cognitive skills (e.g., broad self-control vs. narrower regulatory processes such as inhibitory control). We have also now referenced the highlighted paper. See excerpt below:*

*'Furthermore, previous research showed that greater self-control and, to a lesser extent, interpersonal skills, partly explained education-associated genetic effects on adult educational attainment (31). However, findings are mixed. For example, an adoption study that considered the mediating role of inhibitory control, a more targeted facet of self-regulation, found no evidence for mediation effects on educational performance in middle childhood (32)' [Page 3 of the revised manuscript]*

**Results**

5) The first paragraph, "Latent dimensions of non-cognitive skills" (p.3, lines 111–117), seems more appropriate for the Methods section than the Results.

*Response: Thank you for this thoughtful suggestion. We agree that the paragraph provides a background on the latent structure of non-cognitive skills, and as such, could align with the Methods section. We have shortened the paragraph and only retained the essential information in the Results section to provide only the*

Queen Mary University of London
**Queen Mary University of London**
Department of Biological and Experimental Psychology
School of Biological and Behavioural Sciences
Mile End Road
London E1 4NS

*necessary context for the reader to appreciate our empirical findings (see the revised paragraph below).*

*'**Creating latent dimensions of non-cognitive skills***
*Different measures of non-cognitive skills were collected at different ages, with ratings provided by children, parents, and teachers. Following our previous work (26), two latent dimensions of non-cognitive skills were created using confirmatory factor analyses: education-specific non-cognitive skills (including attitudes towards school, self-perceived academic ability, academic interest and curiosity) and self-regulation (encompassing behavioural and emotional regulation). A detailed description is provided in the Methods.*

*We have added a more detailed explanation of how the latent constructs were derived to the revised Methods section. Please see excerpt below:*

*'**Construction of latent factors of non-cognitive skills and general cognitive ability***
*Confirmatory factor analysis (CFA)(89) was used to create latent constructs of general cognitive ability and non-cognitive skills across developmental stages. In alignment with established research on general cognitive ability (g) and prior investigations in the TEDS dataset (90), we modelled a single factor capturing general cognitive ability (g) at each developmental stage. Each g factor was constructed from the weighted loadings of two verbal and two nonverbal tests (see Supplementary Table 23).*

*CFA was also used to create dimensions reflecting non-cognitive skills. Drawing from previous meta-analytic research on the non-cognitive factors influencing educational outcomes, we adopted a theoretical differentiation between education-specific non-cognitive traits (such as academic related attitudes, motivation, and goals) and broader, more universally applicable measures of self-regulation (encompassing behavioural and emotional regulation). Consequently, distinct factors were established for a) education-specific non-cognitive skills (NCS) and b) domain-general self-regulation skills, considering the various measures available at each age for each evaluator. Full details of latent factor construction and model fit indices are reported in Supplementary Note 3 and Supplementary Table 24 and 25.' [Page 19 of the revised manuscript]*

6) I found it difficult to interpret the descriptive statistics referenced in the second paragraph of the Results (p. 3, lines 120–121), Supplementary Note 1, and Supplementary Table 1, for a couple of reasons: (1) There is no narrative summary of the key descriptive statistics. (2) The numbers in Supplementary Table 1 do not correspond with the measures descriptions in the Methods. For instance, the Methods describe achievement at 16 as "coded on a scale from 11 (A*) to 4 (G, the lowest passing grade)", yet the mean value reported in the supplementary table is 0.018 (which is not a number on that scale). This makes it difficult to interpret where the sample lies on the grade distribution. The same issue arises with other measures

**Queen Mary University of London**
Department of Biological and Experimental Psychology
School of Biological and Behavioural Sciences
Mile End Road
London E1 4NS

in the table — the scoring ranges and directionality are not clearly indicated and do not seem to correspond with the scaling outlined in the measures section. It would be helpful to include a brief narrative summary of the key descriptive statistics in either the main text or the Supplement, and to align the values in Supplementary Table 1 with the measures descriptions in the Methods.

*Response: Thank you for this helpful and detailed comment. We agree that the descriptive statistics in Supplementary Table 1 were difficult to interpret without clarification. As part of our standard preprocessing pipeline, all continuous variables, including academic achievement, non-cognitive skills, and cognitive ability, were residualised for age and sex and subsequently standardised before analysis. As a result, the values shown in Supplementary Table 1 reflect these standardised and adjusted variables, rather than the original raw scales described in the Methods section (e.g., GCSE grades).*

*To address this, we have now:*

*1) Added an explicit explanation in the data preparation section of the Methods:*

*'To control for potential confounding factors, we regressed all continuous variables (academic achievement, non-cognitive skills, and general cognitive ability) on age and sex collected at the same wave and we used the standardised residuals in all our analyses. Supplementary Table 1 presents descriptive statistics for the standardised and age and sex-adjusted variables' [Page 19 of the revised manuscript]*

*2) Clarified this in the Note of Supplementary Table 1:*

*'All continuous variables (academic achievement, non-cognitive skills, and general cognitive ability) were residualised for age and sex at the same wave and then standardised. Values reflect the standardised, age- and sex-adjusted variables used in the analyses'.*

*3) Included a brief narrative summary of the descriptive statistics in the Results section:*

*'Descriptive statistics are presented in Supplementary Note 1 and Supplementary Table 1. All variables were normally distributed; therefore, no transformations were applied prior to analyses.'*

*4) we describe the statistics in greater detail in Supplementary Note 1:*

*'Descriptive statistics for the variables used in this study are presented in Supplementary Table 1. These include age- and sex-adjusted standardised scores for academic achievement, non-cognitive skills (both individual indicators and latent factors), cognitive ability, and polygenic scores, collected from different raters across*

**Queen Mary University of London**
Department of Biological and Experimental Psychology
School of Biological and Behavioural Sciences
Mile End Road
London E1 4NS

*multiple developmental stages. Only participants with available genotype data were included in the final analytic sample (N range = 1,293–5,016). All variables were approximately normally distributed.' [Page 3 of the revised Supplementary Information]*

7) Regarding the sentence, "…correlations between polygenic scores and academic achievement at all ages were statistically significant, moderately sized (Supplementary Figure 1) and increased across development…" (p. 3, lines 129–131):
(1) Could the authors confirm (as the p-values are not shown) that all correlations in Supplementary Figure 1 were statistically significant?
(2) Describing the correlations as "moderately sized" seems generous, as many are < 0.2 (including some < 0.1) and the conventional threshold for a moderate correlation tends to be 0.3–0.6. This is also true for the correlations with non-cognitive skills.
(3) It may be more accurate to say that the correlations "tended to" increase across development, as not all correlations increased with each developmental timepoint (i.e., not all increases were monotonic).

*Response: Thank you for raising these points. To improve clarity and transparency, instead of reporting a correlation matrix, we have included a table (Supplementary Table 2a), which reports the exact correlation coefficients and p-values. We have also revised the text in the Results section to describe how all correlations between polygenic scores and academic achievement were statistically significant and varied from very small to moderate in size, with magnitudes tending to increase across development.*

*'Correlations between polygenic scores and academic achievement at all ages were statistically significant (Supplementary Table 2a). Their magnitude tended to increase across development, particularly for the EA polygenic score (r ranging from 0.23 at age 7 to 0.35 at age 16) and the NonCog polygenic score (r ranging from 0.11 at age 7 to 0.24 at age 16).' [Page 4 of the revised manuscript]*

8) "The indirect (mediated) effects of education-specific non-cognitive skills at age 9 were larger for parent-rated (β = 0.05 [95% CI, 0.02, 0.07])" (p. 4, lines 148–150) — Since the confidence intervals for the parent-rated and self-rated effects overlap, I would avoid calling one "larger", as the difference is not statistically significant.

*Response: Thank you for this helpful point. We agree that, given the overlapping confidence intervals, the effects should not be described as "larger." We have revised the text to report the parent- and self-rated effects side by side without implying a significant difference. See except below:*

Queen Mary University of London
Department of Biological and Experimental Psychology
School of Biological and Behavioural Sciences
Mile End Road
London E1 4NS

*'The indirect (mediated) effects of education-specific non-cognitive skills at age 9 were statistically significant for both parent-rated (ß = 0.05 [95% CI, 0.02, 0.07]) and teacher-rated measures (ß = 0.13 [95% CI, 0.10, 0.15]' [Page 4 of the revised manuscript]*

**Methods**

9) More detail is needed on the measures. Either in the main text or Supplement, it would be helpful to provide consistent information across all measures on: who the rater was, number of items, response scale (e.g., 3-point Likert scale from 1, "Certainly True", to 3, "Not True"), whether items were reverse scored, and what high/low scores indicate (e.g., did higher scores indicate higher/lower perceived ability). This would help readers interpret the descriptive statistics in Supplementary Table 1 more easily. Including information on the reliability and validity of the measures would also strengthen this section.

*Response: We thank Dr. Austerberry for this helpful suggestion. We have revised the Methods to provide consistent information across all measures, including rater, number of items, response scale, scoring direction, and interpretation of higher scores. An example of the revised format is provided below:*

*'At age 16 education-specific non-cognitive skills were assessed via self-reports provided by the twins. The battery of education-specific non-cognitive constructs included the following measures: Academic self-concept: 10 items of a brief academic self-concept scale (adapted from 67, e.g., 'I am good at doing tests') rated on a 5-point Likert scale from 5 =  'Very much like me' to 1 = 'Not like me at all'. Items were reverse coded where necessary with higher scores represent greater academic self-concept.' [Page 16 of the revised manuscript]*

*Regarding the reliability of the measures, we did not have access to item-level data and therefore we were unable to estimate internal consistency and reliability directly. However, to address this, we have added information on the reliability of the scales from published data. We have added a paragraph on this to Supplementary Note 2, and we refer to this note in the main manuscript [Page 15].*

*Please see below the revised excerpt added to the Supplementary Note 2:*

*'Information on the reliability of the non-cognitive measures.*

*'Strengths and Difficulties Questionnaire (SDQ): The Cronbach's α for the SDQ has been reported to be .73 on average, with a mean cross-informant correlation = .34, and mean test–retest stability = .62 after 4–6 months (1).*

*School Engagement (Student Engagement Instrument; SEI: The Cronbach's α values of SEI ranging from .72 to .88 across subscales in the original validation study (2).*

**Queen Mary University of London**
Department of Biological and Experimental Psychology
School of Biological and Behavioural Sciences
Mile End Road
London E1 4NS

*Short Grit Scale (GRIT–S)*
*The Short Grit Scale (3) demonstrates internal consistency values typically between .73 and .83 across validation samples.*

*Curiosity and Exploration Inventory (CEI)*
*The Curiosity and Exploration Inventory (4) shows Cronbach's α values ranging approximately from .58 to .80 depending on the subscale (Exploration, Absorption) and sample.*

*PISA-derived indices (Mathematics self-efficacy, Mathematics interest, and Attitudes toward school)*
*According to OECD PISA Technical Reports, internal consistency reliabilities for these indices commonly range from α ≈ .70 to .90 across countries and assessment cycles (5).*

*Other TEDS-adapted measures:*
*Other TEDS-adapted scales—including Classroom Environment Questionnaire, Academic Self-Perceived Ability, Academic Interest, Academic Self-Concept, Academic Ambition, and the Literacy and Mathematics Environment questionnaires—were adapted from validated educational instruments. Item-level responses for these scales are not provided in the TEDS data release, precluding internal reliability estimation in the present analyses.' [Page 3 and 4 of the revised Supplementary Information].*

10) The Strengths and Difficulties Questionnaire (SDQ) tends to be described as a measure of emotional, behavioural, and relationship functioning, rather than self-regulation. While some researchers have used select SDQ items as indicators of dysregulation (e.g., Holtmann et al., 2011, https://doi.org/10.1159/000318164), the full measure is not typically considered a measure of self-regulation.

Did you have a pre-specified cutoff for acceptable model fit? Several of the CFAs showed poor model fit—particularly:
•General cognitive factor at age 9 (Supplementary Table 23),
•Self- and teacher-reported non-cognitive skills at age 9 (Supplementary Table 24),
•Self-reported non-cognitive skills at age 16 (Supplementary Table 24),
•Teacher- and self-rated SDQ scores at ages 9, 12, and 16 (Supplementary Table 25).
It would be worth investigating why model fit is poor in these cases and considering potential adjustments.

*Response: Thank you for these thoughtful observations. We agree that in the psychological literature the Strengths and Difficulties Questionnaire (SDQ) has been mostly described as a measure of general emotional, behavioural, and social functioning rather than a specific index of self-regulation. In the current study, we used the SDQ latent factor to represent a broad behavioural regulation construct, following prior work using the same dataset (Malanchini et al., 2024) and drawing*

*from research in the field of economics that has used the SDQ as a measure of non-cognitive skills capturing behavioural and emotional regulation (e.g., Conti & Heckman 2010; Attanasio et al., 2020). We have clarified this in the revised Methods sections. Please see excerpt below:*

*'The Strengths and Difficulties Questionnaire (SDQ) (78) was used to evaluate emotional and behavioural self-regulation across all ages, consistent with previous work using the same dataset (26) and aligned with the economics literature (39,53).' [Page 17 of the revised manuscript]*

*Regarding model fit, we used conventional criteria to evaluate CFA models (CFI ≥ .90, RMSEA ≤ .08, SRMR ≤ .08) but note that a few latent models—particularly at younger ages—showed poorer fit (Supplementary Tables 25–26). This likely reflects developmental variability and limited item coverage. Our choice of combining all the available education-related non-cognitive measures at each age was driven by our previous work in this area (Malanchini et al., 2024). We showed that these latent factors capture individual differences in non-cognitive skills more reliably than individual non-cognitive measures which are known to be low in reliability and subject to great response heterogeneity (Morris et al., 2021). However, due to this limitation, which we discuss in greater depth in the revised draft (see Discussion section and excerpt below), we performed our analyses not only on the latent factors, but also on the individual non-cognitive measures.*

*'Third, to improve measurement reliability and reduce error, we employed latent factor models that captured shared variance across conceptually related constructs. This strategy was based on prior validation using the same dataset (26), where latent composites showed stronger predictive utility than individual non-cognitive indicators—many of which are known to exhibit low reliability and substantial response heterogeneity (52). However, we also acknowledge that model fit varied across timepoints, with relatively poor fit observed for several models, particularly at younger ages (e.g., age 9 self- and parent-reported constructs; see Supplementary Tables 25–26). These discrepancies likely reflect both developmental variability and limited item coverage (53). As such, we reported results based on both latent factors and individual indicators (see Figure 1 and 2).'[Page 13 of the revised manuscript].*

11) I was unsure whether age and sex were correctly specified in your CFAs. In Supplementary Tables 24 and 25, it appears that they were included as indicators of the latent factors. If the intent was to control for age and sex, they should be entered as exogenous predictors of the latent variables rather than endogenous indicators.

*Response: Sex and age were not included as indicators in the models. Instead, all variables were adjusted for sex and age prior to analysis, ensuring that the associations reported are independent of these socio-demographic effects. We have now added a foot note in the revised Supplementary Table 25 and 26 to clarify this.*

Queen Mary University of London
Department of Biological and Experimental Psychology
School of Biological and Behavioural Sciences
Mile End Road
London E1 4NS

How many bootstrap samples were used in the mediation models? I am assuming from your code that you used the lavvan default of 1000 but it would be helpful to specify.

*Response: We thank the reviewer for pointing this out. Yes, we used the default bootstrap setting in the lavaan package, which employs 1,000 bootstrap samples. We have now explicitly stated this in the manuscript for clarity, see excerpt below:*

*'We used the default of 1,000 bootstrap samples in lavaan to estimate standard errors and confidence intervals for indirect effects' [Page 20 of the revised manuscript]*

12) I could not find mention of how you handled missing data. In the supplementary mediation results, it looks like you used listwise deletion, resulting in substantial data loss. However, in your code, it looks as though you used the lavaan default—which I think is FIML (which is usually the preferable approach for reducing the risk of bias and power loss). Please could you add to the Methods details on whichever approach you took.

*Response: We thank the reviewer for this helpful observation. We confirm that we used Full Information Maximum Likelihood (FIML), the default in lavaan when fitting models to raw data. The apparent data loss reflects differences in missingness across the specific variables included in each model. We have clarified this in the Methods section, see excerpt below:*

*'We used Full Information Maximum Likelihood (FIML) to account for missing data.' [Page 19 of the revised manuscript]*

With these revisions, I believe the manuscript will make a strong and impactful contribution.

Reviewer #2 (Remarks to the Author):

This study explores whether so called non-cognitive skill may act as mediators between polygenic risk score and academic achievement in TEDS sample. In general the paper is well-written and the authors are obviously experts of the genetic methodology and sample they use.

*Response: We thank the reviewer for their positive evaluation of our work.*

1) I am not an expert of studies with DNA methods and cannot evaluate
the decisions made regarding the polygenic risk scores. My evaluation focuses more on the conceptual issues. I have two main issues with what is done in the manuscript and how the results are interpreted: Both are related to the measures;
The study outcome is academic performance evaluated by teachers at ages 7

**Queen Mary University of London**
Department of Biological and Experimental Psychology
School of Biological and Behavioural Sciences
Mile End Road
London E1 4NS

(English, mathematics), 9, and 12 (English, mathematics, and science). At age 16, mean grades for English, mathematics, and science was used.

*Response: Thank you for this comment. We agree it is essential to be precise in describing the assessments across developmental stages. To clarify: at age 7, teacher assessments were available only for English and mathematics, as science was not part of the Key Stage (KS)1 assessment. Science was included at ages 9 (KS2), 12 (KS3), and 16 (KS4), when these data were available.*

*To examine whether the results changed when we considered a more consistent measure of academic achievement across ages, we conducted a number of sensitivity analyses. First, we considered a two-subject (English and mathematics) composite measure at ages 9 and 12, comparable to the measure we have for age 7. Second, we conducted separate analyses for each subject. The results were highly consistent across these analyses and are presented in Supplementary Table 23 of the revised manuscript. We have also revised the Methods section to clarify this, as shown below:*

*'Consistent with the standardised assessment at Key Stage 1 (KS1), at age 7 academic achievement was measured as a composite of standardised performance in English and mathematics. At age 9 and 12, and consistent with the standardised assessment at KS2 and KS3, achievement was measured as a composite score of performance across three subjects: English, mathematics, and science. To ensure comparability across ages, we conducted additional sensitivity analyses using a composite score including only English and mathematics at ages 7, 9 and 12, as well as analyses examining individual subjects separately (Supplementary Table 7).' [Page 14 and 15 of the revised manuscript].*

*We also added a line in Results section, see excerpt below:*

*'Sensitivity analyses considering a composite score including achievement in English and mathematics at ages 7, 9, and 12 yielded a similar pattern of results. The results were also highly consistent when we considered each subject separately (Supplementary Table 7).' [Page 5 of the revised manuscript].*

2) The so called non-cognitive skills included:
-at age 9: academic self-perceived ability , academic interest and the Classroom Environment Questionnaire (satisfaction with classroom; enjoyment of learning, educational opportunities)
- at age 12: academic interest, academic self-perceived ability, the literacy environment questionnaire and the mathematics environment questionnaire
-at age 16: academic self-concept, school engagement, grit, academic ambition, time spent on math, math self-efficacy, math interest, curiosity, and attitudes toward school

**Queen Mary University of London**
Department of Biological and Experimental Psychology
School of Biological and Behavioural Sciences
Mile End Road
London E1 4NS

Cognitive ability included mainly language and visuo-spatial tasks from WISC. Raven, etc.
First, the non-cognitive skills are not skills, and some are not even characteristics of the child/adolescent. Satisfaction with classroom may not have anything to do with the child, for example. Sometimes teachers and classrooms are indeed not that great and it has nothing to do with the skills of the student in the "non-cognitive skill "domain.

*Response: We thank the reviewer for this important observation. We fully agree that many constructs commonly grouped under the term "non-cognitive skills" are not strictly "skills" in the narrow sense, and that some, such as perceptions of classroom environment, partly reflect contextual experiences as well as characteristics of the child. The term 'noncognitive skills' was originally coined by economists studying individuals who were equivalent in cognitive ability but differed in educational attainment (Heckman et al., 2006), and we adopted an approach to non-cognitive skills that mirrored this original definition, so 'all contributions to educational attainment that are not cognitive skills.' This is also consistent with our research laboratory's previous work in this area (Malanchini et al., 2024).*

*Non-cognitive skills have been defined in many conflicting ways, and the field lacks a clear understanding of what characteristics make up this big umbrella. However, we acknowledge that the label encompasses a heterogeneous set of constructs—including motivational beliefs, attitudes, behavioural dispositions, and some contextually embedded perceptions—which is a limitation recognized in recent reviews (e.g., Smithers et al., 2018). Most prior frameworks (e.g., Richardson et al., 2012; Schneider, 2017) focus on individual characteristics, and do not typically include contextual perceptions such as classroom satisfaction. We have revised the Discussion to clarify this point, and we emphasize that part of our motivation was precisely to empirically evaluate which of these constructs are most relevant as mediators of genetic associations with achievement, and to inform future efforts toward a clearer taxonomy of education-related non-cognitive characteristics, See excerpt below:*

*'A further limitation concerns the heterogeneity of the constructs grouped under the umbrella of non-cognitive skills. While this label has been widely used in both the psychological and economic literature, it encompasses a widely diverse set of characteristics, ranging from motivational beliefs and self-regulatory strategies to attitudes and perceptions of the learning environment, that differ in their specificity, developmental stability, and contextual embedding (24,34). For instance, classroom satisfaction may reflect environmental quality as well as a dispositional characteristic. We retained the term 'non-cognitive skills' for consistency with prior work and to facilitate comparison with the extant literature (24,26,34), but we acknowledge its limitations. In fact, one of our goals was to empirically examine which of these constructs serve as meaningful mediators of the polygenic score prediction of academic achievement, to inform future efforts toward a clearer taxonomy of education-related non-cognitive skills.' [Page 13 of the revised manuscript]*

Queen Mary University of London
Department of Biological and Experimental Psychology
School of Biological and Behavioural Sciences
Mile End Road
London E1 4NS

3) Secondly, and more importantly, this study does not include assessments of the actual reading, mathematics, or science skills. The cognitive skills included are known to be surprisingly weak correlates of reading and math skills and difficulties. Therefore, including them does not help with covarying the cognitive or skill variance in the equations. At the same time, the so-called non-cognitive measures are known to be strongly correlated with the skills and the basic academic skills are correlated also with the outcome. Therefore, the found associations/mediation results may be confounded by the student's skills in reading and math. Particularly the included motivation-measures and self-reported skills (self-concept of ability and self-efficacy -type measures) share much variance with the basic academic skills. Without including assessments of the skills, these results should not be interpreted as evidence for non-cognitive mediation.

The problem is also to be seen in the interpretations/conclusions; authors conclude that "This might be particularly useful knowledge for interventions aimed at fostering learning. Interventions aimed at enhancing students' academic self-concept, interests, and motivations, such as inquiry-based learning42, positive feedback43, and goal-setting44, might be particularly effective." First of all, this conclusion is very far from what was examined. Also, based on the available evidence in literature I'm not convinced yet if we should indeed focus on these targets when children's skills are not in place. Tis is also what these results can conclude. For example, studies examining the associations between reading and math skills and self-concept of ability development suggest that skills predict self-concept appraisals and not the other way around. Same is true for interest in reading. This suggest that motivation and self-beliefs follow skills and if the main target of education is skilled people, then we should rather focus on skills that (false) self-beliefs.

*Response: We thank the reviewer for raising this important concern regarding the absence of direct assessments of academic abilities (e.g., reading ability, mathematics ability) rather than achievement across all timepoints. We agree that these abilities may share variance with some of the non-cognitive constructs included in our mediation models—particularly self-concept, self-efficacy, and motivational traits—and that this overlap could complicate causal interpretation. Standardized test-based assessments of reading and mathematics are available in the TEDS dataset only at age 12, limiting their utility in a developmental mediation framework spanning multiple ages. However, previous work in the TEDS sample shows that academic achievement scores are strongly correlated with test-based assessments of reading (Rimfeld et al., 2019) and mathematics (with correlations between test-based assessments and GCSE scores ranging between .66 and .75, Malanchini et al., 2020). Previous work has demonstrated that teacher-rated academic achievement—the main outcome variable in our study— is substantially correlated with standardized test scores at all key stages (r ranging from 0.71 to .079 for English, from 0.64 and 0.74 for mathematics, and from 0.65 and 0.69 for science, Rimfeld et al.., 2019), supporting its use as a valid proxy for academic performance.*

Queen Mary University of London
Department of Biological and Experimental Psychology
School of Biological and Behavioural Sciences
Mile End Road
London E1 4NS

*Furthermore, we mitigated this potential limitation by controlling for general cognitive ability (g). First, our results were robust to adjusting for g, a measure constructed form two verbal and two non-verbal tests, at each timepoint (Supplementary Table S3). These g measures have been found to correlate strongly with test-based academic abilities such as literacy and numeracy (Ritchie et al., 2014). We expand upon this potential limitation in the revised Discussion, see excerpt below:*

*'Finally, academic achievement was measured using teacher ratings rather than standardized test scores. However, prior work in TEDS has demonstrated that these ratings provide reliable estimates of academic performance and correlate strongly with standardized assessments across compulsory education (61). While we did not consider standardized test-based measures of academic abilities (e.g., numeracy or reading accuracy) in the current study due to their limited utility for our developmental mediation framework, previous work in the TEDS sample has shown that teacher-rated and exam scores are strongly correlated with test-based abilities (62). Furthermore, our results were highly consistent after we statistically controlled for general cognitive ability (g), measures as a composite of verbal and non-verbal reasoning tests, which are strongly associated with academic outcomes (63,64).' [Page 13 and 14 of the revised manuscript]*

*We have also revised the concluding sentence, which we agree extended beyond the evidence provided in the current study, and we have highlighted the removed sentence. [Page 12 of the revised manuscript]*

*'This might be particularly useful knowledge for interventions aimed at fostering learning. Interventions aimed at enhancing students' academic self-concept, interests, and motivations, such as inquiry-based learning (45), positive feedback (46), and goal-setting (47), might be particularly effective.'[removed sentence in page 12 of the original manuscript]*

Reviewer #3 (Remarks to the Author):

This manuscript is exceptional in many ways. It reports findings from very sophisticated analyses addressing extremely important and well-conceived questions. The findings could inform future interventions to improve educational outcomes within the context of gene-environment correlations. Moreover, the important issues and findings are described in one of the best-written manuscripts I have read in decades.

*Response: We are grateful to the reviewer for this positive evaluation of our work.*

1) The manuscript misrepresents the representativeness of the sample. The sample was not selected to be representative of any population and one cannot claim that

dizygotic twins are representative of the general population. That statement needs to be amended.

*Response: Thank for pointing this out. We agree that, although the families invited to take part in TEDS were representative of the broader population of England and Wales for socio-economic status and ethnicity for their birth cohort, twins are a selected population. This is particularly consequential when studying early social development. We have toned down claims of representativeness and discuss this issue in the revised manuscript (see excerpt below).*

*'…Furthermore, dizygotic twins may not accurately represent singleton populations. Although recent large-scale studies (e.g., Christensen et al., 2006) report negligible mean differences in academic achievement between twins and singletons, caution is still warranted when generalising from a twin sample.'[Page 14 of the revised manuscript]*

2) The use of latent variables derived from multiple variables obtained from multiple informants (that differed by wave) is mathematically defensible, but the manuscript needs to warn readers that this bird's eye view of the potential role of non-academic mediators of genetic propensities could be misleading. Readers need to understand that the findings do not tell us if any single variable from a given informant is related to academic achievement. This is extremely important in helping readers translate findings into interventions. Far more detailed analyses would be needed to know which components of latent variables, if any, might be good targets for interventions.

*Response: Thank you for this observation. We agree that latent composites may obscure the contributions of specific variables or informants. We now explicitly note this limitation in the manuscript and clarify that we complemented the results obtained using latent variable models with analyses that examined individual non-cognitive measures to provide a more nuanced perspective. Please see the excerpt below:*

*'Beyond examining the role of non-cognitive skills measured as latent factors, therefore capturing shared variance across different aspects of education-specific characteristics and self-regulation, we considered individual non-cognitive measures. We conducted a set of fine-grained analyses to identify which aspects of non-cognitive skills mediated genetic effects on academic achievement across development. This offered a more detailed view of construct-specific associations and reduced the risk of overinterpreting the results emerging from broad latent dimensions. First, we observed that specific student-centred academic attitudes and perceptions, such as academic interest, curiosity, and perceived ability, evinced stronger mediation effects as compared to perceptions of the learning environment that were external to each individual, such as perception of the classroom or the learning environments. These findings align with previous work, which emphasised the critical role of intrinsic, student-centred non-cognitive skills (e.g., intellectual curiosity and academic self-concept) in gene-environment transactions (40,44)*

Queen Mary University of London
**Queen Mary University of London**
Department of Biological and Experimental Psychology
School of Biological and Behavioural Sciences
Mile End Road
London E1 4NS

*driving students to actively engage in academic settings beyond what environmental factors alone can achieve (45).' [Page 11 of the revised manuscript]*

3) I am not at all convinced that a latent variable derived from the variables scales of the Strengths and Difficulties Questionnaire provides a meaningful measure of "self-regulation." This might explain why that construct was not found to be a strong mediator. The factor analysis creates a single latent score based on several psychopathology scales and adaptive prosocial personality. I wonder if the results would have been different had prosociality not been included and a bifactor model had been used to distinguish general and specific factors. I would like to see the analyses of the SDQ addressed in more detail, perhaps in a separate paper. I would be very surprised if some aspects of maladaptive attention, emotion, and conduct did not mediate the predictions based on the EA PGS.

*Response: We thank the reviewer for this comment that was also raised by Reviewer 1 (see point 10 of Reviewer 1's comments). We agree that the SDQ primarily captures broad behavioural and emotional adjustment rather than a pure measure of self-regulation. In our analyses, we used the latent SDQ factor to represent a general behavioural regulation construct, following prior research adopting similar approaches (e.g., Conti and Heckman, 2010; Attanasio et al., 2020). The factor therefore reflects shared variance across the SDQ subscales, including both adaptive (prosocial) and maladaptive dimensions, rather than isolating specific regulatory components.*

*We acknowledge that alternative models—such as excluding the prosocial scale or applying a bifactor approach—could yield different insights into the distinct contributions of attention, conduct, or emotional regulation. However, these exploratory psychometric analyses are beyond the scope of the current paper, which focuses on consistent modelling across multiple non-cognitive domains. We have clarified this rationale and limitation in the revised Discussion. Please see excerpt below:*

*'The SDQ was used as a latent factor capturing broad behavioural and emotional adjustment—consistent with its treatment in prior economics-based research (39,53), rather than a narrow self-regulation measure. Future work might explore alternative structures (e.g., bifactor models) and develop developmentally sensitive, structurally valid measures of non-cognitive characteristics that allow for greater cross-age and cross-context comparability.' [Page 13 of the revised manuscript]*

Reviewer #4 (Remarks to the Author):

1) The study by Zhou et al. examines how non-cognitive skills (as reported by children, parents, and/or teachers) such as self-regulation, learning attitudes and strategies mediate the associations between polygenic indexes for cognitive ability,

**Queen Mary University of London**
Department of Biological and Experimental Psychology
School of Biological and Behavioural Sciences
Mile End Road
London E1 4NS

non-cognitive traits and educational attainment, and subsequent academic achievement in English, maths, and science at ages 7, 9, 12, and 16. The central questioned being tackled is an important one – through what pathways and processes does genetic liability unfold across development to influence academic outcomes? By utilising the twin structure of the cohort the study is also able to control for shared family environment, population stratification and assortative mating, which is a noted strength, as are the multiple informants. While the practical implications are likely some way off, this work generates testable hypotheses about which interventions - and who they should target, and when - might moderate genetic risk to poorer educational outcomes. From my perspective, these findings also underscore the need to account for genetic confounding in measures of student skills and behaviours; teacher-reported measures are free from passive gene–environment correlation, which might make them especially valuable for examining indirect paths (?).

While we find the overall methodological approach appropriate —and note the use of a well-characterised, large longitudinal twin cohort and pre-registration of analyses—the analytical strategy and variable choices are poorly justified, and the relationship of these findings to the group's prior work is not clearly articulated. Our major comments focus on the following:

**Clarify overlap with previous published research**
The authors cite two prior papers from their group (refs 29 and 17) but do not clearly explain how this study differs from or extends them. From a skim, much of the current manuscript appears to replicate results reported in Malanchini et al. (2024), using the same cohort, timepoints, and overall design to test very similar hypotheses—only without a mediation analysis? This overlap should be explicitly acknowledged, with a clear statement of what is novel here.

e.g. lines 329-331-replicating results from ref 29- but not cited, framed as novel finding "the mediating role of education-specific non-cognitive skills increased developmentally, pointing to the growing importance of students' perceived non-cognitive profiles and experiences in their academic journeys."
vs

Malanchini et al. (2024) discussion: "independent genetic contribution of non-cognitive skills to academic achievement increased developmentally"

*Response: We thank the reviewer for highlighting the need to clarify how this study relates to our previous work. We have now added a statement in the manuscript to explicitly acknowledge how this study extends our previous work (Malanchini et al., 2024) and focuses on addressing a different research question. Malanchini et al., 2024 aimed to investigate the developmental change and stability in genetic effects associated with non-cognitive skills related to education. Their findings show how genetic associations between non-cognitive skills and academic achievement increase across development, which the authors interpret as consistent*

**Queen Mary University of London**
Department of Biological and Experimental Psychology
School of Biological and Behavioural Sciences
Mile End Road
London E1 4NS

*with the possibility of gene-environment correlation processes –i.e., genetic effects amplified by correlated environmental experiences that accumulate across development. The current study builds on this initial evidence and investigates what are the psychological characteristics that help translating genetic dispositions into observed academic achievement across development, therefore actively testing the previously hypothesised gene-environment correlation pathways. We clarify how this study builds on our previous work but asks a fundamentally different research question in the revised manuscript.*

*'Our cross-sectional analyses showed that the latent dimensions of education-specific non-cognitive skills and self-regulation both mediated the associations between education-related genetic disposition and academic performance. However, while the role of self-regulation was stable across compulsory education, the mediating role of education-specific non-cognitive skills increased developmentally, pointing to the growing importance of students' perceived non-cognitive profiles and experiences in their academic journeys. This developmental increase is consistent with the possibility that, as they grow up, children become more aware of their aptitudes and appetites towards learning. As they gain greater self-awareness and autonomy, students might become increasingly more able to shape their environmental contexts in ways that allow them to cultivate these non-cognitive skills and, in turn, foster their academic performance (40).' [Page 11 of the revised manuscript]*

2) Relatedly, the manuscript cites Zhou et al. (2024; ref. 17), which seems to employ the same cohort, PGIs, and outcome measures (?) but focuses on family-environment mediators rather than non-cognitive skills. The authors should explicitly state how the present study's findings converge with—or diverge from—those reported by Zhou et al. Drawing these connections will help readers understand how the current study builds on the existing research and will help clarify its unique contribution.

*Response: We thank the reviewer for highlighting the need to clarify how this study relates to our prior work. We have now revised our introduction, please see excerpt below:*

*'The present study extends this line of enquiry and investigates whether, beyond family-related experiences (17), another important set of psychological characteristics –non-cognitive skills– mediate education-associated genetic effects on academic achievement across development.' [Page 2 of the revised manuscript]*

3) **Choice of PGIs**
It isn't made clear why it is important to look at EA PGI alongside the cog and non-cog PGIs (given the EA PGI is so powerful, what does adding the cognitive PGI tell you?), or how the five PGIs relate (or not) to one another. For example, should the EA and cognitive PGIs be more highly correlated (and show more similar patterns of

**Queen Mary University of London**
Department of Biological and Experimental Psychology
School of Biological and Behavioural Sciences
Mile End Road
London E1 4NS

association) than the non-cognitive PGIs? In what ways does the Demange et al. (ref 31) versus Malanchini et al (ref 29) 'non-cognitive' PGIs differ from each other? Should the reader expect overlapping patterns of associations?
A brief description of why each of the five PGIs were selected, alongside how each PGI was derived—and a correlation matrix showing the inter-relations among the five PGIs—would help the reader interpret the observed patterns of association in the manuscript.

*Response: We thank the reviewer for the comment. We have clarified that the primary analyses focus on three polygenic scores—educational attainment (EA, Lee et al., 2018)), cognitive performance, and non-cognitive skills—derived from Malanchini et al. (2024). Genetic effects associated with non-cognitive skills were derived by separating genetic variants associated with both education and cognitive performance from those unique to educational attainment beyond cognitive skills. This extension of the original GWAS-by-subtraction model (Demange et al., 2021) also includes SES-related traits (i.e., income and deprivation) in the non-cognitive factor (Malanchini et al., 2024). Including these three genetic instruments allowed to decompose education-associated genetics into a cognitive and a non-cognitive component and to examine whether these genetic effects operate via similar experiential pathways. We have clarified our rationale in the revised Manuscript, please see excerpt below:*

*'We considered genetic effects associated with educational attainment (EA) (14), as well as those associated with cognitive and non-cognitive skills (26). This allowed us to examine putatively different pathways from genetic disposition to academic achievement.' [Page 3 of the revised manuscript]*

4) **Choice of mediator variables**
An explanation of inclusion criteria for behavioural dimensions included in the mediator analysis is missing. Was there a specific rationale for the inclusion/exclusion of certain measures, and how were they systematically rated as cognitive vs non-cognitive, and beyond this, education-specific vs non-education specific? For example, if you ask students how good they are at school, their answers will track with how well they actually do – so what are the conceptual and/or data-driven distinctions being made between cognitive and non-cognitive skills?

*Response:*

*We thank the reviewer for highlighting this important point that aligns with point 2 raised by Reviewer 2. We agree that the boundaries between cognitive and non-cognitive constructs is not always clear, particularly for self-evaluative measures such as academic self-concept and self-efficacy, which tend to show modest correlations with performance in cognitive tests (Furnham and Cheng, 2024). In the current study, we considered 'non-cognitive' those measures of behaviours, attitudes and motivations that have been found to associate with academic outcomes beyond performance in cognitive tests (Richardson et al., 2012; Malanchini et al., 2019;*

**Queen Mary University of London**
Department of Biological and Experimental Psychology
School of Biological and Behavioural Sciences
Mile End Road
London E1 4NS

*Conti and Heckman, 2010). However, because we acknowledge that these are not fully independent of cognitive performance, we conducted sensitivity analyses controlling for general cognitive ability (Supplementary Table 22). We found that, although some of the effect sizes were attenuated, the results were overall consistent. We clarify this in the revised manuscript, see excerpt below.*

*'In the current study, we considered 'non-cognitive' those measures of behaviours, attitudes and motivations that have been found to associate with academic outcomes beyond performance in cognitive tests (26,34,39). However, because previous research has shown that students' non-cognitive skills correlate with students' cognitive ability (26,28), we tested the possibility that cognitive ability could account for the observed mediation effects.' [Page 10]*

*We have also revised Discussion section to clarify this in greater detail, please see excerpt below:*

*'A further limitation concerns the heterogeneity of the constructs grouped under the umbrella of non-cognitive skills. While this label has been widely used in both the psychological and economic literature, it encompasses a widely diverse set of characteristics, ranging from motivational beliefs and self-regulatory strategies to attitudes and perceptions of the learning environment, that differ in their specificity, developmental stability, and contextual embedding (24,34). For instance, classroom satisfaction may reflect environmental quality rather than a stable dispositional characteristic. We retained the term 'non-cognitive skills' for consistency with prior work and to facilitate comparison with the extant literature (24,26,34), but we acknowledge its limitations. In fact, one of our goals was to empirically examine which of these constructs serve as meaningful mediators of the polygenic score prediction of academic achievement, to inform future efforts toward a clearer taxonomy of education-related non-cognitive skills.' [Page 13 of the revised manuscript]*

5) **Latent factors vs. individual measures**
The manuscript would benefit from a clearer justification and more consistent reporting regarding the use of both latent-factor composites and individual non-cognitive measures. For instance, why build latent factors and also analyse individual scores? What distinct insights are gained, or which set of results should the reader focus on? We note that Supplementary Table 24 shows that some latent models (e.g. child- and parent-reported non-cognitive skills at age 9) fit poorly, and some do not include all individual measures (age 16). The model fits should be reported so readers understand the strength—and limitations—of the latent constructs before interpreting mediation results. Finally, there seems to be inconsistency across analyses with only education specific NCS mediation reported

**Queen Mary University of London**
Department of Biological and Experimental Psychology
School of Biological and Behavioural Sciences
Mile End Road
London E1 4NS

for individual measures, but not self-regulation?

*Response:*

*We thank the reviewer for this helpful and constructive comment. In line with what suggested also by Reviewer 3, we have revised the Methods and Discussion sections accordingly:*

*Discussion:*

*'Beyond examining the role of non-cognitive skills measured as latent factors capturing shared variance across different aspects of education-specific characteristics and self-regulation, we considered individual non-cognitive measures. We conducted a set of fine-grained analyses to identify which aspects of non-cognitive skills mediated genetic effects on academic achievement across development. This offered a more detailed view of construct-specific associations and reduced the risk of overinterpreting the results emerging from broad latent dimensions.' [Page 11 of the revised manuscript]*

*'Third, to improve measurement reliability and reduce error, we employed latent factor models that captured shared variance across conceptually related constructs. This strategy was based on prior validation using the same dataset (26), where latent composites showed stronger predictive utility than individual non-cognitive indicators—many of which are known to exhibit low reliability and substantial response heterogeneity (52). However, we also acknowledge that model fit varied across timepoints, with relatively poor fit observed for several models, particularly at younger ages (e.g., age 9 self- and parent-reported constructs; see Supplementary Tables 25–26). These discrepancies likely reflect both developmental variability and limited item coverage. For transparency and robustness, we therefore reported results based on both latent factors and individual indicators (see Figure 1 and 2). The SDQ was used as a latent factor capturing broad behavioural and emotional adjustment—consistent with its treatment in prior economics-based research (39,53), rather than a narrow self-regulation measure. Future work could explore alternative structures (e.g., bifactor models) and develop developmentally sensitive, structurally valid measures of non-cognitive characteristics that allow for greater cross-age and cross-context comparability.' [Page 13 of the revised manuscript]*

*See excerpt below:*

*Supplementary Note 2:*

*'We constructed latent factors of education-specific non-cognitive skills at ages 9, 12, and 16, separately for different raters where available. Structural validity was*

*assessed using confirmatory factor analysis with one twin per pair to account for non-independence. Model fit was evaluated using standard indices (CFI, TLI, RMSEA, SRMR), and loadings for all indicators are presented in Supplementary Table 25. While teacher- and self-reported models at ages 9 and 12 demonstrated acceptable to good fit, the child- and parent-report models at age 9 and self-report model at age 16 yielded modest fit indices, indicating limitations in shared structure across indicators. As such, both latent and individual indicator analyses are presented in the main text to enable interpretation across both aggregated and item-level resolution. For self-regulation, we used latent factors derived from the Strengths and Difficulties Questionnaire (SDQ) rather than individual subscales. This decision was based on the limited conceptual specificity of individual SDQ subscales for modelling self-regulatory processes in the academic domain, as well as the small and diffuse nature of item-level effects observed across waves and reporters.' [Page 3 of the revised Supplementary Material]*

Minor comments

6) Abstract
The abstract's focus on mediation at age 9 appears a little cherry-picked (since effects aren't consistent across reporters at age 9?) and overlooks how small the overall PGI–achievement associations can be (e.g., ~0.10 for teacher-rated achievement at age 9 with the non-cognitive PGI). To avoid overstating the findings, please report the absolute size of the indirect (mediated) effect or the percentage of total variance it explains—so readers can see that, despite reported proportions, the effects themselves are modest.

*Response: We thank the reviewer for this helpful comment. We agree with the concern and have revised the Abstract to reduce the emphasis on age 9 and to provide a fuller picture of the mediation effects across constructs and developmental stages. Specifically, we now include the lower bound of the mediation effects as well as the corresponding absolute effect sizes, alongside the reported percentages, to present the findings in a more transparent and balanced manner. See excerpt below:*

*'We found that non-cognitive skills mediated between less than 5 and up to 64% of the genetic prediction of academic achievement and that effects were larger and more robust for motivation and attitudes (ß ≈ 0.13) than for self-regulation (ß ≈ 0.01–0.03). This pattern of results held longitudinally and was replicated in within-family analyses, where non-cognitive skills accounted for up to 83% of the total mediation effects. This highlights the contribution of non-cognitive skills beyond shared familial factors, likely reflecting how children evoke and select experiences that align with their genetic propensity and lead to differences in academic development.' [Page 1 of the revised manuscript]*

7) Introduction

Queen Mary University of London
**Queen Mary University of London**
Department of Biological and Experimental Psychology
School of Biological and Behavioural Sciences
Mile End Road
London E1 4NS

As noted, the introduction requires more contextualisation for the study and clearer motivation for the section of variables and analytical strategy. Some of this appears in the Discussion (e.g. lines 366–370, 402–404); it should be introduced and expanded in the Introduction.

*Response: We thank the reviewer for this constructive and helpful comment. We now revised the corresponding paragraph in Introduction, please see excerpt below:*

*‘To address these questions, we employed a design with unique features, including the measurement of several non-cognitive skills across development, such as academic motivation, academic interest, and self-regulation at age 7, 9 ,12 and 16 (see Methods). These constructs were selected based on prior theoretical and empirical work that identified them as characteristics that predict academic achievement and engagement beyond cognitive performance (24,34). Data on non-cognitive skills were obtained from multiple raters, including children, parents, and teachers. We considered genetic effects associated with educational attainment (EA)(14), as well as those associated with cognitive and non-cognitive skills (26). This allowed us to examine putatively different pathways from genetic disposition to academic achievement. We used a sibling difference design to partly adjust for shared sibling processes, such as passive gene-environment correlation (17,35), demographic between-family factors, population stratification (36), and assortative mating (37). These preregistered analyses (https://osf.io/vmpf7/) provide a detailed exploration of how non-cognitive skills contribute to translating genetic disposition into observed differences between children in academic achievement across compulsory education.’ [Page 3 of the revised manuscript]*

The manuscript cites many papers based on the cohort examined in the current study. To strengthen your argument(s), please incorporate findings from other cohorts beyond TEDS where possible.

*Response: To strengthen the generalizability of our arguments beyond the TEDS cohort, we have now incorporated findings from other cohorts that used different designs, for example adoption studies [e.g., page 2 or the revised manuscript].*

8) The key question 3 (lines 91–92 – ‘importantly’) inadvertently downplays your first two questions. I suggest repositioning this question higher, with more context as to why this is a salient question to ask.

*Response: Thank you for this suggestion, we now revised the corresponding sentence, please see excerpt below:*

*‘Third, do non-cognitive skills mediate polygenic score-academic achievement associations overtime, reflecting longitudinal mediation processes rather than co-occurrent associations?’ [Page 3 of the revised manuscript]*

Queen Mary University of London
Department of Biological and Experimental Psychology
School of Biological and Behavioural Sciences
Mile End Road
London E1 4NS

9) Results
The manuscript utilises non-cognitive skills data obtained from multiple raters, including teacher, parents, and the children themselves (line 103). Could they be dealt with systematically rather than interleaving them, for clarity? That way readers can compare across informants and more easily note where there are gaps.
In a similar vein, the ordering and orientation of figures (e.g., Figure 1 vs. Figure 2 vs Fig 3) are inconsistent, which hampers cross-comparison. Please present mediation figures in the same layout.

*Response: Thank you for this helpful suggestion. In the main text, we chose to organize the results by developmental stage rather than by informant to maintain a coherent narrative that follows age-related changes in non-cognitive mediation. While this required interleaving results from different raters, we have now clarified the source of each estimate more consistently throughout the Results section to improve clarity.*

10) Lines 111-118: as mentioned, given the focus of the manuscript, greater transparency is needed about how the CFA was selected, model fits should be stated and if any measures were excluded and why.

*Response: Thank you for this observation, we have added a more detailed explanation to the revised Results section, please see excerpt below:*

*'Creating latent dimensions of non-cognitive skills*
*Different measures of non-cognitive skills were collected at different ages, with ratings provided by children, parents, and teachers. Following our previous work (26), two latent dimensions of non-cognitive skills were created using confirmatory factor analyses: education-specific non-cognitive skills (including attitudes towards school, self-perceived academic ability, academic interest and curiosity) and self-regulation (encompassing behavioural and emotional regulation). A detailed description is provided in the Methods. Model fit indices are reported in Supplementary Tables 25–26. While model fit was acceptable to good for most raters and timepoints, self- and parent-reported models at age 9 showed poorer fit, and results for these should therefore be interpreted with caution.' [Page 3 and 4 of the revised manuscript]*

11) Lines: 123-126. I found this generally unclear – I initially read this as three PGIs, but its actually 5…?
Line 135: Now only three PGIs are reported? I am unsure why.
Line 162: ok, so now the results for Demange et al PGIs are being reported separately, but in a different format (tables 5 and 6). Presenting these data in the same format as Figure 1 would facilitate comparison of results across PGIs.

Queen Mary University of London
Department of Biological and Experimental Psychology
School of Biological and Behavioural Sciences
Mile End Road
London E1 4NS

*Response: We thank the reviewer for pointing this out. To avoid confusion, we have now removed the two earlier PGSs (Demange et al., 2021), which were previously included in the correlation matrix as secondary references. We now focus exclusively on the three more recent PGIs from Malanchini et al. (2024), which form the basis of our primary analyses.*

12) Line 130: This line states that all correlations between PGIs and achievement were statistically significant, yet the p-values are not reported in Supp Fig 1. Some of the reported correlations are near zero … (e.g. -0.01)?
Line 132: The stated range is wrong? (it's 0.04-0.22)
Line 134: why isn't the range also presented here for consistency?
Line 140: Please check if p-values really are exactly zero (across all supp tables)
Line 149: ..'increased across compulsory education within reporter would arguably be a more accurate way to describe the pattern of results in Fig 1? (i.e., indirect effects at age 9 for teacher reported achievement are ~x2 that at age 16)
Line 160: As noted, in my earlier comment that the total effects are sometimes small..

*We thank the reviewer for noting these. We have corrected the reported range for the NonCog PGS, which now reads r = .09 –.22 (with the minimum observed at age 9). In addition, to improve accuracy and transparency, we have replaced the previous Supplementary Figure with Supplementary Table 2a, which reports the exact correlation coefficients, sample sizes, and p-values. This ensures consistency between the values reported in the text and the supplementary materials. The Results text has been updated accordingly, see excerpt below:*

*'Correlations between polygenic scores and academic achievement at all ages were statistically significant and ranged from small to moderate (Supplementary Table 2a). Their magnitude tended to increase across development, particularly for the EA polygenic score (r ranging from 0.23 at age 7 to 0.35 at age 16) and the NonCog polygenic score (r ranging from 0.11 at age 7 to 0.24 at age 16). The correlations between polygenic scores and latent dimensions of non-cognitive skills were modest and statistically significant, except for self-rated non-cognitive skills at age 9 (see Supplementary Figure 2; Supplementary Table 2b). The correlations between latent dimensions of non-cognitive skills and academic achievements were positive, moderately sized and statistically significant across all ages (r ranging between 0.13 and 0.63) (Supplementary Figure 2; Supplementary Table 2b). Moderate and positive correlations were also observed when considering individual measures of non-cognitive skills, including academic interest, self-perceived ability, grit, and curiosity (see Supplementary Table 3). [Page 4 of the revised manuscript]*

13) Line 177: As mentioned, can you clarify why you test both latent-

**Queen Mary University of London**
Department of Biological and Experimental Psychology
School of Biological and Behavioural Sciences
Mile End Road
London E1 4NS

factor composites and individual measures, especially since several latent-factor indirect effects (e.g. age-9 self-report with the non-cognitive PGI) were non-significant—and in some cases the PGI doesn't seem to correlate with achievement (e.g. non-cog PGI at age 9)?

*Response: Thank you for this thoughtful comment. We have clarified that the analyses now focus only on the three polygenic scores from Malanchini et al. (2024). We also now explicitly state that individual indicators were examined alongside latent factors to capture construct-specific associations and ensure interpretability where latent models showed weaker fit or nonsignificant mediation. Please see excerpt below:*

*‘ Beyond examining the role of non-cognitive skills measured as latent factors capturing shared variance across different aspects of education-specific characteristics and self-regulation, we considered individual non-cognitive measures. We conducted a set of fine-grained analyses to identify which aspects of non-cognitive skills mediated genetic effects on academic achievement across development. This offered a more detailed view of construct-specific associations and reduced the risk of overinterpreting the results emerging from broad latent dimensions. First, we observed that specific student-centred academic attitudes and perceptions, such as academic interest, curiosity, and perceived ability, evinced stronger mediation effects as compared to perceptions of the learning environment that were external to each individual, such as perception of the classroom or the learning environments. These findings align with previous work, which emphasised the critical role of intrinsic, student-centred non-cognitive skills (e.g., intellectual curiosity and academic self-concept) in gene-environment transactions (40,44), driving students to actively engage in academic settings beyond what environmental factors alone can achieve (45).’ [Page 11 of the revised manuscript]*

14) Line 190-193: While several NCSs (e.g. math interest, curiosity, academic self-concept) show substantial indirect effects, many of the measures at age 16 do not show significant mediation—yet this narrative overlooks these null findings. To avoid giving a skewed impression, please revise.
Figure 2: The bottom left panel lists several age 16 measures (optimism, openness and conscientiousness) that are not mentioned elsewhere in the paper and so appear entirely new. Please clarify.

*Response: We thank the reviewer for these helpful comments. We have revised the relevant paragraph to acknowledge non-significant mediation effects more explicitly, noting that some measures—particularly broader school and classroom measures—did not show significant effects*

*‘However, not all longitudinal pathways yielded significant effects. For example, some measures on broader school and external perceptions, such as measures of*

**Queen Mary University of London**
Department of Biological and Experimental Psychology
School of Biological and Behavioural Sciences
Mile End Road
London E1 4NS

*the classroom environment at age 9 and maths environment at age 12.' [Page 6 of the revised manuscript]*

15) Line 221: Malanchini et al. (2024) states future work should explicitly investigate developmental change at the phenotypic, genetic and genomic level, suggesting latent growth curve models (LGCM). The current manuscript uses a simple time-lagged longitudinal model to assess how early skills mediate later polygenic score effects. Can the rationale for this choice of analytical approach over LGCM be provided?

*Response: We thank the reviewer for this suggestion. We agree that latent growth curve models (LGCMs) offer a powerful framework for examining developmental change, and Malanchini et al. (2024) highlighted their value for tracing dynamics developmental processes over time. However given the inconsistent measurement of non-cognitive skills across developmental stages, we were unable to model developmental trajectories. Nevertheless, we adopted a time-lagged mediation approach to test whether earlier non-cognitive skills mediated genetic effects on subsequent academic outcomes.*

16) Line 256: Can Supp Tables 14-16 be provided as a figure (akin to Fig 2). For example, table 16 has 4,700 rows making it very difficult to interpret.

*Response: We appreciate the reviewer's suggestion. Given the size and detail of Supplementary Tables 15–17 (e.g., Table 17 includes over 4,700 rows), we opted to retain them in tabular form to ensure full transparency. While we do not present these tables as figures, we have described the general patterns in the main text and highlighted key findings in more concise supplementary tables. We believe this approach balances clarity with comprehensive reporting.*

17) Line 360: Can you please use PGI/PGS, not genetics (as you are examining PGS that only capture some of the heritability.

*Response: We have revised them in the main text, please see excerpt below:*

*'We examined whether the mediating role of non-cognitive skills was more strongly linked to cognitive or non-cognitive polygenic scores. We found that mediation effects were comparable when separating education-associated genetic effects into cognitive and non-cognitive polygenic signals.' [Page 12 of the revised manuscript]*

18) Methods

Queen Mary University of London
**Queen Mary University of London**
Department of Biological and Experimental Psychology
School of Biological and Behavioural Sciences
Mile End Road
London E1 4NS

Line 444: The numbers reported here differ to those reported in Supp note 1 and Supp table 1?

*Response: We thank the reviewer for pointing this out. The analytic subsample comprised 5,016 individuals (including 2,895 DZ sibling pairs). Descriptive statistics are reported for all genotyped participants, with varying sample sizes (1,293–5,016). We now clarified this in the revised manuscript:*

*'…, resulting in a variable sample size ranging from 1,293 to 5,016 due to the inclusion of different variables.' [Page 14 of the revised manuscript]*

19) Line 486: Age 12 only has teacher report, not all three raters?

*Response: We thank the reviewer for the helpful observation. At age 12, education-specific non-cognitive skills were assessed only through self-reports. We now clarified this in the revised manuscript, please see excerpt below:*

*' At age 12 data on education-specific non-cognitive skills were collected from self-reports.' [Page 15 of the revised manuscript]*

TYPOS

20) 258 child, rather than shared by siblings, were more likely to be mediated *BY parents' perceptions

*Response: We thank for the reviewer for pointing this out, and we have modified the revised draft accordingly, see excerpt below:*

*'…child, rather than shared by siblings, were more likely to be mediated by parents' perceptions of non-cognitive profiles unique to each child...' [Page 9 of the revised manuscript]*

21) 353 emphasised the critical role of intrinsic, student-*CENTRED non-cognitive skills (e.g., intellectual curiosity and academic self-concept) in gene-environment transactions

*Response: We thank for the reviewer for pointing this out, and we have revised accordingly, see excerpt below:*

*'…emphasised the critical role of intrinsic, student-centred non-cognitive skills…'[Page 12 of the revised manuscript]*

**Queen Mary University of London**
Department of Biological and Experimental Psychology
School of Biological and Behavioural Sciences
Mile End Road
London E1 4NS

Reviewer #5 (Remarks to the Author):

I co-reviewed this manuscript with one of the reviewers who provided the listed reports. This is part of the Nature Communications initiative to facilitate training in peer review and to provide appropriate recognition for Early Career Researchers who co-review manuscripts.

*Response: We thank both the reviewer and co-reviewer for their constructive feedback, which has helped us strengthen the manuscript.*

London, 22nd January 2026

The Editorial Team
Nature Communication
Subject: Article submission.

Dear Editors,

Thank you for the opportunity to revise and resubmit our manuscript: "***From genetic disposition to academic achievement: The mediating role of non-cognitive skills across development***" to *Nature Communications*.

We are grateful for the positive feedback received from four reviewers and we have addressed additional comments provided by a fifth reviewer in this revised version of the manuscript. Please find below a point-by-point response letter.

Sincerely,
Quan Zhou and Margherita Malanchini

School of Biological and Behavioural Sciences
Queen Mary University of London

---

**REVIEWER COMMENTS**

Reviewer #1 (Remarks to the Author):

I have reviewed the revised manuscript and the accompanying response to reviewers. The authors have done an excellent and thorough job of addressing all of my comments, as well as those raised by the other reviewers. The revisions have substantially strengthened the manuscript, and my previous concerns have been fully resolved.

I am now satisfied with the manuscript in its current form and have no further substantive comments or requests for revision. This paper fills a critical gap in our knowledge of potentially modifiable pathways though which genetic effects may be mediated across development, and I am happy to recommend the paper for publication.

Chloe Austerberry

*We thank Dr. Austerberry for her positive and constructive evaluation of our revised manuscript. We are grateful for her careful review and valuable feedback. We are pleased that our revisions have fully addressed her concerns and that she considers the manuscript suitable for publication.*

Reviewer #2 (Remarks to the Author):

Thank you for the clear responses on my queries and concerns.

Congratulations on the excellent paper!

*We thank the reviewer for their positive feedback, for their careful evaluation of our work and for their constructive comments. We are pleased that our revisions have fully addressed their concerns and that they consider the manuscript suitable for publication.*

Reviewer #3 (Remarks to the Author):

NCOMMS25_19683A
I continue to see merit in this manuscript, but I am sorry to say that I am less positive than I was after my first review. This is partly because the other reviewers identified issues that I had not seen, but it is mostly because the author has not been responsive to the key issues raised by the other reviewers and myself.

1. I previously noted that one cannot refer to the TEDS sample as "representative." The author's response to my critique suggests that they think "representative" means "similar to the rest of the population." It does not. It means that every twin pair was recruited in a simple or complex design that gave each pair of twins in the population an equal chance of participation. That is not how the TEDS sample was recruited and it cannot be referred to as representative. Any sampling method other than representative sampling introduces bias into the sample, often unknowable biases.

*Response:*

*We thank the reviewer for clarifying what they mean with "representative" in this context. We have incorporated this in the revised manuscript, and below we provide a more detailed description of the TEDS recruitment process.*

*All twins born between 1994 and 1996 in England and Wales, as identified through birth records, were invited via their parents to participate. The invitations were sent to families by the UK Office for National Statistics after screening for infant mortality, and 16,810 families expressed interest in taking part. Therefore, we think that this method of recruitment would have given every twin pair in England and Wales an equal chance of*

*being included in the study. We acknowledge that recruitment of twins was dependent on their parents' interest and willingness to be involved in the study, which could have resulted in potential bias, however, this is difficult to evaluate if considering the reviewer's definition of representativeness in this context.*

*A standard way of evaluating representativeness of a cohort, is to compare participants to the wider population at the time of data collection. Below, we provide this comparison in terms of socio-economic status and ethnicity.*

*TEDS conducted the first wave of data collection when twins were 18 months old, obtaining demographic information, data about pregnancy and childbirth, and questions related to zygosity. At recruitment, TEDS was reasonably representative of the relevant England and Wales birth cohort, including in terms of ethnicity and key socioeconomic indicators. Specifically, at first contact, 91.7% of the sample was White, close to the national equivalent of ~93% for parents of young children born in the 1990s/early 2000s. The same pattern holds for parental socioeconomic indicators (e.g., parental education and employment rates). This is shown in Table 1, taken from a paper providing a detailed cohort description (Rimfeld et al., 2019).*

**Table 1.** Representativeness of TEDS sample at first contact, early childhood, middle childhood, adolescence and emerging adulthood for the full sample and separately for the genotyped sample

| | | Returned data (*N* families) | Response rate (%) | Full twin-pairs (*N*) | White (%) | Sample with 3+ full A-levels (%) | Mothers with A-levels or higher (%) | Fathers with A-levels or higher (%) | Mother employed (%) | Father employed (%) | Female (%) | MZ (%) |
|---|---|---|---|---|---|---|---|---|---|---|---|---|
| Full sample | First contact | 13,759 | 84.4 | 13,759 | 91.7 | – | 35.5 | 44.8 | 43.1 | 91.6 | 50.0 | 33.3 |
| | Early childhood | 10,336 | 70.5 | 10,336 | 92.9 | – | 37.8 | 46.3 | 43.2 | 92.2 | 50.7 | 33.8 |
| | Middle childhood | 9129 | 60.9 | 9090 | 93.0 | – | 39.9 | 47.5 | 45.9 | 92.9 | 50.8 | 35.0 |
| | Adolescence[a] | 8711 | 74.2 | 8583 | 92.8 | – | 40.6 | 48.5 | 46.6 | 93.0 | 51.8 | 35.1 |
| | Emerging[a] adulthood | 8429 | 76.5 | 8004 | 92.9 | 48.1 | 41.3 | 49.3 | 46.4 | 93.5 | 53.1 | 34.4 |
| Genotyped sample | First contact | 7019 | 99.9 | 7019 | 99.9 | – | 39.6 | 46.9 | 45.6 | 92.9 | 51.9 | 38.1 |
| | Early childhood | 6287 | 90.1 | 6287 | 99.9 | – | 40.0 | 47.1 | 45.4 | 93.2 | 52.0 | 38.1 |
| | Middle childhood | 6253 | 89.4 | 6235 | 99.9 | – | 40.6 | 47.8 | 46.6 | 93.2 | 52.0 | 38.3 |
| | Adolescence[a] | 6170 | 89.5 | 6124 | 99.9 | – | 41.7 | 48.5 | 47.1 | 93.4 | 52.7 | 37.8 |
| | Emerging[a] adulthood | 5861 | 87.7 | 5638 | 99.9 | 49.9 | 42.5 | 49.5 | 47.0 | 94.0 | 53.9 | 37.3 |
| National equivalents (for cohort of parents with young children born in late 1990s, early 2000s) | | | | | 93[c] | | 35[d] | 47[d] | 50[b,e] | 91[e] | | 33[f] |
| National equivalents (for twins themselves) | | | | | | 42.1[g] | | | | | | |

*Notes*: Early childhood refers to families who provided any data when the twins were aged 2, 3 or 4 years; middle childhood refers to families who provided any data when the twins were aged 7, 8, 9 or 10 years; adolescence refers to families who provided any data when the twins were aged 12, 14 or 16; emerging adulthood refers to families who provided any data when the twins were aged 18–23 years.
[a]Only active families were contacted.
[b]50% national equivalent refers to working mothers with their youngest child under the age of 2 in the UK; this is slightly higher than at TEDS, which is expected because TEDS families have multiple births.
[c]https://www.ons.gov.uk/ons/rel/ghs/general-household-survey/2001-edition/living-in-britain-full-report.pdf
[d]https://webarchive.nationalarchives.gov.uk/20130320225719/https://media.education.gov.uk/assets/files/pdf/sfr092000pdf.pdf
[e]https://www.ons.gov.uk/employmentandlabourmarket/peopleinwork/employmentandemployeetypes/articles/moremotherswithyoungchildrenworkingfulltime/2017-09-26
[f]http://www.multiplebirths.org.uk/media.asp
[g]https://assets.publishing.service.gov.uk/government/uploads/system/uploads/attachment_data/file/502158/SFR03_2016__A_level_and_other_level_3_results_in_England_SFR_revised.pdf

*We have added further information on how the sample was recruited in the revised manuscript, please find excerpt below:*

*"All twins born between 1994 and 1996 in England and Wales, as identified through birth records, were invited via their parents to participate. The invitations were sent to families by the UK Office for National Statistics after screening for infant mortality, and 16,810 families expressed interest in taking part. The initial data collection involved the participation of over 13,000 twin pairs, and after almost three decades, TEDS maintains an active membership of over 10,000 families. TEDS sample remains largely representative of the demographic characteristics of the UK population for their respective generation in terms of ethnicity and socio-economic status [66,67]." [page 14 of the revised manuscript]*

2. In my previous review, I raised concerns about the measures and their interpretations. I thought I was being clear at that time, but I see that I was too being polite. At the risk of being blunt, therefore, I will be more clear. There is NO measure of “self-regulation” analyzed for this paper. Any reference to self-regulation is completely misleading. It is absolutely not defensible to refer to the SDQ as a measure of “self-regulation.” It is a heterogeneous measure of behavior problems, emotional problems, and prosocial behavior. Although some economists have naively referred to SDQ scores as reflecting failures of self-regulation, that hypothesis has never been supported and almost certainly could never be confirmed. I cannot endorse a manuscript that refers to the SDQ as a measure of self-regulation.

*Response: Self-regulation is widely recognized as being complex and multi-factorial, encompassing several, partially overlapping, constructs. These include the regulation of cognition, emotions, and behaviours, and involve both top-down control mechanisms such as executive functions and cognitive control, as well as temperamental and affective processes (Nigg, 2017). The highly cited review by Nigg (2017) argues for the domain generality of self-regulatory processes, which operate across three broad areas: actions, emotions and cognition. Within this framework, regulation of action is defined as the goal-directed optimization of overt physical actions, such as ocular, motor and vocal responses. Regulation of emotion describes the processes that regulate the onset and characteristics of an emotional response, such as its magnitude, duration, and intensity. Regulation of cognition is defined as the goal-oriented modification of cognitive processes, such as attention and memory, in the absence of regulation of action or emotion (Malanchini et al., 2019). In large-scale cohort studies, a number of broad emotional and behavioural questionnaires have been considered as indicators of subsets of regulation-related functioning, rather than as direct measures of self-regulation per se. And this has also been the case in our previously published work (Malanchini et al., 2024).*

*Nevertheless, we appreciate the reviewer’s concern about referring to SDQ-based measures as “self-regulation” and to avoid this potential ambiguity, we have revised the manuscript entirely. We now refer to the SDQ measures as reflecting “emotional and behavioural functioning”, rather than self-regulation. This change has been implemented consistently throughout the revised manuscript, including figures and figure captions, see for example Figure 1 below.*

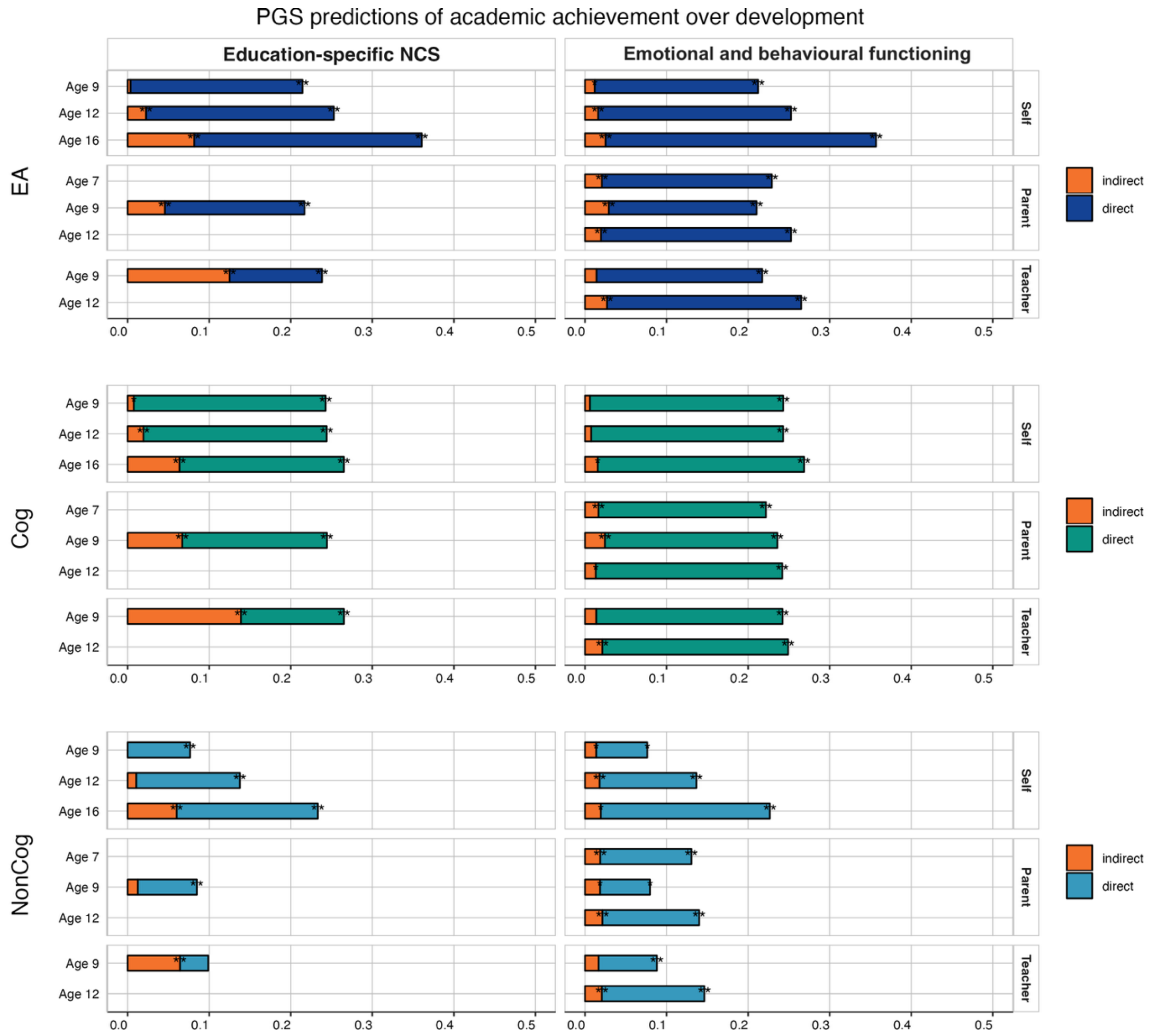


3. I was convinced by the other reviewers that the term “non-cognitive skills” is a misnomer. This is partly because constructs such as self-concept and academic interest cannot reasonably be termed “skills.” It is also because it is probably impossible to draw a clear line between cognitive and non-cognitive aspects of behavior, as cognition is part of nearly every aspect of behavior. Furthermore, at least some of the self-concept measures are likely to be results of variations in academic performance rather than mediators. I would be more comfortable with something like “non-academic characteristics.”

*Response:*

*We thank the reviewer for this comment and agree that the term “non-cognitive skills” is not without conceptual limitations. As previously noted by Reviewer 2, and now raised by Reviewer 3, many of the constructs commonly grouped under this label (e.g., academic interest or self-concept) are not “skills” in a technical sense, and it is difficult to draw a clear boundary between cognitive and non-cognitive aspects of behaviour. We also acknowledge that some measures, particularly self-concept and self-perceived ability, may partly reflect prior academic performance*

*rather than functioning as purely antecedent characteristics (Malanchini et al., 2017; Wang et al., 2020).*

*At the same time, the term “non-cognitive skills” was originally introduced to denote sources of educational variation beyond traditional cognitive ability (Heckman et al., 2006), and has since become a widely used umbrella term in both the economic and psychological literatures. We adopted this term to facilitate comparability with prior work and with existing genetically informed studies in this domain, including our laboratory’s previous research (Malanchini et al., 2024). In this sense, our use of the term is intended as a pragmatic classification rather than a strict theoretical claim about the absence of cognition or the skill-like nature of all included constructs.*

*We further acknowledge that this label encompasses a heterogeneous set of education-related characteristics, including motivational beliefs, attitudes, behavioural dispositions, and some contextually embedded perceptions, all of which has been highlighted as limitations by a recent review and meta-analysis (Smithers et al., 2018). While alternative terms such as “socio-emotional skills” or, as suggested by the reviewer, “non-academic characteristics” may address some conceptual concerns, many of the constructs examined here (e.g., academic interest, engagement, and self-concept) are tightly embedded in academic contexts and learning-related processes. This makes defining them as “non-academic” potentially ambiguous. In addition, this terminology is not widely adopted in the literature and may limit comparability with existing empirical frameworks.*

*To address these concerns, the revised manuscript explicitly acknowledges the conceptual imprecision of the term and clarifies the scope of constructs included. We should also emphasise that a central aim of the present study was to empirically evaluate which education-related non-cognitive characteristics serve as meaningful mediators of genetic associations with academic achievement, thereby informing future efforts toward a clearer and more theoretically grounded taxonomy.*

*We have revised the Discussion as follows:*

*“A further limitation concerns the heterogeneity of the constructs grouped under the umbrella of non-cognitive skills. While this label has been widely used in both the psychological and economic literature, it encompasses a diverse set of characteristics, ranging from motivational beliefs and self-regulatory strategies to attitudes and perceptions of the learning environment, that differ in their specificity, developmental stability, and contextual embedding (24,34). For instance, classroom satisfaction may reflect environmental quality as well as a dispositional characteristic. We retained the term ‘non-cognitive skills’ for consistency with prior work and to facilitate comparison with the extant literature (24,26,34), but we acknowledge its limitations. In fact, one of our goals was to empirically examine which of these constructs serve as meaningful mediators of the polygenic score prediction of academic achievement, to inform future efforts toward a clearer taxonomy of education-related non-cognitive characteristics.” [Page 13 of the revised manuscript]*

4. There are a number of typos, such as “overtime” instead of “over time” in line 102.

*Response: We have corrected the typos.*

Reviewer #4 (Remarks to the Author):

Thank you for the detailed and thorough response to our review. We appreciate the care taken to address the comments. We have no further concerns and are happy to recommend the paper for publication

Reviewer #5 (Remarks to the Author):

I co-reviewed this manuscript with one of the reviewers who provided the listed reports. This is part of the Nature Communications initiative to facilitate training in peer review and to provide appropriate recognition for Early Career Researchers who co-review manuscripts.

*We thank both reviewers for co-reviewing our manuscript and for their careful evaluation of our work. We appreciate their constructive comments and are pleased that our revision has fully addressed their concerns.*

References

Heckman, J. J., Stixrud, J., & Urzua, S. (2006). The Effects of Cognitive and Noncognitive Abilities on Labor Market Outcomes and Social Behavior. *Journal of Labor Economics*, *24*(3), 411–482. https://doi.org/10.1086/504455

Malanchini, M., Allegrini, A. G., Nivard, M. G., Biroli, P., Rimfeld, K., Cheesman, R., von Stumm, S., Demange, P. A., van Bergen, E., Grotzinger, A. D., Raffington, L., De la Fuente, J., Pingault, J.-B., Tucker-Drob, E. M., Harden, K. P., & Plomin, R. (2024). Genetic associations between non-cognitive skills and academic achievement over development. *Nature Human Behaviour*, *8*(10), 2034–2046. https://doi.org/10.1038/s41562-024-01967-9

Malanchini, M., Engelhardt, L. E., Grotzinger, A. D., Harden, K. P., & Tucker-Drob, E. M. (2019). 'Same but different': Associations between multiple aspects of self-regulation, cognition and academic abilities. *Journal of Personality and Social Psychology*, *117*(6), 1164–1188. https://doi.org/10.1037/pspp0000224

Malanchini, M., Wang, Z., Voronin, I., Schenker, V. J., Plomin, R., Petrill, S. A., & Kovas, Y. (2017). Reading self-perceived ability, enjoyment and achievement: A genetically informative study of their reciprocal links over time. *Developmental Psychology*, *53*(4), 698–712. https://doi.org/10.1037/dev0000209

Nigg, J. T. (2017). Annual Research Review: On the relations among self-regulation, self-control, executive functioning, effortful control, cognitive control, impulsivity, risk-taking, and inhibition for developmental psychopathology. *Journal of Child Psychology and Psychiatry, and Allied Disciplines*, *58*(4), 361–383. https://doi.org/10.1111/jcpp.12675

Rimfeld, K., Malanchini, M., Spargo, T., Spickernell, G., Selzam, S., McMillan, A., Dale, P. S., Eley, T. C., & Plomin, R. (2019). Twins Early Development Study: A Genetically Sensitive Investigation into Behavioral and Cognitive Development from Infancy to Emerging Adulthood. *Twin Research and Human Genetics*, *22*(6), 508–513. https://doi.org/10.1017/thg.2019.56

Smithers, L. G., Sawyer, A. C. P., Chittleborough, C. R., Davies, N. M., Davey Smith, G., & Lynch, J. W. (2018). A systematic review and meta-analysis of effects of early life non-cognitive skills on academic, psychosocial, cognitive and health outcomes. *Nature Human Behaviour*, *2*(11), Article 11. https://doi.org/10.1038/s41562-018-0461-x

Wang, Z., Oh, W., Malanchini, M., & Borriello, G. (2020). The developmental trajectories of mathematics anxiety: Cognitive, personality, and environmental correlates. *CONTEMPORARY EDUCATIONAL PSYCHOLOGY*, *61*. https://doi.org/10.1016/j.cedpsych.2020.101876

**REVIEWERS' COMMENTS**

Reviewer #3 (Remarks to the Author):

The authors responded well to my previous comments and I have no further concerns.

*We thank the reviewer for their positive feedback, for their careful evaluation of our work and for their constructive comments. We are pleased that our revisions have fully addressed their concerns.*
